# Comparative Genomics Identifies Epidermal Proteins Associated with the Evolution of the Turtle Shell

**DOI:** 10.1093/molbev/msv265

**Published:** 2015-11-24

**Authors:** Karin Brigit Holthaus, Bettina Strasser, Wolfgang Sipos, Heiko A. Schmidt, Veronika Mlitz, Supawadee Sukseree, Anton Weissenbacher, Erwin Tschachler, Lorenzo Alibardi, Leopold Eckhart

**Affiliations:** ^1^Research Division of Biology and Pathobiology of the Skin, Department of Dermatology, Medical University of Vienna, Vienna, Austria; ^2^Dipartimento di Scienze Biologiche, Geologiche ed Ambientali (BiGeA), University of Bologna, Bologna, Italy; ^3^Clinical Department for Farm Animals and Herd Management, University of Veterinary Medicine Vienna, Vienna, Austria; ^4^Center for Integrative Bioinformatics Vienna (CIBIV), Max F. Perutz Laboratories, Medical University of Vienna, University of Vienna, Vienna, Austria; ^5^Vienna Zoo, Vienna, Austria

**Keywords:** turtles, skin, gene family, integument, gene duplication.

## Abstract

The evolution of reptiles, birds, and mammals was associated with the origin of unique integumentary structures. Studies on lizards, chicken, and humans have suggested that the evolution of major structural proteins of the outermost, cornified layers of the epidermis was driven by the diversification of a gene cluster called Epidermal Differentiation Complex (EDC). Turtles have evolved unique defense mechanisms that depend on mechanically resilient modifications of the epidermis. To investigate whether the evolution of the integument in these reptiles was associated with specific adaptations of the sequences and expression patterns of EDC-related genes, we utilized newly available genome sequences to determine the epidermal differentiation gene complement of turtles. The EDC of the western painted turtle (*Chrysemys picta bellii*) comprises more than 100 genes, including at least 48 genes that encode proteins referred to as beta-keratins or corneous beta-proteins. Several EDC proteins have evolved cysteine/proline contents beyond 50% of total amino acid residues. Comparative genomics suggests that distinct subfamilies of EDC genes have been expanded and partly translocated to loci outside of the EDC in turtles. Gene expression analysis in the European pond turtle (*Emys orbicularis*) showed that EDC genes are differentially expressed in the skin of the various body sites and that a subset of beta-keratin genes within the EDC as well as those located outside of the EDC are expressed predominantly in the shell. Our findings give strong support to the hypothesis that the evolutionary innovation of the turtle shell involved specific molecular adaptations of epidermal differentiation.

## Introduction

**Fig. 1.**
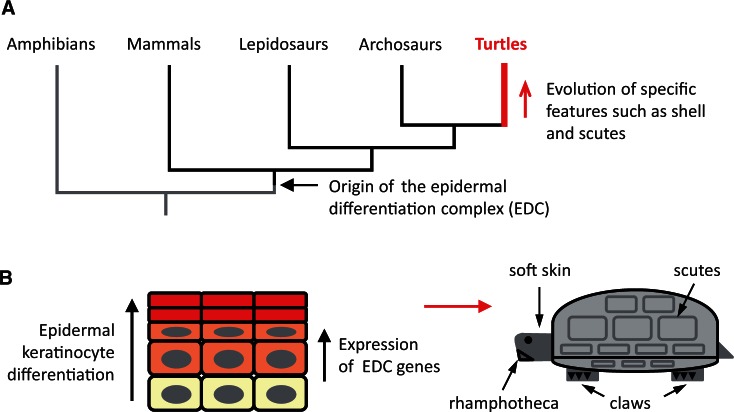
Schematic overview of the phylogenetic position of turtles and keratinocyte differentiation in the epidermis of turtles. (*A*) Phylogenetic tree of turtles and other vertebrates. (*B*) Diagram of the epidermis of turtles and other amniotes. Keratinocytes proliferate in the basal layer (yellow) and, upon transition into suprabasal layers, undergo a differentiation program that ultimately converts living cells into dead components of the cornified layer (red) (left panel). Variations of the gene expression program during differentiation lead to various epidermal structures of turtles, such as the scutes of the shell (right panel).

Turtles are a clade of reptiles that have evolutionarily diverged from their next relatives, that is, the archosaurs (crocodilians and birds) approximately 240–260 Ma (fig. 1*A*; Iwabe et al. 2005; Kumar and Hedges 2011; Shaffer et al. 2013; Wang et al. 2013; Thomson et al. 2014; Bever et al. 2015; Crawford et al. 2015). The most important morphological innovation in the evolution of turtles has been the shell which is composed of skeletal, dermal, and epidermal elements that together form the ventral plastron and the dorsal carapace (Zangerl 1969). The complex evolution and development of the bony elements of the turtle shell have been extensively studied and reviewed (Ruckes 1929; Burke 1989; Reisz and Head 2008; Nagashima et al. 2009; Hirasawa et al. 2013, 2015; Rice et al. 2015). The epidermal components of the shell are the scutes in hard-shelled turtles and the largely unpatterned epidermis in soft-shelled turtles (Thomson et al. 2014). The latter have lost both scales, an ancestral trait of reptiles, and scutes, which are generally considered to be derived from scales (Alibardi and Thompson 1999; Thomson et al. 2014). Other important epidermal structures of turtles are the claws, which are shared with other amniotes (Alibardi 2003, 2014) and the rhamphotheca, a horny sheath covering the mandibles that functionally compensates the absence of teeth in turtles. The molecular basis for the evolution of epidermal structures in turtles is only beginning to emerge (Dalla Valle et al. 2009; Li et al. 2013; Moustakas-Verho et al. 2014, 2015).

The epidermis of vertebrates is a stratified epithelium in which cells of the basal layer proliferate and start to differentiate upon detachment from the basement membrane that separates the epidermis from the underlying dermis. Keratinocyte differentiation involves the transcriptional upregulation of genes that encode structural proteins and the passive movement of cells toward the skin surface. Ultimately, keratinocytes undergo cornification, a mode of programmed cell death (Eckhart et al. 2013) that generates mechanically rigid and interconnected cell corpses (corneocytes) (fig. 1*B*). Although the molecular determinants of epidermal differentiation have been characterized only incompletely in turtles, it can be inferred from comparison with other amniotes (Strasser et al. 2014) that the epidermal features of turtles are a consequence of specific adaptations of the process of keratinocyte differentiation.

In mammals, many of the components of the cornified protein envelope of corneocytes are encoded by genes of a gene cluster known as the Epidermal Differentiation Complex (EDC) (Mischke et al. 1996). The human EDC comprises genes encoding S100A proteins, peptidoglycan recognition proteins (PGLYRP), simple EDC (SEDC) genes with one noncoding and one coding exon such as loricrin, involucrin, and small proline-rich proteins, and S100 fused-type proteins (SFTPs) such as cornulin, trichohyalin, and filaggrin (Henry et al. 2012; Kypriotou et al. 2012).

Recently, we have shown that a gene cluster with the same basic organization is also present in two sauropsidian model species, the chicken and the green anole lizard (Strasser et al. 2014). Moreover, in the above study we demonstrated that these genes are specifically expressed in epidermal keratinocytes. Loricrin contributes to the formation of the skin barrier not only in mammals but also in lizards (Strasser et al. 2014). SFTPs are expressed in human and avian epithelia that function as scaffolds for growing skin appendages such as claws, hair, and feathers (Mlitz et al. 2014). Recently, a new epidermal differentiation cysteine-rich protein (EDCRP) has been detected as a component of avian feathers (Strasser et al. 2015). Importantly, gene locus synteny (Vanhoutteghem et al. 2008; Strasser et al. 2014) and conservation of exon–intron organization (Strasser et al. 2014) have led to the hypothesis that the beta-keratins, which are widely considered the main epidermal proteins of sauropsids (Fraser and Parry 1996, 2014; Alibardi et al. 2009), have originated in the EDC and represent a sauropsid-specific subtype of SEDC gene products (Strasser et al. 2014). It is important to note that the term “beta-keratins” indicates neither common ancestry nor sequence similarity to “keratins” in the sense used by the Gene Nomenclature Committee. The latter group of proteins was originally named “alpha-keratins” and belongs to the intermediate filament protein superfamily (Schweizer et al. 2006). We advocate the renaming of beta-keratins to “corneous beta-proteins” or another term without the misleading word keratin, but we will use the traditional term here to link our report to the previous literature on skin proteins of turtles. The phylogeny of beta-keratins in turtles has been recently reported (Li et al. 2013); however, the role of the EDC in the evolution of the unique integument of turtles has remained elusive.

Here, we report the identification of the genes that constitute the EDC in turtles, the investigation of EDC gene expression in a turtle model species, and comparative analyses that suggest evolutionary trajectories for the main types of EDC genes in turtles. Our results reveal that the evolution of turtles involved expansions of gene families within the EDC, translocations of beta-keratin and other genes to novel loci outside of the EDC, and adaptations of EDC gene expression patterns to turtle-specific integumentary structures.

## Results

### The Basic Organization of the EDC Is Conserved in Turtles

To investigate the presence and organization of the EDC in a representative turtle species, we used the published genome sequence of the western painted turtle, *Chrysemys picta bellii* (Shaffer et al. 2013), and determined the set of genes located between the homologs of S100A12 and S100A11 genes. Automatic gene prediction algorithms had failed to correctly annotate many EDC genes of the chicken and lizard (Strasser et al. 2014), and were also not considered reliable for *C. picta.* Therefore, we used the existing gene annotations for S100A and PGLYRP genes only, and performed tBLASTn searches with the amino acid sequences of human, chicken, and lizard EDC-encoded proteins (Strasser et al. 2014) and predicted additional genes of the SEDC type by screening conceptual translations of the EDC nucleotide sequence. Iterative rounds of gene searches were performed in which newly predicted amino acid sequences were used as query sequences for the tBLASTn searches.

**Fig. 2.**
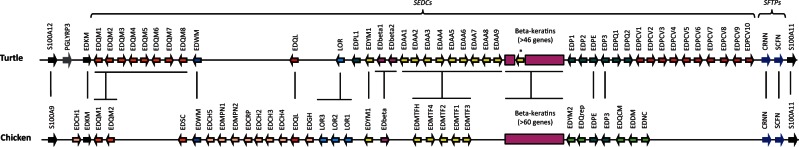
Organization of the EDC in the turtle *Chrysemys picta* in comparison to that of the chicken. Genes of the EDC in chicken (chromosome 25) and the turtle *C. picta* are schematically depicted. Arrows indicate the orientation of the genes. SEDC genes with two exons are represented by colored arrows with a black frame whereas other genes are shown as filled arrows. Clusters of beta-keratin genes are shown as boxes (for more detailed information about beta-keratins, see supplementary fig. S13, Supplementary Material online). The gene *EDAA10* (*) is located within the beta-keratin gene cluster of the turtle. Colors indicate families of genes as defined in the text. Numbers indicate the position of genes within each family cluster but not 1:1 orthology to specific members of the same gene family in other species. Black vertical lines connect orthologous genes or gene families. Note that the schemes are not drawn to scale.

The EDC of the western painted turtle has an organization of largely shared synteny with that of the chicken (Strasser et al. 2014; fig. 2). Besides 12 S100A genes and *PGLYRP3*, we identified a homolog of *EDKM*, 90 SEDC genes (including five partial genes) and 2 SFTP genes on the EDC scaffold (GenBank accession number NW_007281429.1) of the *C. picta* genome (supplementary tables S1 and S2 and fig. S1, Supplementary Material online). Names and abbreviations were tentatively assigned to these genes according to a preliminary nomenclature system for sauropsidian EDC genes (Strasser et al. 2014; supplementary table S1, Supplementary Material online). In addition to the SEDC genes on the EDC scaffold, we identified SEDC gene homologs at two genome loci outside of the EDC as well as on several short scaffolds that did not contain any other genes than SEDCs. Because the scaffold containing the great majority of EDC genes has several sequence gaps, it is possible and even likely that some of the latter scaffolds have not yet been integrated into their correct position within the EDC and that the number of genes within the EDC is higher than that on the genomic scaffold mentioned above. Details on the SEDC genes identified at non-EDC loci are provided below.

The gene loci identified in *C. picta* were compared to those of three other turtles of which genome sequences were available in GenBank, that is, *Chelonia mydas*, *Pelodiscus sinensis,* and *Apalone spinifera.* These comparisons showed a similar organization of the EDC in *C*he*. mydas* and *P. sinensis* (supplementary tables S3 and S4 and figs. S2 and S3, Supplementary Material online) whereas the fragmented genome sequence assembly of *A. spinifera* did not allow alignments of sufficient length (not shown).

### Proteins Encoded by Turtle EDC Genes Have Evolved Extreme Biases in Amino Acid Compositions and Highly Repetitive Sequences

**Fig. 3.**
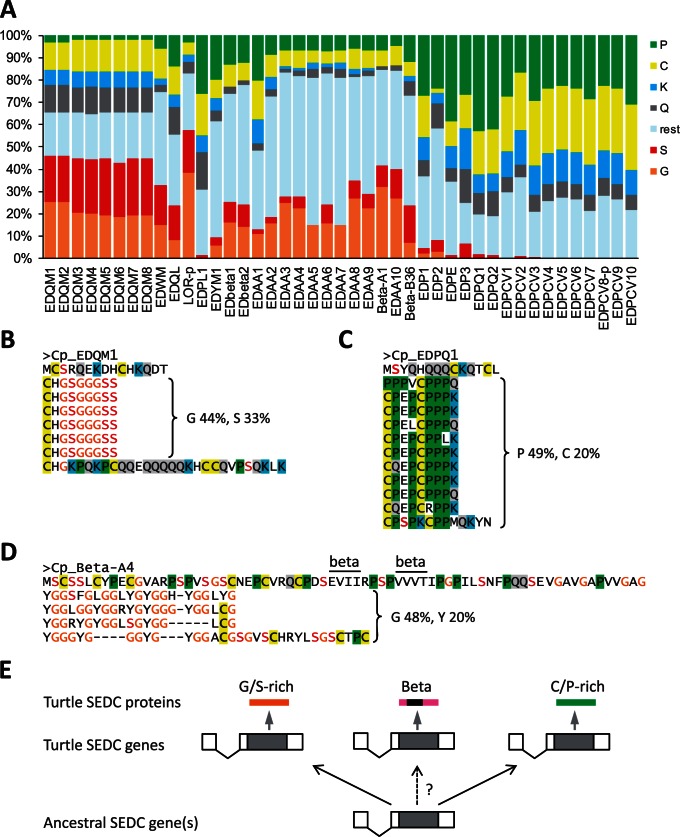
SEDC genes encode proteins with extremely biased amino acid composition. (*A*) The diagram shows the amino acid compositions of SEDC proteins of *Chrysemys picta.* The protein data are shown in the order of the corresponding genes in the EDC (fig. 2). Note that out of the main beta-keratin gene cluster, only the translation products of the first and the last gene are included here. (*B–D*) Amino acid sequences of exemplary SEDC proteins. The positions of two predicted beta-sheets in Beta-A4 are indicated. (*E*) Schematic depiction of the evolutionary diversification of SEDC genes from a common ancestral gene.

The newly identified EDC gene sequences of turtles were translated in silico (supplementary figs. S1 and S2, Supplementary Material online) and the resulting amino sequences were analyzed for features that might be associated with the presumable function of the encoded proteins in the epidermis of turtles. As previous studies have suggested that the evolution of the EDC has generated SEDC proteins with highly diverse amino acid compositions (Strasser et al. 2014), we determined the amino acid contents of SEDC proteins in *C. picta.* Indeed, many SEDC proteins of *C. picta* have extremely high contents of either glycine and serine, or cysteine and proline (fig. 3*A*–*C*), and, in addition, contain lysine and glutamine residues which are supposed to be the sites of protein cross-linking via transglutamination (Strasser et al. 2014). Remarkably, the combined content of cysteine and proline exceeded 50% of the total amino acid residues in several SEDC proteins. The genes encoding glycine/serine-rich proteins were clustered in one half (fig. 2) of the EDC whereas the genes encoding cysteine/proline-rich proteins were clustered in the other half (fig. 2) of the EDC, indicating that they arose by tandem duplication events. Another group of genes encoding proteins rich in aromatic amino acids, particularly histidine and tyrosine (supplementary fig. S4, Supplementary Material online), is located in the central region of the EDC. These genes are likely homologous to chicken genes that were previously named “epidermal differentiation proteins starting with the MTF motif” (EDMTFs) (Strasser et al. 2014). For the turtle homologs of EDMTFs, we propose the name epidermal differentiation proteins rich in aromatic amino acids (EDAAs). Beta-keratins, as defined by the presence of a 34-amino acid residue segment with high propensity to form beta-sheets (Fraser and Parry 1996, 2014; Alibardi et al. 2009), are encoded by SEDC genes located on both sides of the EDAA cluster. The amino-terminal portion of most beta-keratins does not have an extreme bias in the amino acid content whereas the carboxy-terminal portion is typically rich in glycine and tyrosine (fig. 3*D*).

Among the two SFTPs of *C. picta*, cornulin is rich in proline (18%), glutamine (10%), and glutamic acid (14%) whereas scaffoldin is rich in glutamic acid (∼24%), arginine (∼22%), and proline (∼18%; the percentage numbers are not accurate because the gene has not been completely sequenced). In many SEDC proteins (fig. 3*B* and *C*) and in both SFTPs (supplementary fig. S5, Supplementary Material online), the amino acid sequences are dominated by repeats, possibly representing the products of inequal crossovers during the evolution of EDC genes (Strasser et al. 2014). Proteins encoded by genes at various positions distributed over the entire length of the SEDC gene cluster of *C. picta* contain conserved sequence motifs at their amino and carboxy-terminus (supplementary fig. S6, Supplementary Material online), similar to diverse proteins encoded by EDC genes of humans, chicken, and lizard (Strasser et al. 2014). The conservation of lysine and glutamine residues, that is, the target amino acids of transglutamination (Strasser et al. 2014), suggests that protein cross-linking via transglutamination is a conserved feature of EDC proteins. Common exon–intron structure, a gene arrangement compatible with an evolution by tandem duplications, and the presence of conserved sequence elements at the amino- and carboxy-termini of many (but not all, e.g., beta-keratins) SEDC proteins, support the hypothesis that SEDC genes have originated from a single or only few ancestral gene(s) (Strasser et al. 2014). The amino acid sequences of turtle SEDC proteins exemplify the remarkable sequence diversification that has accompanied the evolution of epidermal proteins in amniotes (fig. 3*E*).

### Gene Duplications and Translocations Have Generated Families of SEDC Genes Both Inside and Outside the EDC of Turtles

To allow for hypotheses on the evolutionary history of individual EDC genes of turtles, we next compared the amino acid sequences of proteins encoded by genes along the EDC. Classical approaches of molecular phylogenetics were deemed not applicable for most EDC genes because of the highly repetitive nature of amino acid sequences and because of the biased amino acid compositions of the encoded proteins, which precluded unambiguous sequence alignments. However, we performed a phylogenetic analysis of beta-keratins (see below).

We found that a large portion of the EDC of *C. picta* was comprised by five distinct gene types, namely those encoding EDQMs (Epidermal Differentiation proteins containing a glutamine (Q) Motif) (supplementary fig. S7, Supplementary Material online), EDAAs (supplementary fig. S8, Supplementary Material online), EDP (Epidermal Differentiation proteins rich in Proline)-like proteins, EDPCVs (Epidermal Differentiation proteins rich in Proline, Cysteine and Valine) (supplementary fig. S9, Supplementary Material online), and beta-keratins (supplementary fig. S10, Supplementary Material online). Only the existence of the latter proteins of turtles and their homology to proteins of the chicken was reported previously (Dalla Valle et al. 2009; Li et al. 2013). Orthologs of *EDQM*, *EDAA*,** and *EDP*-like genes are also present in the chicken, whereas turtle *EDPCV* genes appear to lack counterparts in the chicken (fig. 2).

The number of *EDQM* genes was higher in *C. picta* (*n* = 8) than in chicken (*n* = 2), suggesting a lineage-specific expansion of this gene family. Similarly, the number of *EDAA* genes in *C. picta* (*n* = 22) was higher than the number of the homologous *EDMTF* genes in the chicken (*n* = 5). Unexpectedly, BLAST searches identified a locus (between genes encoding SLAMF8 and NLRPs) outside of the EDC that contained *EDAA* genes (supplementary fig. S11, Supplementary Material online). This locus was conserved in *Che. mydas* and *P. sinensis*, however in the latter only *EDAA* genes carrying premature stop codons or frameshift mutations could be identified. This pattern of *EDAA* gene loci is compatible with the hypothesis that *EDAA* genes originated within the EDC, and *EDAA* copies were translocated next to the *SLAMF8* locus (supplementary fig. S11, Supplementary Material online) in the stem lineage of turtles. Fifteen *EDPCV* genes were identified in *C. picta*, whereas only four *EDPCV* genes were found in the soft-shelled turtle *P. sinensis.* In the latter we identified a scaffold (GenBank accession number NW_005854374.1) that contained *EDPCV* genes as well as the gene *Natural killer cell receptor 2B4-like*, suggesting that this scaffold is not part of the EDC. As neither *C. picta* nor *Che. mydas* had *EDPCV* genes at syntenic loci, it is likely that the *EDPCV* gene cluster has undergone a rearrangement, possibly a translocation of a subset of its genes, in *P. sinensis.*

The largest family of SEDC proteins of the turtles are the beta-keratins. In total, we identified 82 complete and more than 10 partial beta-keratin genes in the genome of *C. picta.* Sequence alignments showed that there were subfamilies with characteristic sequence motifs (supplementary fig. S10, Supplementary Material online). Comparisons of beta-keratin gene loci of *C. picta*, *Che. Mydas*, and *P. sinensis* and genomes of other vertebrates demonstrated that some of the beta-keratin genes of the turtles are located adjacent to the gene *ODF3B* outside of the EDC (supplementary fig. S12, Supplementary Material online). No other vertebrates have beta-keratin genes at this locus, suggesting that this beta-keratin gene cluster originated specifically in the evolutionary lineage leading to modern turtles. The beta-keratins encoded by genes at this locus (tentatively named Beta-O proteins, whereby O indicates the location of the genes “outside of the EDC”), are most closely related to beta-keratins encoded by a subcluster (tentatively named Beta-A) of the beta-keratin gene cluster in the EDC (supplementary fig. S13, Supplementary Material online). Within the EDC, the Beta-A gene cluster is flanked by the Beta-B cluster of beta-keratins for which we could not identify close homologs outside of the EDC. The cluster of Beta-A genes of the turtle is syntenic to “claw beta-keratins” (figure 3 in Greenwold et al. 2014) of the chicken (designated “Beta claw” in supplementary fig. S13*A*, Supplementary Material online). Phylogenetic analysis suggests that beta-keratins of the Beta-A plus Beta-O clade of turtles and claw, feather, and scale beta-keratins of the chicken form four separate strongly supported monophyletic groups. Furthermore, these groups cluster together to the exclusion of the other beta-keratins (supplementary fig. S13*B*, Supplementary Material online). Together with the localization of Beta-A genes within the phylogenetically ancient beta-keratin subcluster of the EDC (supplementary fig. S13, Supplementary Material online), the strong support for the joined subtree of Beta-A and Beta-O proteins suggests that the cluster of Beta-O genes arose by translocation of one or more ancestral genes from the Beta-A gene cluster, followed by gene duplications.

In addition to the above-mentioned gene families, the EDC of turtles contains several individual genes that are orthologous to EDC genes of the chicken and other amniotes (Strasser et al. 2014). Like the EDCs of the lizard and human but different from that of the chicken, the turtle EDC contains a *PGLYRP3* gene. The western painted turtle has a single *LOR* gene (fig. 2, supplementary fig. S3, Supplementary Material online) whereas the chicken has three (Strasser et al. 2014). Both in turtle and chicken, *LOR* is flanked by a gene, tentatively named *EDQL* (previously named *EDQM3* in chicken (Strasser et al. 2014)), that encodes a protein with a carboxy-terminus highly similar to that of loricrin (supplementary fig. S14*A* and S6 and table S1, Supplementary Material online). *EDWM*, an SEDC gene present in all sauropsids investigated so far (Strasser et al. 2014) is conserved in the hard-shelled turtles *C. picta* and *C*he*. mydas* but has acquired mutations that destroy its open reading frame in the soft-shelled turtles *P. sinensis* and *A. spinifera* (supplementary fig. S15, Supplementary Material online). *EDCRP* (Strasser et al. 2015) and other genes encoding extremely cysteine-rich proteins are absent between the *EDWM* and *LOR* genes of the turtle whereas they are present at this site of avian EDCs (fig. 2). *EDP3* genes were identified in *C. picta* and chicken (supplementary fig. S14*B*, Supplementary Material online). Most of the SEDC genes of *C. picta* had orthologs with highly conserved sequences in *Che. mydas* and *P. sinensis* (supplementary fig. S16, Supplementary Material online). However, the numbers of genes in the SEDC subfamilies of *EDQM* and *EDPCV* genes differed (supplementary fig. S3, Supplementary Material online), and SEDC genes containing multiple internal sequence repeats, such as *LOR* and *EDPE*, could not be faithfully predicted for *C*he*. mydas* and *P. sinensis* because of uncertainties in the genomic sequence assembly (supplementary fig. S3, Supplementary Material online, and data not shown). Thus, the evolution of individual EDC genes in the diverse subclades of turtles remains to be investigated in future studies.

Together, these data suggest that the EDC genes underwent differential evolution in the lineages leading to turtles and other sauropsids, with many genes being conserved and some genes undergoing repeated rounds of tandem duplication events to give rise to turtle-specific expansions of gene families.

### EDC Genes Are Differentially Expressed in the Shell and Other Integumentary Structures of the European Pond Turtle

To test whether the predicted EDC genes are expressed, we investigated RNA-seq data of *C. picta* and *P. sinensis* (available in the National Center for Biotechnology Information (NCBI) databases, Materials and Methods) and screened the published transcriptome sequence reads of the red-eared slider turtle (*Trachemys scripta*) (Kaplinsky et al. 2013). The available RNA-seq information from *C. picta* did not include specific samples from skin, nevertheless we found sequence reads indicating expression of the predicted exons of *EDP3*, *EDPQ1*/*2,* and two *EDPCV* genes (Shaffer et al. 2013) (supplementary table S2*A*, Supplementary Material online). RNA-seq data from *P. sinensis* (Wang et al. 2013) demonstrated expression of most predicted EDC genes (supplementary table S4*A*, Supplementary Material online) and suggested transcriptional upregulation of these genes during the developmental maturation of the epidermis (supplementary fig. S17, Supplementary Material online). The analysis of the transcriptome data from *T. scripta* (Kaplinsky et al. 2013) confirmed expression of homologs of all genes investigated, including *cornulin*, *scaffoldin*, *EDKM*, *loricrin*, *EDQL*, and *EDPE* in the embryo of *T. scripta.* However, these data did not allow assigning the transcripts to particular tissues and body sites.

**Fig. 4.**
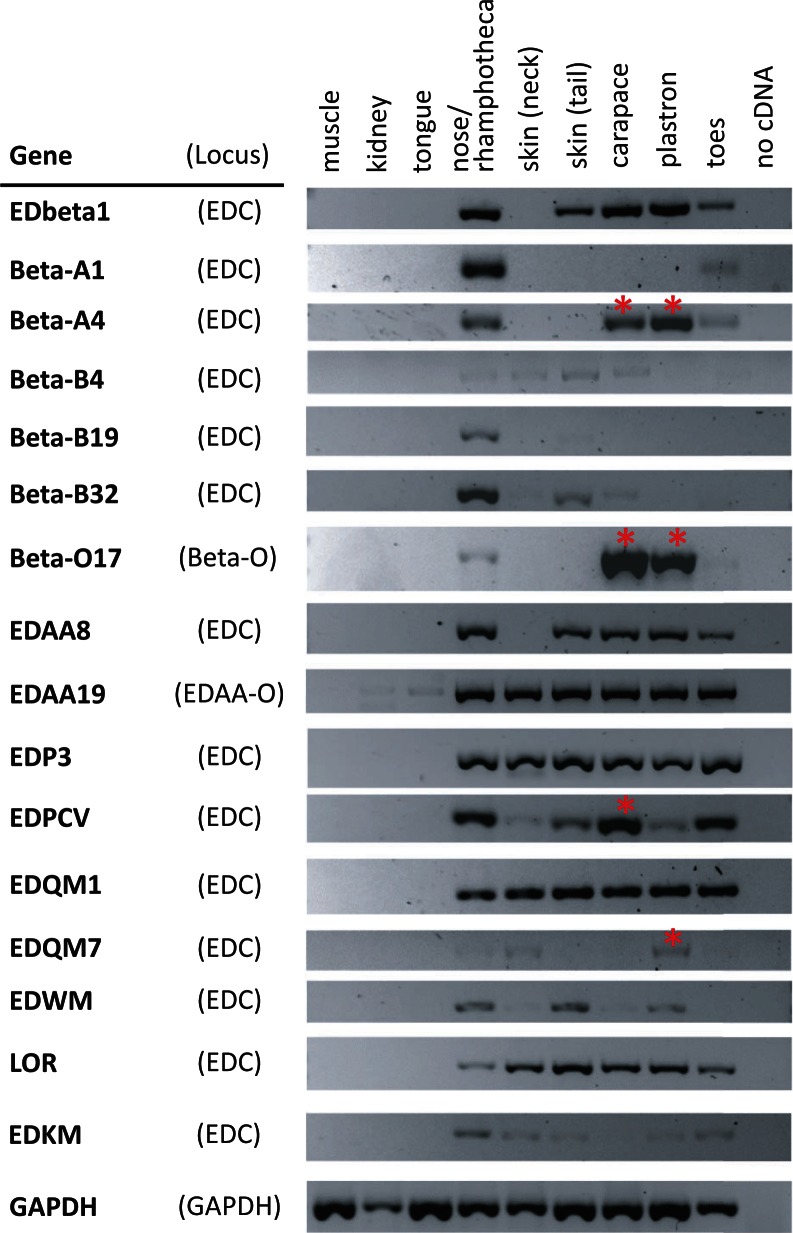
EDC genes are differentially expressed in the skin of different body sites of the European pond turtle. The expression of EDC genes was determined by RT-PCR in embryonic tissues of the European pond turtle (*Emys orbicularis*). Intron-spanning primers were designed using the sequences of the EDC genes of *Chrysemys picta* and *Chelonia mydas.* The RT-PCR products were sequenced and their identity was determined by identifying the best sequence matches with EDC genes of *C. picta* (supplementary fig. S19, Supplementary Material online). Red asterisks mark transcripts that are predominantly expressed in the shell (carapace and/or plastron).

Therefore, we studied EDC gene expression in freshly prepared turtle tissues. Because *C. picta* was not available to us, 45-days old embryos of the European pond turtle (*Emys orbicularis*) from a breeding program at the Vienna Zoo were investigated. Representative histological images illustrating the epidermal layers and fully cornified skin structures present at this embryonic stage are shown in supplementary figure S18, Supplementary Material online, Supplementary Material online. Muscle, kidney, tongue (without cornifying keratinocytes), and nose/rhamphotheca, skin of neck, tail, toes including claws, carapace, and plastron (with cornifying keratinocytes) were subjected to RNA extraction and reverse transcription polymerase chain reaction (RT-PCR) analyses using primers that were designed to anneal to the predicted exons 1 and 2 of EDC genes of *C. picta*. With the exception of primers specific for *EDPE*, all the other PCRs that we performed on the cDNAs derived from different tissues of *E. orbicularis* gave single products that could be purified and sequenced (supplementary fig. S19*A* and *B*, Supplementary Material online). Alignment of cDNA sequences of *E. orbicularis* to the predicted mRNA sequences of *C. picta* confirmed the specificity for the intended targets and revealed a high degree of sequence conservation between *E. orbicularis* and *C. picta* (supplementary fig. S19*C*, Supplementary Material online). A PCR with primers specific for the housekeeping gene *GAPDH* confirmed that all preparations of tissue samples contained cDNAs accessible for PCR amplification, though differences in cDNA amounts allowed only for semiquantitative comparisons of gene expression (fig. 4, lowermost panel). A cDNA preparation from the nose and rhamphotheca (rhinotheca) of the turtle embryos contained transcripts of all the genes investigated whereas other tissues contained only transcripts of a subset of genes. The physiological significance of the broad gene expression in the skin of the nose and/or rhamphotheca is unknown.

All genes localized in the EDC were expressed in tissues that contained epidermal keratinocytes (fig. 4). Likewise, *EDAA* genes located outside the EDC (*EDAA-O*) (supplementary fig. S11, Supplementary Material online) and beta-keratin genes outside the EDC (*Beta-O*) (supplementary fig. S12, Supplementary Material online) were essentially confined to tissues in which keratinocytes cornify (fig. 4). Transcripts of several EDC genes (*LOR*, *EDQM1*, *EDP3*, *EDAA19*) were detected in the skin of all body sites whereas some genes showed differential expression at the various regions of the body surface. Among beta-keratins, EDbeta1 showed a relatively wide expression pattern whereas Beta-A1 was expressed only in the nose/mouth region and the toes, perhaps indicating a role in the hard cornification of the rhamphotheca and the claws, respectively. Intriguingly, the transcripts tentatively named Beta-A4, originating from a gene within the Beta-A subcluster of the beta-keratin gene cluster of the EDC (supplementary fig. S13*A*, Supplementary Material online), and Beta-O17, which corresponds to a beta-keratin located outside the EDC, were present at the highest levels of expression in the carapace and the plastron. In particular, Beta-O17 was essentially specific for the shell because RT-PCR products from the nose/rhamphotheca and the toes were much weaker than those from the carapace and the plastron (fig. 4, uppermost panel). In summary, the expression analysis of EDC and EDC-related genes of *E. orbicularis* demonstrated that most genes are differentially expressed at various body sites and some of these genes, including beta-keratins of the Beta-A and Beta-O families as well as distinct SEDC genes different from beta-keratins, are expressed predominantly in the shell (fig. 4, red asterisks).

## Discussion

**Fig. 5.**
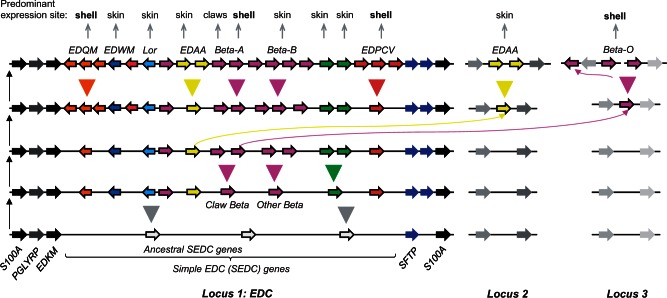
A scenario for the evolution of the EDC in turtles. Based on the results of this study a scenario for the diversification of turtle EDC genes was developed. The hypothetical structures of the EDC and two other loci, that contain EDC-related genes in modern turtles, are depicted schematically. The most primitive EDC containing ancestral SEDC genes (“simple EDC genes” consisting of one noncoding and one coding exon) is shown at the bottom. The association of EDC gene expression with tissues of modern turtles, as determined by RT-PCRs, is shown on the top of the schematics. Genes are represented by arrows. Curved lines indicate gene translocations; triangles indicate gene family expansions. To provide a better overview, only a subset of EDC genes of each clade (indicated by different colors) is shown.

The results of this study suggest that the evolution of the unique morphology of turtles involved specific adaptations of epidermal differentiation genes located in, or originating from the amniote-specific gene cluster known as EDC (Strasser et al. 2014). A scenario for the evolution of the EDC in turtles is schematically depicted in figure 5. According to this model, the basic organization of the EDC was inherited from a common ancestor of turtles and their next relatives, the archosaurs. In the lineage leading to turtles, *EDAA* and beta-keratin genes were independently translocated to loci outside the EDC. The *EDQM* and *EDPCV* gene families as well as *EDAA* and beta-keratin genes both within and outside the EDC expanded by repeated gene duplications. Furthermore, many EDC genes acquired differential expression patterns in various skin structures. We propose that some EDC genes, including a subset of beta-keratin genes (members of the Beta-A cluster), and beta-keratin genes at the locus outside of the EDC (Beta-O) evolved a predominant expression in scales of the dorsal and ventral aspects of the body where they contributed to the evolution of the hard scutes of the shell.

EDC genes encode structural proteins of epidermal keratinocytes (Henry et al. 2012; Kypriotou et al. 2012; Eckhart et al. 2013). In particular, proteins encoded by SEDC genes are supposed to exert their function by becoming cross-linked components of mechanically resilient structures at the skin surface (Candi et al. 2005; Eckhart et al. 2013). The relative abundance and the type of molecular interactions of individual proteins likely modulate the physicochemical parameters of cornification products such as the pliable cornified layer of the “soft” epidermis and the more rigid scutes of the shell. Our data suggest that SEDC protein families with very different amino acid contents have expanded during the evolution of turtles, namely EDQMs (containing a characteristic stretch of glutamine residues), EDPCVs (rich in proline and cysteine residues), EDAAs (rich in aromatic amino acids), and beta-keratins. The distinct sequence features of these protein families might facilitate different types of interactions with other structural proteins of cornifying keratinocytes, including keratins, cytolinkers, and cell junction proteins that are encoded by genes at loci outside of the EDC (Niessen 2007; Vandebergh and Bossuyt 2012; Wiche et al. 2015). Glutamine and cysteine residues (present in EDQMs and EDPCVs) are the main sites of intermolecular cross-linking of EDC proteins via transglutamination and disulfide bond formation, respectively (Kalinin et al. 2002; Eckhart et al. 2013; Rice et al. 2013). Stretches of glycine residues, located between transglutamination sites of EDQM proteins possibly allow for flexible changes in protein length that are supposed to contribute to the compaction of the cellular protein envelope during keratinocyte cornification (Candi et al. 2005). Aromatic amino acid residues (highly abundant in EDAAs and in the carboxy-terminal portion of beta-keratins) are potential sites of the non-covalent protein interaction mode termed pi-stacking (McGaughey et al. 1998; Waters 2002). Together with the emerging data on EDC proteins of other amniotes (Henry et al. 2012; Strasser et al. 2014; our unpublished data), the results of the present study provide the basis for theoretical and experimental studies on the molecular interactions that determine the epidermal phenotypes of amniotes.

The expression of EDC genes at the various body sites of turtles was investigated by semiquantitative RT-PCR analyses using *E. orbicularis* as a model species. This approach had several limitations such as the restricted availability of tissue samples which did not allow the analysis of biological replicates. Nevertheless, our results allow the conclusion that many turtle EDC genes are expressed in the skin of more than one body site. This is true for beta-keratins of the cluster B (within the EDC), loricrin, EDP3, EDAA, and at least one EDQM gene. However, our data also identify EDC genes expressed predominantly in the shell (*Beta-A4*) and, in some cases, predominantly in the carapace (*EDPCV*, assignment of this *E. orbicularis* RT-PCR product to an individual *EDPCV* gene family member was not possible) or the plastron (*EDQM7*) (fig. 4). The association of gene expression with the shell was most obvious for two beta-keratins investigated, one belonging to the Beta-A cluster (within the EDC) and the other belonging to the Beta-O cluster (outside the EDC). These findings suggest a specific role for these beta-keratins in the scutes of the shell but also indicate that other SEDC genes have contributed to the evolution of the shell.

The data presented here complement and extend previous studies on the roles of beta-keratins in the evolution of turtles. Beta-keratins, also referred to as corneous beta-proteins (Alibardi et al. 2009) to indicate their lack of common ancestry with keratins (Schweizer et al. 2006), are encoded by genes of the SEDC-type (one noncoding and one coding exon) (fig. 3*E*). They are defined by a central segment of amino acids that are predicted to form beta-sheets which mediate the formation of filaments (Fraser and Parry 1996, 2014). The conserved presence of beta-keratin genes within the SEDC gene clusters of lizard (Strasser et al. 2014), birds, and turtles as well as identical exon–intron structures of beta-keratin and other SEDC genes argue for an evolutionary origin of beta-keratins by derivation from a common ancestral gene. However, the lack of SEDC-typical sequence motifs (supplementary fig. S6, Supplementary Material online) at the amino- and carboxy-terminal ends and the presence of the beta-sheet-forming core sequence makes beta-keratins unique among SEDC proteins and leaves open the possibility that as-yet-unknown recombination events were involved in the origin of beta-keratins. Our semiquantitative RT-PCRs suggest that the Beta-A cluster of turtle beta-keratin genes comprises genes (e.g., *Beta-A1*) that are expressed in the toes and others (e.g., *Beta-A4*) that are also expressed in the toes but more strongly in the shell (fig. 4). Notably, the Beta-A cluster is syntenic with the claw beta-keratin gene cluster in birds (Greenwold et al. 2014; supplementary fig. S13*A*, Supplementary Material online), and phylogenetic analysis suggests that these genes belong to the same subclade of beta-keratins, which comprises Beta-A plus Beta-O proteins of turtles and claw, feather, and scale beta-keratins of the chicken (supplementary fig. S13*B*, Supplementary Material online). Based on these data, we put forward the hypothesis that turtle Beta-A proteins and chicken claw beta-keratins have probably been inherited from a common ancestor of turtles and birds in which the evolutionary precursors of Beta-A proteins might have been components of claws. It is conceivable that distinct sequence features of these ancestral proteins contributed to the hardness of the claws. Later, duplicated genes of this type might have been co-opted as components of the hard scutes of the evolving shell. A gene translocation and further duplications generating the Beta-O cluster of shell beta-keratins might have been associated with the further evolution of the shell (fig. 5). This scenario is partly analogous to the evolution of the so-called “hair keratins,” that is, keratin intermediate filament proteins that likely functioned in the claws of primitive amniotes before they were co-opted as components of mammalian hair (Eckhart et al. 2008).

The above scenario of beta-keratin evolution refines the evolutionary model of a previous report (Li et al. 2013), in which “turtle-specific beta-keratins,” corresponding to beta-keratins of the Beta-A and Beta-O clusters of our study, with a putative expression in the shell have been proposed. Other reports have identified mRNAs encoding 17 individual beta-keratins in the hard-shelled turtle *Pseudemys nelsoni* (Dalla Valle et al. 2009) and five beta-keratins in the soft-shelled turtle *A. spinifera* (Dalla Valle et al. 2013). The results of the present study allow assigning 14, 2 and 1 beta-keratins of *P. nelsoni* to the Beta-O, A and B clusters, respectively, whereas all previously described beta-keratins of *A. spinifera* belong to the Beta-B cluster (supplementary fig. S20, Supplementary Material online). In agreement with our RT-PCR results obtained in *E. orbicularis*, the mRNA transcripts from Beta-B genes of *P. nelsoni* and *A. spinifera* tended to be more abundant in tissues outside of the shell (Dalla Valle et al. 2009, 2013). In contrast, a Beta-O protein predominated over a Beta-B protein in the scutes of the shell of *P. nelsoni* according to a recent immuno-labeling study (Alibardi 2014), supporting the role of Beta-O proteins in the shell, as proposed here. In future studies, it will be important to carefully consider the sequence similarities among the many beta-keratins and to further improve quantitative comparisons of individual beta-keratin expression levels at different body sites of turtles.

A hard shell was present in a common ancestor of all modern turtles and was lost during the evolution of soft-shelled turtles (Gaffney 1990; Li et al. 2008; Lyson et al. 2014). A significant role of beta-keratin pseudogenization in this degeneration process was previously suggested (Li et al. 2013). The present study confirms changes in the set of beta-keratins in *P. sinensis* and identifies further epidermal differentiation genes that have been lost in this soft-shelled turtle. Besides a rearrangement and reduction of the number of *EDPCV* genes in *P. sinensis*, we found an inactivation of *EDWM* in the two soft-shelled turtles *P. sinensis* and *A. spinifera.* Since *EDWM* is present in all other sauropsids investigated so far (Strasser et al. 2014; supplementary fig. S15, Supplementary Material online), the distribution of *EDWM* in amniote species correlates with that of scales, which are widely conserved in sauropsids with the exception of soft-shelled turtles (Crawford et al. 2015). Notably, scales and scutes share elements of their developmental program (Moustakas-Verho and Cherepanov 2015). Therefore, the loss of *EDWM* may have been associated—perhaps as a secondary event after the inactivation of a surface patterning mechanism—with the loss of scales and hard scutes in soft-shelled turtles. A scenario summarizing the changes of the EDC during the evolution of soft-shelled turtles is depicted in supplementary figure S21, Supplementary Material online. It will be interesting to explore the genomic foundations for the diversification of the integument in the various phylogenetic lineages of turtles in future studies.

Collectively, the results of the present comparative genomics study and our gene expression data indicate that the evolution of the integument of turtles was associated with numerous adaptations of genes involved in epidermal differentiation and with the origin and expansion of shell-associated proteins. As this study provides a comprehensive catalog of EDC genes expressed in the epidermis and distinct skin appendages of turtles, these data will facilitate further in-depth investigations of the evolution of claws, rhamphotheca, scutes, and scales of turtles, and reptiles in general.

## Materials and Methods

### Genome Sequences and Gene Identification

Genome sequences from the following turtle species were used for gene predictions: western painted turtle (*C. picta bellii*) (Shaffer et al. 2013), Chinese soft-shelled turtle (*P. sinensis*), and green sea turtle (*Che. mydas*) (Wang et al. 2013). The accession numbers of genome sequences are listed in supplementary tables S2–S4, Supplementary Material online. Coding sequences and exon–intron borders were predicted according to a published approach (Strasser et al. 2014). Briefly, the genomic regions between S100A12 and S100A11 genes were screened for EDC genes using the following three methods. First, the amino acid sequences of EDC proteins from other amniotes were used as queries in tBLASTn searches. Second, RNA-seq data available in the Sequence Read Archive and information about RNA-seq exon coverage available in the NCBI browser for “genomic regions, transcripts, and products” were used to identify transcribed regions, which were subsequently investigated for the potential to encode proteins with amino acid sequences similar to known EDC proteins. Third, for the prediction of SEDC genes, the genomic sequence was conceptually translated, and open reading frames encoding proteins of 50–500 amino acids were identified. Putative protein-coding sequences were scrutinized for the presence of a splice acceptor site at a distance of 10–30 nt upstream of the start codon and for the presence of a putative noncoding exon 1, as defined by a TATA box followed by a splice donor site at a distance of 60–90 nt. The gene predictions were validated by BLAST searches in the transcriptome of *T. scripta* (Kaplinsky et al. 2013) and by RT-PCR tests in *E. orbicularis* (see below).

### Sequence Alignment and Phylogenetic Analysis

For phylogenetic analysis, the amino acid sequences of beta-keratins of *C. picta* (supplementary fig. S1*B*, Supplementary Material online) and chicken were used. Chicken beta-keratin genes within the EDC (chromosome 25) were identified at the genomic loci indicated in supplementary table S6, Supplementary Material online, and translated in silico. Amino acid sequences of feather beta-keratins encoded by genes outside of the EDC were obtained from Ng et al. (2014). The beta-keratin sequences were aligned using Multalin (Corpet 1988) with default settings. After checking for alignment errors, only the unambiguously aligned core segment (positions 67–126 of the overall alignment, supplementary Material online: FASTA file) was used for subsequent phylogenetic analysis. A phylogenetic tree was reconstructed by maximum likelihood (ML) using IQ-TREE 1.3.8 (Nguyen et al. 2015) using the JTT + G4 model (Jones et al. 1992; Yang 1994). The evolutionary model was determined by model selection according to Posada (2008) as implemented in IQ-Tree using the Bayesian information criterion. Tree searches were performed for three different perturbation strengths (‐pers 0.5, 0.2, and 0.1) and two different stop conditions (‐numstop 200 and 400). For each pair of search options, five replicates were performed and the reconstructed tree with the highest likelihood was taken as the ML estimate. Support values were obtained by ultrafast bootstrap approximation (UFBoot) (Minh et al. 2013) with 10,000 samples in IQ-TREE. Since UFBoot support values behave like posterior probabilities (Minh et al. 2013), branches with support values of at least 90% are regarded as supported, whereas values of at least 95% are regarded as strongly supported.

### Animal Tissues

Tissues were sampled from 45 days old embryos of the European pond turtle *(E. orbicularis*) in agreement with the national laws regulating animal welfare, the guidelines of Good Veterinary Practice, and the guidelines of the Ethics committee of the Medical University of Vienna. The embryos were derived from an *E. orbicularis* breeding program at the Vienna Zoo.

### RT-PCR

RNA was prepared from tissues of *E. orbicularis* according to a published protocol (Mlitz et al. 2014; Strasser et al. 2014). The RNA was reverse-transcribed to cDNA which was subsequently amplified by PCRs with primers specific for EDC genes. The sequences of the primers were chosen to anneal to conserved regions of EDC genes predicted in the genomes of *C. picta* and *Che. mydas.* Primer sequences are listed in supplementary table S5, Supplementary Material online. PCR products were purified and sequenced. Nucleotide sequences of cDNAs were submitted to GenBank (accession numbers KR632557–KR632565).

## Supplementary Material

Supplementary figures S1–S21 and tables S1–S6 are available at *Molecular Biology and Evolution* online (http://www.mbe.oxfordjournals.org/).

Supplementary Data

## References

[msv265-B1] Alibardi L 2003 Adaptation to the land: the skin of reptiles in comparison to that of amphibians and endotherm amniotes. J Exp Zool B Mol Dev Evol. 298:12–41. 12949767 10.1002/jez.b.24

[msv265-B3] Alibardi L 2014 Immunocytochemistry suggests that the prevalence of a sub-type of beta-proteins determines the hardness in the epidermis of the hard-shelled turtle. J Exp Zool B Mol Dev Evol. 322:54–63. 24254963 10.1002/jez.b.22548

[msv265-B4] Alibardi L Dalla Valle L Nardi A Toni M 2009 Evolution of hard proteins in the sauropsid integument in relation to the cornification of skin derivatives in amniotes. J Anat. 214:560–586. 19422429 10.1111/j.1469-7580.2009.01045.x PMC2736123

[msv265-B5] Alibardi L Thompson MB 1999 Epidermal differentiation during carapace and plastron formation in the embryonic turtle *Emydura macquarii*. J Anat. 194:531–545. 10445822 10.1046/j.1469-7580.1999.19440531.x PMC1467953

[msv265-B6] Bever GS Lyson TR Field DJ Bhullar BAS 2015 Evolutionary origin of the turtle skull. Nature 525:239–242. 26331544 10.1038/nature14900

[msv265-B7] Burke AC 1989 Development of the turtle carapace: implications for the evolution of a novel bauplan. J Morphol. 199:363–378. 10.1002/jmor.1051990310 29865619

[msv265-B8] Candi E Schmidt R Melino G 2005 The cornified envelope: a model of cell death in the skin. Nat Rev Mol Cell Biol. 6:328–340. 15803139 10.1038/nrm1619

[msv265-B9] Corpet F 1988 Multiple sequence alignment with hierarchical clustering. Nucleic Acids Res. 16:10881–10890. 2849754 10.1093/nar/16.22.10881 PMC338945

[msv265-B10] Crawford NG Parham JF Sellas AB Faircloth BC Glenn TC Papenfuss TJ Henderson JB Hansen MH Simison WB 2015 A phylogenomic analysis of turtles. Mol Phylogenet Evol. 83:250–257. 25450099 10.1016/j.ympev.2014.10.021

[msv265-B11] Dalla Valle L Michieli F Benato F Skobo T Alibardi L 2013 Molecular characterization of alpha-keratins in comparison to associated beta-proteins in soft-shelled and hard-shelled turtles produced during the process of epidermal differentiation. J Exp Zool B Mol Dev Evol. 320:428–441. 23794440 10.1002/jez.b.22517

[msv265-B13] Dalla Valle L Nardi A Toni M Emera D Alibardi L 2009 Beta-keratins of turtle shell are glycine-proline-tyrosine rich proteins similar to those of crocodilians and birds. J Anat. 214:284–300. 19207990 10.1111/j.1469-7580.2008.01030.x PMC2667886

[msv265-B14] Eckhart L Dalla Valle L Jaeger K Ballaun C Szabo S Nardi A Buchberger M Hermann M Alibardi L Tschachler E 2008 Identification of reptilian genes encoding hair keratin-like proteins suggests a new scenario for the evolutionary origin of hair. Proc Natl Acad Sci U S A. 105:18419–18423. 19001262 10.1073/pnas.0805154105 PMC2587626

[msv265-B15] Eckhart L Lippens S Tschachler E Declercq W 2013 Cell death by cornification. Biochim Biophys Acta. 1833:3471–3480. 23792051 10.1016/j.bbamcr.2013.06.010

[msv265-B16] Fraser RD Parry DA 1996 The molecular structure of reptilian keratin. Int J Biol Macromol. 19:207–211. 8910061 10.1016/0141-8130(96)01129-4

[msv265-B17] Fraser RD Parry DA 2014 Amino acid sequence homologies in the hard keratins of birds and reptiles, and their implications for molecular structure and physical properties. J Struct Biol. 188:213–224. 25448888 10.1016/j.jsb.2014.10.012

[msv265-B18] Gaffney ES 1990 The comparative osteology of the Triassic turtle Proganochelys. Bull Am Mus Nat Hist. 194:1–263.

[msv265-B19] Greenwold MJ Bao W Jarvis ED Hu H Li C Gilbert MT Zhang G Sawyer RH 2014 Dynamic evolution of the alpha (α) and beta (β) keratins has accompanied integument diversification and the adaptation of birds into novel lifestyles. BMC Evol Biol. 14:249. 25496280 10.1186/s12862-014-0249-1 PMC4264316

[msv265-B20] Henry J Toulza E Hsu C Pellerin L Balica S Mazereeuw-Hautier J Paul C Serre G Jonca N Simon M 2012 Update on the epidermal differentiation complex. Front Biosci. 17:1517–1532. 10.2741/4001 22201818

[msv265-B21] Hirasawa T Nagashima H Kuratani S 2013 The endoskeletal origin of the turtle carapace. Nat Commun. 4:2107. 23836118 10.1038/ncomms3107 PMC3715867

[msv265-B22] Hirasawa T Pascual-Anaya J Kamezaki N Taniguchi M Mine K Kuratani S 2015 The evolutionary origin of the turtle shell and its dependence on the axial arrest of the embryonic rib cage. J Exp Zool B Mol Dev Evol. 324:194–207. 24898540 10.1002/jez.b.22579

[msv265-B23] Iwabe N Hara Y Kumazawa Y Shibamoto K Saito Y Miyata T Katoh K 2005 Sister group relationship of turtles to the bird-crocodilian clade revealed by nuclear DNA-coded proteins. Mol Biol Evol. 22:810–813. 15625185 10.1093/molbev/msi075

[msv265-B24] Jones DT Taylor WR Thornton JM 1992 The rapid generation of mutation data matrices from protein sequences. Comput Appl Biosci. 8:275–282. 1633570 10.1093/bioinformatics/8.3.275

[msv265-B25] Kalinin AE Kajava AV Steinert PM 2002 Epithelial barrier function: assembly and structural features of the cornified cell envelope. Bioessays 24:789–800. 12210515 10.1002/bies.10144

[msv265-B26] Kaplinsky NJ Gilbert SF Cebra-Thomas J Lilleväli K Saare M Chang EY Edelman HE Frick MA Guan Y Hammond RM 2013 The embryonic transcriptome of the red-eared slider turtle (*Trachemys scripta*). PLoS One 8:e66357. 23840449 10.1371/journal.pone.0066357 PMC3686863

[msv265-B27] Kumar S Hedges SB 2011 TimeTree2: species divergence times on the iPhone. Bioinformatics 27:2023–2034. 21622662 10.1093/bioinformatics/btr315 PMC3129528

[msv265-B28] Kypriotou M Huber M Hohl D 2012 The human epidermal differentiation complex: cornified envelope precursors, S100 proteins and the ‘fused genes’ family. Exp Dermatol. 21:643–649. 22507538 10.1111/j.1600-0625.2012.01472.x

[msv265-B29] Li C Wu XC Rieppel O Wang LT Zhao LJ 2008 An ancestral turtle from the Late Triassic of southwestern China. Nature 456:497–501. 19037315 10.1038/nature07533

[msv265-B30] Li YI Kong L Ponting CP Haerty W 2013 Rapid evolution of Beta-keratin genes contribute to phenotypic differences that distinguish turtles and birds from other reptiles. Genome Biol Evol. 5:923–933. 23576313 10.1093/gbe/evt060 PMC3673632

[msv265-B31] Lyson TR Schachner ER Botha-Brink J Scheyer TM Lambertz M Bever GS Rubidge BS de Queiroz K 2014 Origin of the unique ventilatory apparatus of turtles. Nat Commun. 5:5211. 25376734 10.1038/ncomms6211

[msv265-B32] McGaughey GB Gagné M Rappé AK 1998 pi-Stacking interactions. Alive and well in proteins. J Biol Chem. 273:15458–15463. 9624131 10.1074/jbc.273.25.15458

[msv265-B33] Minh BQ Nguyen MA von Haeseler A 2013 Ultrafast approximation for phylogenetic bootstrap. Mol Biol Evol. 30:1188–95. 23418397 10.1093/molbev/mst024 PMC3670741

[msv265-B34] Mischke D Korge BP Marenholz I Volz A Ziegler A 1996 Genes encoding structural proteins of epidermal cornification and S100 calcium-binding proteins form a gene complex (“epidermal differentiation complex”) on human chromosome 1q21. J Invest Dermatol. 106:989–992. 8618063 10.1111/1523-1747.ep12338501

[msv265-B35] Mlitz V Strasser B Jaeger K Hermann M Ghannadan M Buchberger M Alibardi L Tschachler E Eckhart L 2014 Trichohyalin-like proteins have evolutionarily conserved roles in the morphogenesis of skin appendages. J Invest Dermatol. 134:2685–2692. 24780931 10.1038/jid.2014.204 PMC4260798

[msv265-B36] Moustakas-Verho JE Cherepanov GO 2015 The integumental appendages of the turtle shell: an evo-devo perspective. J Exp Zool B Mol Dev Evol. 324:221–229. 25877335 10.1002/jez.b.22619

[msv265-B37] Moustakas-Verho JE Zimm R Cebra-Thomas J Lempiäinen NK Kallonen A Mitchell KL Hämäläinen K Salazar-Ciudad I Jernvall J Gilbert SF 2014 The origin and loss of periodic patterning in the turtle shell. Development 141:3033–3039. 25053434 10.1242/dev.109041

[msv265-B38] Nagashima H Sugahara F Takechi M Ericsson R Kawashima-Ohya Y Narita Y Kuratani S 2009 Evolution of the turtle body plan by the folding and creation of new muscle connections. Science 325:193–196. 19590000 10.1126/science.1173826

[msv265-B58] Ng CS Wu P Fan WL Yan J Chen CK Lai YT Wu SM Mao CT Chen JJ Lu MY 2014 Genomic organization, transcriptomic analysis, and functional characterization of avian α- and β-keratins in diverse feather forms. Genome Biol Evol. 6:2258–2273. 25152353 10.1093/gbe/evu181 PMC4202321

[msv265-B39] Niessen CM 2007 Tight junctions/adherens junctions: basic structure and function. J Invest Dermatol. 27:2525–2532. 17934504 10.1038/sj.jid.5700865

[msv265-B40] Nguyen LT Schmidt HA von Haeseler A Minh BQ 2015 IQ-TREE: a fast and effective stochastic algorithm for estimating maximum-likelihood phylogenies. Mol Biol Evol. 32:268–274. 25371430 10.1093/molbev/msu300 PMC4271533

[msv265-B41] Posada D 2008 jModelTest: phylogenetic model averaging. Mol Biol Evol. 25:1253–1256. 18397919 10.1093/molbev/msn083

[msv265-B42] Reisz RR Head JJ 2008 Palaeontology: turtle origins out to sea. Nature 456:450–451. 19037301 10.1038/456450a

[msv265-B43] Rice R Riccio P Gilbert SF Cebra-Thomas J 2015 Emerging from the rib: resolving the turtle controversies. J Exp Zool B Mol Dev Evol. 324:208–220. 25675951 10.1002/jez.b.22600

[msv265-B44] Rice RH Winters BR Durbin-Johnson BP Rocke DM 2013 Chicken corneocyte cross-linked proteome. J Proteome Res. 12:771–776. 23256538 10.1021/pr301036k PMC3569041

[msv265-B45] Ruckes H 1929 The morphological relationships between the girdles, ribs, and carapace. Ann N Y Acad Sci. 13:81–120.

[msv265-B46] Schweizer J Bowden PE Coulombe PA Langbein L Lane EB Magin TM Maltheis L Omary MB Parry DA Rogers MA 2006 New consensus nomenclature for mammalian keratins. J Cell Biol. 174:169–174. 16831889 10.1083/jcb.200603161 PMC2064177

[msv265-B47] Shaffer HB Minx P Warren DE Shedlock AM Thomson RC Valenzuela N Abramyan J Amemiya CT Badenhorst D Biggar KK 2013 The western painted turtle genome, a model for the evolution of extreme physiological adaptations in a slowly evolving lineage. Genome Biol. 14:R28. 23537068 10.1186/gb-2013-14-3-r28 PMC4054807

[msv265-B48] Strasser B Mlitz V Hermann M Rice RH Eigenheer RA Alibardi L Tschachler E Eckhart L 2014 Evolutionary origin and diversification of epidermal barrier proteins in amniotes. Mol Biol Evol. 31:3194–3205. 25169930 10.1093/molbev/msu251 PMC4245816

[msv265-B49] Strasser B Mlitz V Hermann H Tschachler E Eckhart L 2015 Convergent evolution of cysteine-rich proteins in feathers and hair. BMC Evol Biol. 15:82. 25947341 10.1186/s12862-015-0360-y PMC4423139

[msv265-B50] Thomson RC Plachetzki DC Mahler DL Moore BR 2014 A critical appraisal of the use of microRNA data in phylogenetics. Proc Natl Acad Sci U S A. 111:E3659–E3668. 25071211 10.1073/pnas.1407207111 PMC4156711

[msv265-B51] Vandebergh W Bossuyt F 2012 Radiation and functional diversification of alpha keratins during early vertebrate evolution. Mol Biol Evol. 29:995–1004. 22046002 10.1093/molbev/msr269

[msv265-B52] Vanhoutteghem A Djian P Green H 2008 Ancient origin of the gene encoding involucrin, a precursor of the cross-linked envelope of epidermis and related epithelia. Proc Natl Acad Sci U S A. 105:15481–15486. 18809918 10.1073/pnas.0807643105 PMC2563112

[msv265-B53] Wang Z Pascual-Anaya J Zadissa A Li W Niimura Y Huang Z Li C White S Xiong Z Fang D 2013 The draft genomes of soft-shell turtle and green sea turtle yield insights into the development and evolution of the turtle-specific body plan. Nat Genet. 45:701–706. 23624526 10.1038/ng.2615 PMC4000948

[msv265-B54] Waters ML 2002 Aromatic interactions in model systems. Curr Opin Chem Biol. 6:736–741. 12470725 10.1016/s1367-5931(02)00359-9

[msv265-B55] Wiche G Osmanagic-Myers S Castañón MJ 2015 Networking and anchoring through plectin: a key to IF functionality and mechanotransduction. Curr Opin Cell Biol. 32:21–29. 25460778 10.1016/j.ceb.2014.10.002

[msv265-B56] Yang Z 1994 Maximum likelihood phylogenetic estimation from DNA sequences with variable rates over sites: approximative methods. J Mol Evol. 39:306–314. 7932792 10.1007/BF00160154

[msv265-B57] Zangerl R 1969 The turtle shell. In: Gans C d'A Bellairs Parsons T, editors. Biology of reptilia, I. New York (NY): Academic Press p. 311–339

