# Supplementary material for: Comparative Genomics Identifies Epidermal Proteins Associated with the Evolution of the Turtle Shell
Source: Mol Biol Evol. 2015 Nov 24;33(3):726–37. doi: 10.1093/molbev/msv265 (PMC4760078; doi:10.1093/molbev/msv265)
Supplement: Supplementary Data [file supp_33_3_726__index.html]

Comparative Genomics Identifies Epidermal Proteins Associated with the Evolution of the Turtle Shell — Comparative Genomics Identifies Epidermal Proteins Associated with the Evolution of the Turtle Shell — Supplementary Data 

# Comparative Genomics Identifies Epidermal Proteins Associated with the Evolution of the Turtle Shell

## Supplementary Data

files

- Supplementary Data - zip file
