# Supplementary material for: Comparative Genomics Identifies Epidermal Proteins Associated with the Evolution of the Turtle Shell
Source: Mol Biol Evol. 2015 Nov 24;33(3):726–37. doi: 10.1093/molbev/msv265 (PMC4760078; doi:10.1093/molbev/msv265)
Supplement: Supplementary Data [file supp_msv265_suppl_data.zip › Supplementary_Tables_Holthaus-et-al.pdf]

Supplementary Data: Supplementary Tables

Comparative genomics identifies epidermal differentiation proteins associated with the evolution of the turtle shell

Karin Brigit Holthaus, Bettina Strasser, Wolfgang Sipos, Heiko A. Schmidt, Veronika Mlitz, Supawadee Sukseree, Anton Weissenbacher, Erwin Tschachler, Lorenzo Alibardi, Leopold Eckhart

Content

Supplementary Tables S1 – S6

Supplementary Table S1
Tentative abbreviations and full names of EDC genes in *Chrysemys picta*

Species	Gene name abbreviation	Full gene name
Chrysemys picta	Crnn	Cornulin
Chrysemys picta	EDAA1	Epidermal Differentiation protein rich in Aromatic Amino acids 1
Chrysemys picta	EDAA2	Epidermal Differentiation protein rich in Aromatic Amino acids 2
Chrysemys picta	EDAA3	Epidermal Differentiation protein rich in Aromatic Amino acids 3
Chrysemys picta	EDAA4	Epidermal Differentiation protein rich in Aromatic Amino acids 4
Chrysemys picta	EDAA5	Epidermal Differentiation protein rich in Aromatic Amino acids 5
Chrysemys picta	EDAA6	Epidermal Differentiation protein rich in Aromatic Amino acids 6
Chrysemys picta	EDAA7	Epidermal Differentiation protein rich in Aromatic Amino acids 7
Chrysemys picta	EDAA8	Epidermal Differentiation protein rich in Aromatic Amino acids 8
Chrysemys picta	EDAA9	Epidermal Differentiation protein rich in Aromatic Amino acids 9
Chrysemys picta	EDAA10	Epidermal Differentiation protein rich in Aromatic Amino acids 10
Chrysemys picta	EDAA11	Epidermal Differentiation protein rich in Aromatic Amino acids 11
Chrysemys picta	EDAA12	Epidermal Differentiation protein rich in Aromatic Amino acids 12
Chrysemys picta	EDAA13	Epidermal Differentiation protein rich in Aromatic Amino acids 13
Chrysemys picta	EDAA14	Epidermal Differentiation protein rich in Aromatic Amino acids 14
Chrysemys picta	EDAA15	Epidermal Differentiation protein rich in Aromatic Amino acids 15
Chrysemys picta	EDAA16	Epidermal Differentiation protein rich in Aromatic Amino acids 16
Chrysemys picta	EDAA17	Epidermal Differentiation protein rich in Aromatic Amino acids 17
Chrysemys picta	EDAA18	Epidermal Differentiation protein rich in Aromatic Amino acids 18
Chrysemys picta	EDAA21	Epidermal Differentiation protein rich in Aromatic Amino acids 21
Chrysemys picta	EDAA22	Epidermal Differentiation protein rich in Aromatic Amino acids 22
Chrysemys picta	EDbeta1	Epidermal Differentiation protein beta (beta-keratin) 1
Chrysemys picta	EDbeta2	Epidermal Differentiation protein beta (beta-keratin) 2
Chrysemys picta	EDKM	Epidermal Differentiation protein containing a KKLIQQ Motif
Chrysemys picta	EDP1	Epidermal Differentiation protein rich in Proline 1
Chrysemys picta	EDP2	Epidermal Differentiation protein rich in Proline 2
Chrysemys picta	EDP3	Epidermal Differentiation protein rich in Proline 3
Chrysemys picta	EDPCV1	Epidermal Differentiation protein rich in Proline, Cysteine and Valine 1
Chrysemys picta	EDPCV2	Epidermal Differentiation protein rich in Proline, Cysteine and Valine 2
Chrysemys picta	EDPCV3	Epidermal Differentiation protein rich in Proline, Cysteine and Valine 3
Chrysemys picta	EDPCV4	Epidermal Differentiation protein rich in Proline, Cysteine and Valine 4
Chrysemys picta	EDPCV5	Epidermal Differentiation protein rich in Proline, Cysteine and Valine 5
Chrysemys picta	EDPCV6	Epidermal Differentiation protein rich in Proline, Cysteine and Valine 6
Chrysemys picta	EDPCV7	Epidermal Differentiation protein rich in Proline, Cysteine and Valine 7
Chrysemys picta	EDPCV8	Epidermal Differentiation protein rich in Proline, Cysteine and Valine 8
Chrysemys picta	EDPCV9	Epidermal Differentiation protein rich in Proline, Cysteine and Valine 9
Chrysemys picta	EDPCV10	Epidermal Differentiation protein rich in Proline, Cysteine and Valine 10
Chrysemys picta	EDPCV11	Epidermal Differentiation protein rich in Proline, Cysteine and Valine 11
Chrysemys picta	EDPCV12	Epidermal Differentiation protein rich in Proline, Cysteine and Valine 12
Chrysemys picta	EDPCV13	Epidermal Differentiation protein rich in Proline, Cysteine and Valine 13
Chrysemys picta	EDPCV14	Epidermal Differentiation protein rich in Proline, Cysteine and Valine 14
Chrysemys picta	EDPCV15	Epidermal Differentiation protein rich in Proline, Cysteine and Valine 15
Chrysemys picta	EDPE	Epidermal Differentiation protein rich in Proline and glutamic acid (E)
Chrysemys picta	EDPL1	Epidermal Differentiation Proline-rich protein, close to Loricrin, 1
Chrysemys picta	EDPQ1	Epidermal Differentiation protein rich in Proline and glutamine (Q) 1
Chrysemys picta	EDPQ2	Epidermal Differentiation protein rich in Proline and glutamine (Q) 2
Chrysemys picta	EDQL	Epidermal Differentiation protein rich in glutamine (Q), close to Loricrin
Chrysemys picta	EDQM1	Epidermal Differentiation protein containing a glutamine (Q) Motif 1
Chrysemys picta	EDQM2	Epidermal Differentiation protein containing a glutamine (Q) Motif 2
Chrysemys picta	EDQM3	Epidermal Differentiation protein containing a glutamine (Q) Motif 3
Chrysemys picta	EDQM4	Epidermal Differentiation protein containing a glutamine (Q) Motif 4
Chrysemys picta	EDQM5	Epidermal Differentiation protein containing a glutamine (Q) Motif 5
Chrysemys picta	EDQM6	Epidermal Differentiation protein containing a glutamine (Q) Motif 6
Chrysemys picta	EDQM7	Epidermal Differentiation protein containing a glutamine (Q) Motif 7
Chrysemys picta	EDQM8	Epidermal Differentiation protein containing a glutamine (Q) Motif 8
Chrysemys picta	EDWM	Epidermal Differentiation protein containing WYDP Motif
Chrysemys picta	EDYM1	Epidermal Differentiation protein containing Y Motif 1
Chrysemys picta	Lor	Loricrin
Chrysemys picta	Pglyrp3	Peptidoglycan recognition protein 3
Chrysemys picta	Scfn	Scaffoldin

NOTE - Genes of the main beta-keratin gene cluster and S100A genes are not included here.

Suppl. Table S2A

Western painted turtle *Chrysemys picta bellii* EDC and related genes

Gene	Accession nr.	CDS start	CDS end	Expression confirmed by RNA-seq data
S100A1	NW_007281429.1	14272	17371	yes
S100A13	NW_007281429.8	34709	36274	yes
S100A14	NW_007281429.7	38691	41083	yes
S100A4-like	NW_007281429.6	76480	78681	no
S100A2-like 1	NW_007281429.5	83904	84560	no
S100A2-like 2	NW_007281429.5	92858	95973	no
S100A4	NW_007281429.4	99204	100383	yes
S100A5	NW_007281429.3	105793	106688	yes
S100A6	NW_007281429.2	108926	110289	yes
S100A7	NW_007281429.1	130785	131826	yes
S100A12	NW_007281429.0	919813	921972	yes
PGLYRP3	NW_007281429.1	945790	948573	yes
EDKM	NW_007281429.1	956038	956573	yes
EDQM1	NW_007281429.1	966063	965767	no
EDQM2	NW_007281429.1	977351	977064	no
EDQM3	NW_007281429.1	980759	981055	no
EDQM4	NW_007281429.1	985624	985325	no
EDQM5	NW_007281429.1	989554	989850	no
EDQM6	NW_007281429.1	994595	994299	no
EDQM7	NW_007281429.1	997887	998183	no
EDQM8	NW_007281429.1	1002794	1002498	no
EDWM	NW_007281429.1	1011168	1010626	no
EDQL	NW_007281429.1	1018639	1018421	no
LOR-partial	NW_007281429.1	1025940	1024173	no
EDPL1	NW_007281429.1	1049594	1049397	no
EDYM1	NW_007281429.1	1058038	1057526	no
EDAA1	NW_007281429.1	1089591	1089310	no
EDAA2	NW_007281429.1	1096092	1096304	no
EDAA3	NW_007281429.1	1101495	1101277	no
EDAA4	NW_007281429.1	1175917	1176147	no
EDAA5	NW_007281429.1	1183381	1183160	no
EDAA6	NW_007281429.1	1188493	1188705	no
EDAA7	NW_007281429.1	1194163	1193942	no
EDAA8	NW_007281429.1	1203066	1202806	no
EDAA9	NW_007281429.1	1209101	1209331	no
EDAA10	NW_007281429.1	1302661	1302413	no
EDAA11	NW_007307341.1	608	378	n.a.
EDAA12	NW_007306063.1	608	378	n.a.
EDAA13	NW_007295621.1	671	901	n.a.
EDAA14	NW_007286563.1	4325	4095	n.a.
EDAA15	NW_007284381.1	11696	11926	n.a.
EDAA16	NW_007284381.1	5762	5532	n.a.
EDAA17	NW_007284381.1	1172	1393	n.a.
EDAA18	NW_007286375.1	5287	5508	n.a.
EDAA19	NW_007282178.1	8553	8332	no
EDAA20	NW_007282178.1	17818	17597	no
EDAA21	NW_007282178.1	24458	24679	no
EDAA22	NW_007284676.1	2449	2670	n.a.
EDP1	NW_007281429.1	1745592	1745020	no
EDP2	NW_007281429.1	1758947	1758441	no
EDPE	NW_007281429.1	1772376	1773518	no
EDP3	NW_007281429.1	1778600	1778418	yes
EDPQ1	NW_007281429.1	1784025	1784378	yes
EDPQ2	NW_007281429.1	1791904	1792283	yes
EDPCV1	NW_007281429.1	1796867	1796439	no
EDPCV2	NW_007281429.1	1803508	1803780	no
EDPCV3	NW_007281429.1	1813639	1814222	no
EDPCV4	NW_007281429.1	1820502	1820137	no
EDPCV5	NW_007281429.1	1825113	1825448	no
EDPCV6	NW_007281429.1	1832160	1831816	no
EDPCV7	NW_007281429.1	1836997	1837419	yes
EDPCV8-partial	NW_007281429.1	1854445	<1854179	yes
EDPCV9	NW_007281429.1	1858298	1858651	no
EDPCV10	NW_007281429.1	1865591	1865271	yes
EDPCV11	NW_007328050.1	366	704	n.a.
EDPCV12	NW_007301624.1	43	387	n.a.
EDPCV13	NW_007284487.1	1526	1984	n.a.
EDPCV14	NW_007283637.1	1802	2254	n.a.
EDPCV15	NW_007283637.1	11177	11569	n.a.
CRNN	NW_007281429.1	1889578	1892139	no
SCFN-partial	NW_007281429.1	1905413	1919585	yes
S100A11	NW_007281429.1	1926091	1930102	yes

Notes - CDS, coding sequence; n.d., not determined; n.a. not applicable.
The symbols < and > indicate that ends of the coding sequence were not present on the scaffold.
Contigs of EDAA11 and EDAA12 have 98% identical nucleotide sequences and identical beginning.
Beta-keratin genes are not included here.

Suppl. Table S2B

Western painted turtle *Chrysemys picta bellii* beta-keratin genes

Gene	Accession nr.	CDS start	CDS end
EDbeta1	NW_007281429.1	1068086	1067787
EDbeta2	NW_007281429.1	1073627	1073328
Beta-A1	NW_007281429.1	1221404	1221946
Beta-A2	NW_007281429.1	1227812	1227300
Beta-A3	NW_007281429.1	1232913	1232557
Beta-A4	NW_007281429.1	1238857	1239357
Beta-A5	NW_007281429.1	1244817	1244311
Beta-A6	NW_007281429.1	1255598	1255074
Beta-A7	NW_007281429.1	1262738	1263268
Beta-A8	NW_007281429.1	1266045	1265725
Beta-A9	NW_007281429.1	1272115	1272609
Beta-A10	NW_007281429.1	1277733	1277368
Beta-B1	NW_007281429.1	1311841	1311398
Beta-B2	NW_007281429.1	1323678	1323409
Beta-B3	NW_007281429.1	1358869	1359462
Beta-B4	NW_007281429.1	1363692	1363099
Beta-B5	NW_007281429.1	1392765	1392532
Beta-B6	NW_007281429.1	1400809	1400411
Beta-B7	NW_007281429.1	1414967	1414569
Beta-B8	NW_007281429.1	1426768	1426361
Beta-B9	NW_007281429.1	1431942	1432160
Beta-B10	NW_007281429.1	1452827	1452231
Beta-B11	NW_007281429.1	1463460	1462954
Beta-B12	NW_007281429.1	1473077	1473625
Beta-B13	NW_007281429.1	1479472	1479942
Beta-B14	NW_007281429.1	1493090	1493488
Beta-B15	NW_007281429.1	1504652	1505050
Beta-B16	NW_007281429.1	1516699	1517097
Beta-B17	NW_007281429.1	1532013	1532411
Beta-B18	NW_007281429.1	1536315	1535917
Beta-B19	NW_007281429.1	1540929	1541327
Beta-B20	NW_007281429.1	1547570	1547172
Beta-B21	NW_007281429.1	1551278	1551676
Beta-B22	NW_007281429.1	1555985	1555575
Beta-B23	NW_007281429.1	1563465	1563863
Beta-B24	NW_007281429.1	1568853	1568296
Beta-B25	NW_007281429.1	1584967	1584521
Beta-B26	NW_007281429.1	1592788	1593246
Beta-B27	NW_007281429.1	1630944	1630501
Beta-B28	NW_007281429.1	1641756	1641259
Beta-B29	NW_007281429.1	1646851	1646348
Beta-B30	NW_007281429.1	1655316	1655819
Beta-B31	NW_007281429.1	1661046	1660543
Beta-B32	NW_007281429.1	1673002	1672484
Beta-B33	NW_007281429.1	1685593	1685051
Beta-B34	NW_007281429.1	1698616	1698074
Beta-B35	NW_007281429.1	1708560	1707973
Beta-B36	NW_007281429.1	1730188	1729709
Beta-A1L1	NW_007295316.1	298	858
Beta-A1L2	NW_007283747.1	10082	10642
Beta-B17L	NW_007357687.1	90	488
Beta-B18L	NW_007351657.1	996	1394
Beta-O1	NW_007281530.1	1866927	1866487
Beta-O2	NW_007281530.1	1878475	1878915
Beta-O3	NW_007284315.1	7904	8452
Beta-O4	NW_007306927.1	879	439
Beta-O5	NW_007307261.1	919	479
Beta-O6	NW_007284788.1	1495	1127
Beta-O7	NW_007281980.1	49619	49987
Beta-O8	NW_007284421.1	6417	6785
Beta-O9	NW_007299123.1	79	498
Beta-O10	NW_007337571.1	2687	2268
Beta-O11	NW_007282030.1	33208	33627
Beta-O12	NW_007282723.1	2923	3342
Beta-O13	NW_007285688.1	649	1068
Beta-O14	NW_007282723.1	44024	44443
Beta-O15	NW_007284434.1	3624	4061
Beta-O16	NW_007283772.1	13629	13192
Beta-O17	NW_007282173.1	68401	68838
Beta-O18	NW_007282173.1	8154	7732
Beta-O19	NW_007282030.1	80425	80865
Beta-O20	NW_007286070.1	2133	2570
Beta-O21	NW_007285023.1	9662	9225
Beta-O22	NW_007284070.1	9973	9536
Beta-O23	NW_007282723.1	20021	20458
Beta-O24	NW_007283734.1	7236	7673
Beta-O25	NW_007285589.1	4598	4161
Beta-O26	NW_007284118.1	2918	3325
Beta-O27	NW_007282340.1	53526	53119
Beta-O28	NW_007305077.1	418	843
Beta-O29	NW_007286872.1	295	720

Note - CDS, coding sequence.

Suppl. Table S2C

Western painted turtle *Chrysemys picta bellii* partial beta-keratin genes

Gene	Accession nr.	CDS start	CDS end
Beta-p1	NW_007281429.1	1288079	>1288360
Beta-p2	NW_007281429.1	1297097	>1297471
Beta-p3	NW_007281429.1	1610400	>1610771
Beta-p4	NW_007309963.1	717	>998
Beta-p5	NW_007281530.1	1835247	>1834948
Beta-p6	NW_007281530.1	1877180	1877467
Beta-p7	NW_007284266.1	6219	>6746
Beta-p8	NW_007312745.1	602	>928
Beta-p9	NW_007303124.1	779	>1243
Beta-p10	NW_007284082.1	5517	>6053
Beta-p11	NW_007305201.1	<3	521
Beta-p12	NW_007282030.1	14653	>14895
Beta-p13	NW_007282340.1	19970	<19728
Beta-p14	NW_007282340.1	63694	<63356
Beta-p15	NW_007301237.1	>1339	935
Beta-p16	NW_007297142.1	<1	375
Beta-p17	NW_007299554.1	<1	255

Notes - CDS, coding sequence.

The symbols < and > indicate that ends of the coding sequence were not present on the scaffold.

Suppl. Table S3

Green sea turtle *Chelonia mydas* EDC related genes

Gene	Accession nr.	CDS start	CDS end
S100A12	NW_006666501.1	68848	66632
PGLYRP3	NW_006666501.1	49058	46106
EDKM	NW_006666501.1	39190	39044
EDQM1	NW_006666501.1	26343	26564
EDQM2	NW_006666501.1	12945	13202
EDQM3	NW_006666501.1	7329	7072
EDQM4-partial	NW_006581571.1	91560	91589
EDWM	NW_006581571.1	87240	87761
EDQL-partial	NW_006581571.1	72904	73211
LOR-partial	NW_006581571.1	65336	66987
EDPL1mut	NW_006581571.1	40144	40500
EDYM1	NW_006581571.1	30097	30603
EDbeta1	NW_006581571.1	20666	20965
EDbeta2	NW_006581571.1	15641	15940
EDAA1	NW_006618844.1	12655	12425
EDAA2-partial	NW_006605119.1	6741	>6935
EDAA3	NW_006605119.1	11428	11207
EDAA4	NW_006605119.1	20822	20583
EDAA10-like	NW_006579794.1	125068	125316
EDAAO1	NW_006654175.1	672005	672229
EDAAO2-partial	NW_006706580.1	207	>320
Beta-A1-like	NW_006605119.1	40249	40734
Beta-B36-like	NW_006589899.1	22435	21983
EDP1	NW_006589899.1	36953	36543
EDP2	NW_006589899.1	50928	50416
EDP3	NW_006620999.1	6565	6756
EDP3L-partial	NW_006593002.1	<2	184
EDPCV1	NW_006612892.1	2974	2603
EDPCV2	NW_006612892.1	8093	8410
EDPCV3	NW_006612892.1	16189	15863
EDPCV4-fused	NW_006612892.1	24874	24455
EDPCV5	NW_006612892.1	28308	28580
EDPCV6	NW_006612892.1	36193	36510
EDPCV7-partial	NW_006640425.1	475	>624
EDPCV8	NW_006579140.1	724	365
EDPCV9	NW_006647290.1	3758490	3758747
CRNN	NW_006647290.1	3732282	3729834
SCFN	NW_006647290.1	3714784	3705629
S100A11-partial	NW_006647290.1	>3685053	3684187

Notes - CDS, coding sequence.

The symbols < and > indicate that ends of the coding sequence were not present on the scaffold.

Only the first and the last gene of the main beta-keratin cluster are indicated.

Suppl. Table S4A
EDC genes and related genes of *Pelodiscus sinensis*

Gene	Accession nr.	CDS start	CDS end	Expression confirmed by RNA-seq data
S100A12-partial	NW_005853395.1	40971	<40149	yes
PGLYRP3	NW_005853395.1	8585	4835	no
EDKM-partial	NW_005856649.1	1542	>2160	yes
EDQM1	NW_005856649.1	>14845	14759	yes
EDQM2	NW_005856649.1	29466	29140	yes
EDQL	NW_005856649.1	38597	38379	yes
Lor-partial	NW_005856649.1	49344	<49066	yes
EDPL1	NW_005856649.1	75700	75299	yes
EDYM1	NW_005856649.1	87919	87407	yes
EDAA1-partial	NW_005856649.1	<124860	124940	yes
EDAA2-partial	NW_005855424.1	<4987	5067	yes
EDAA3-partial	NW_005855424.1	25646	25443	yes
EDAA4-partial	NW_005855424.1	32648	32852	yes
EDAA5	NW_005855424.1	43933	44142	yes
EDAA6	NW_005855424.1	50778	50557	yes
EDAA7	NW_005857151.1	897	1121	yes
EDAA8	NW_005853100.1	1125816	1125706	no
EDP1A-partial	NW_005854020.1	39149	38495	yes
EDP1B	NW_005854020.1	43373	42675	yes
EDP2	NW_005854020.1	60896	60405	yes
EDP4	NW_005854020.1	87726	86956	yes
EDP5	NW_005854020.1	99701	99126	yes
EDP6	NW_005854020.1	104798	106018	yes
EDPCV1	NW_005856448.1	38543	38836	yes
EDPCV2	NW_005856448.1	10144	10548	yes
EDPCV3	NW_005856448.1	2730	3032	no
EDPCV4	NW_005854374.1	35445	35128	yes
SCFN exon 2	NW_005854801.1	1094	957	yes
SCFN exon 3	NW_005852012.1	115937	112076	yes
S100A11-partial	NW_005852012.1	>91333	91160	yes

Notes - n.a., not applicable; n.d., not determined. For beta-keratin genes, see Suppl. Table S4B.
The symbols < and > indicate that ends of the coding sequence were not present on the scaffold.
SCFN exon 3 was partially re-sequenced and found to lack premature stop codons.
The amino acid sequence of Ps_SCFN (Fig. S2B) differs from the translation of NW_005852012.1.

Suppl. Table S4B
Beta-keratin genes of *Pelodiscus sinensis*

Gene	Accession nr.	CDS start	CDS end
EDbeta1	NW_005856649.1	98695	98399
beta1	NW_005855424.1	63829	64257
beta2	NW_005855424.1	69435	69043
beta3	NW_005855424.1	77992	78390
beta4	NW_005855424.1	85432	85040
beta5	NW_005855424.1	96312	96704
beta6	NW_005855424.1	103633	103241
beta7	NW_005859062.1	6208	5948
beta8	NW_005859062.1	12458	12084
beta9	NW_005859062.1	21403	21789
beta10	NW_005859062.1	28806	28432
beta11	NW_005859062.1	32967	33365
beta12	NW_005859062.1	38336	37962
beta13	NW_005859062.1	44144	44542
beta14	NW_005859062.1	50063	50461
beta15	NW_005859062.1	55226	54819
beta16	NW_005859062.1	98548	98985
beta17	NW_005859062.1	129236	128580
beta18	NW_005859062.1	134400	134996
beta19	NW_005856726.1	57244	56828
beta20	NW_005856726.1	70492	70223
beta21	NW_005856726.1	75358	75089
beta22	NW_005856726.1	109400	108828
beta23	NW_005856726.1	119265	118549
beta24	NW_005856726.1	131020	130457
beta25	NW_005856726.1	153300	151990
beta26	NW_005856726.1	164107	163331
beta27	NW_005856726.1	171808	172395
beta28	NW_005857404.1	2092	2496
beta29	NW_005857404.1	5648	6052
beta30	NW_005857404.1	12458	12066
beta31	NW_005857404.1	18761	19162
beta32	NW_005857404.1	29821	29429
beta33	NW_005857404.1	40813	40298
beta34	NW_005857404.1	48692	49084
beta35	NW_005857404.1	59580	59176
beta36	NW_005857404.1	66269	66673
beta37	NW_005857404.1	76424	76816
beta38	NW_005857404.1	82108	81620
beta39	NW_005857404.1	94016	93492
beta40	NW_005857404.1	99897	100355
beta41	NW_005857404.1	103517	103909
beta42	NW_005857404.1	111063	111455
beta43	NW_005857404.1	122467	123066
beta44	NW_005857404.1	127548	127952
beta45	NW_005857404.1	135528	135010
beta46	NW_005857404.1	142059	142577
beta47	NW_005857404.1	154083	153523
beta48	NW_005857404.1	160552	161061
beta49	NW_005857404.1	171148	171696
beta50	NW_005857404.1	178202	178732
beta51	NW_005851315.1	6976	7437
beta52	NW_005851315.1	14859	15125
beta53	NW_005851315.1	19991	20437
beta54	NW_005851315.1	25942	26388
beta55	NW_005851315.1	29687	30031
beta56	NW_005851315.1	48842	49258
beta57	NW_005851315.1	66396	66842
beta58	NW_005851315.1	88113	88523
beta59	NW_005851315.1	97314	97571
beta60	NW_005851515.1	864	484
beta61	NW_005851515.1	10845	11831
beta62	NW_005851515.1	22566	22147
beta63-partial	NW_005851515.1	29766	>30206
beta64	NW_005851515.1	52402	52905
beta65	NW_005851515.1	72310	72825
beta66	NW_005851515.1	83793	83239
beta67	NW_005851515.1	93560	94084
beta68	NW_005851515.1	108705	109220
beta69	NW_005853269.1	45707	45216
beta70	NW_005853269.1	60041	59550
beta71	NW_005858327.1	3703	3116
beta72-partial	NW_005858327.1	16881	>17297
beta73	NW_005856853.1	11358	10768
beta74	NW_005852318.1	1255	695
beta75-partial	NW_005870254.1	<793	488
beta76	NW_005854020.1	23969	23571

Notes - n.a., not applicable; n.d. not determined.
The symbols < and > indicate that ends of the coding sequence were not present on the scaffold.

Supplementary Table S5. Primers for RT-PCR analysis of *Emys orbicularis*

Target gene(s)	Exon in which primer anneals	Orientation of primer relative to gene	Primer sequence	Scaffold containing the annealing site used for primer design (Accession number)	Annealing site start (Nucleotide number)	Annealing site end (Nucleotide number)	Notes
Beta-A1	exon 1	sense	CTTCATCCCCTCGGTGAACTG	NW_007281429.1	1220330	1220350	
Beta-A1	exon 2	anti-sense	CGGCTCGTTGCAGCTGCCAGA	NW_007281429.1	1221461	1221481	same anti-sense primer as for Beta-A4
Beta-A4	exon 1	sense	CTTCAGCTCCTCAGTCAACTG	NW_007281429.1	1237822	1237842	
Beta-A4	exon 2	anti-sense	CGGCTCGTTGCAGCTGCCAGA	NW_007281429.1	1221461	1221481	same anti-sense primer as for Beta-A1
Beta-p1	exon 1	sense	CTTCATCTCCTCAGTGAACAC	NW_007281429.1	1286991	1287011	gene not amplified in *E. orbicularis*
Beta-O17-like	exon 1	sense	CTTCATCTCCTCAGTGAACAC	NW_007282173.1	67319	67339	annealing with mismatches
Beta-p1	exon 2	anti-sense	AATTGGTCCTGGGATGGTTAC	NW_007281429.1	1288211	1288231	gene not amplified in *E. orbicularis*
Beta-O17-like	exon 2	anti-sense	AATTGGTCCTGGGATGGTTAC	NW_007282173.1	68533	68553	annealing with mismatches
Beta-B8	exon 1	sense	CTTCTTCTCCTCGGTGAACTG	NW_007281429.1	1427463	1427443	gene not amplified in *E. orbicularis*
Beta-B32	exon 1	sense	CTTCTTCTCCTCGGTGAACTG	NW_007281429.1	1673882	1673862	annealing with mismatches
Beta-B8	exon 2	anti-sense	GACAACTGGTGGTGGATAGAC	NW_007281429.1	1426642	1426622	gene not amplified in *E. orbicularis*
Beta-B32	exon 2	anti-sense	GACAACTGGTGGTGGATAGAC	NW_007281429.1	1672894	1672874	annealing with mismatches
Beta-B19	exon 1	sense	GACTTCATCCCCTTGGTGCACT	NW_007281429.1	1540259	1540280	
Beta-B19	exon 2	anti-sense	TGGTAAGGTGCTTCCCACAAT	NW_007281429.1	1541118	1541138	
Beta-B3	exon 1	sense	ACTTAATCTCCTTGGTGAACA	NW_007281429.1	1347417	1347437	
Beta-B3	exon 2	anti-sense	TCCATAGTGACATGGGTTCCA	NW_007281429.1	1359424	1359444	
EDbeta1	exon 1	sense	CGAGTTATTCTCAGTGAACTGG	NW_007281429.1	1068750	1068729	
EDbeta1	exon 2	anti-sense	ACGACAACCGGTGGTGGGTAG	NW_007281429.1	1067961	1067941	
EDAA8	exon 1	sense	ACTTCTTCTGTCTTACTCTCC	NW_007281429.1	1208021	1208041	
EDAA8	exon 2	anti-sense	GGCAAAACATGGCCAGCATCT	NW_007281429.1	1176115	1176135	
EDAA19	exon 1	sense	TCACTTACTCTCCTCGGTGAC	NW_007282178.1	9818	9798	
EDAA19	exon 2	anti-sense	GGTACCAACAACCTTTGGGAT	NW_007282178.1	8423	8403	
EDKM	exon 2	sense	CTGCACCATCACCCCGGAATG	NW_006666501.1	39208	38188	
EDKM	exon 3	anti-sense	CGACCGTGACCACTATCCAAG	NW_006666501.1	37570	37550	
EDP3	exon 1	sense	CCTTGTACCTACAGCTGAAAC	NW_007281429.1	1779587	1779567	
EDP3	exon 2	anti-sense	CTGCTTCCAGTCTTGGGACTG	NW_007281429.1	1778444	1778424	
EDPCV	exon 1	sense	TTTGTTCCTGTTGGTGACTTG	NW_007281429.1	1812055	1812075	
EDPCV	exon 2	anti-sense	CAGTGCTGCACAGGTGGGCAT	NW_007281429.1	1837374	1837394	
EDQM1	exon 1	sense	CACGAGTTCTTCTCTGCATTC	NW_007281429.1	966842	966822	
EDQM1	exon 2	anti-sense	CACCTGGCAGCAGTGCTTCTG	NW_007281429.1	965807	965787	
EDQM7	exon 1	sense	CACGTTCCTGGAGGTGAATAG	NW_007281429.1	997067	997087	
EDQM7	exon 2	anti-sense	TGGCAGGGCACCTTGCAGATC	NW_007281429.1	998150	998170	
EDWM	exon 1	sense	ACGGTCCTTGTTGGTCAATAG	NW_006581571.1	86642	86662	
EDWM	exon 2	anti-sense	ACTGTTGCACATGTTGCTTTG	NW_006581571.1	87673	87693	
LOR	exon 1	sense	ATTTGTTTCCAGTTGCTGAAC	NW_007281429.1	1028005	1027985	
LOR	exon 2	anti-sense	GCAATTATAATCTTCTGGCAG	NW_007281429.1	1025761	1025741	
GAPDH	exon 5	sense	CTTTGGCCAAGGTCATCAAT	NW_006634294.1	527040	527059	
GAPDH	exon 6	anti-sense	CAGAACATCATCCCAGCATC	NW_006634294.1	527493	527474	

Notes - The primers were designed using genome sequences of *C. picta* or *C. mydas* . The primers were used to amplify cDNAs of *E. orbicularis* .
Primer sequences are shown in 5'-3' direction. Annealing site starts and end refer to numbers on the scaffold but not to the 5´ and 3´ ends of the primers.

Suppl. Table S6
Chicken (*Gallus gallus*) beta-keratin genes within the EDC

Gene	Orientation within EDC	Accession number	CDS start	CDS end
GgEDbeta	+	NC_006112.1	825202	825525
GgBet1	-	NC_006112.2	854410	854012
GgBet2	+	NC_006112.2	855666	856082
GgBet3	-	NC_006112.2	858814	858425
GgBet4	+	NC_006112.2	860071	860460
GgBet5	+	NC_006112.2	864665	865081
GgBet6	-	NC_006112.2	867842	867435
GgBet7	+	NC_006112.2	869099	869506
GgBet8	-	NC_006112.2	872116	871700
GgBet9	+	NC_006112.2	873374	873781
GgBet10	-	NC_006112.2	876526	876110
GgBet11	+	NC_006112.2	877786	878175
GgBet12	-	NC_006112.2	880823	880434
GgBet13	+	NC_006112.2	882416	882823
GgBet14	-	NC_006112.2	885937	885545
GgBet15	-	NC_006112.2	895274	894897
GgBet16	+	NC_006112.2	906469	906762
GgBet17	+	NC_006112.2	909282	909578
GgBet18	+	NC_006112.2	912696	912992
GgBet19	+	NC_006112.2	920610	920906
GgBet20	+	NC_006112.2	921450	921746
GgBet21	+	NC_006112.2	927670	927966
GgBet22	+	NC_006112.2	929118	929414
GgBet23	+	NC_006112.2	932678	932974
GgBet24	+	NC_006112.2	936060	936356
GgBet25	+	NC_006112.2	939315	939611
GgBet26	+	NC_006112.2	944034	944330
GgBet27	+	NC_006112.2	947082	947378
GgBet28	+	NC_006112.2	950614	950910
GgBet29	+	NC_006112.2	956699	957049
GgBet30	+	NC_006112.2	960981	961331
GgBet31	+	NC_006112.2	967208	967558
GgBet32	+	NC_006112.2	973298	973642
GgBet33	-	NC_006112.2	975768	975106
GgBet34	+	NC_006112.2	979013	979657
GgBet35	-	NC_006112.2	981718	981359
GgBet36	+	NC_006112.2	982872	983237
GgBet37	-	NC_006112.2	985238	984873
GgBet38	-	NC_006112.2	989144	988779
GgBet39	+	NC_006112.2	990297	990662
GgBet40	-	NC_006112.2	993140	992775
GgBet41	+	NC_006112.2	994299	994664
GgBet42	-	NC_006112.2	997105	996740
GgBet43	+	NC_006112.2	998247	998612
GgBet44	-	NC_006112.2	1001042	1000677
GgBet45	+	NC_006112.2	1002152	1002517
GgBet46	+	NC_006112.2	1005687	1006052
GgBet47	-	NC_006112.2	1008543	1008178
GgBet48	+	NC_006112.2	1009693	1010058
GgBet49	-	NC_006112.2	1013391	1012921
GgBet50	+	NC_006112.2	1017136	1017624
GgBet51	-	NC_006112.2	1020045	1019563
GgBet52	+	NC_006112.2	1023690	1024181
GgBet53	+	NC_006112.2	1029671	1030066
GgBet54	-	NC_006112.2	1034534	1034160
GgBet55	+	NC_006112.2	1038872	1039195
GgBet56	-	NC_006112.2	1042806	1042375
GgBet57	-	NC_006112.2	1045505	1045008
GgBet58	-	NC_006112.2	1049893	1049459
GgBet59	-	NC_006112.2	1057634	1057089
GgBet60	-	NC_006112.2	1063389	1062721
GgBet61	+	NC_006112.2	1066589	1067158
GgBet62	+	NC_006112.2	1070763	1071293
GgBet63	-	NC_006112.2	1074197	1073667
GgBet64	-	NC_006112.2	1081224	1080733

Note - CDS, coding sequence.
For phylogenetic analyses, sequences of "feather" beta-keratins encoded by genes outside of the EDC were taken from Ng et al. 2014.
