# Supplementary material for: Comparative Genomics Identifies Epidermal Proteins Associated with the Evolution of the Turtle Shell
Source: Mol Biol Evol. 2015 Nov 24;33(3):726–37. doi: 10.1093/molbev/msv265 (PMC4760078; doi:10.1093/molbev/msv265)
Supplement: Supplementary Data [file supp_msv265_suppl_data.zip › Supplementary_Figures_Holthaus-et-al.pdf]

Supplementary Data: Supplementary Figures

Comparative genomics identifies epidermal differentiation proteins associated with the evolution of the turtle shell

Karin Brigit Holthaus, Bettina Strasser, Wolfgang Sipos, Heiko A. Schmidt, Veronika Mlitz, Supawadee Sukseree, Anton Weissenbacher, Erwin Tschachler, Lorenzo Alibardi, Leopold Eckhart

Content

Supplementary Figures S1 – S21

A

>Cp_CRNN
MTQLLSNIKGIINAFYVFAKKDGACPTLSKGELRQLIHQEFADVTVVPQGLQTIDKLLQLLDTDSDGRLDFNGFLVLIFQVAK
ACYGEVSQGQRPGHGGSSASQGEANCGERTKEPPTPERDPSPRQAPEPQTPERDSIPCQAPEPQIPEQDSSPCQAPELQIPEQ
DSNPCQAPEAQIPERDPSPCQAPELQTPEQDSSPRQAPEPQIPEQDSSPRQALEPQIPEQDSIPCQAPEAQIPERDPSPHQAP
EPQTPERDPSPHQGLEPQTPERDPSPCHAPEPQIPEQDPSLHQAPEPQTPEQDSSSRHTPEPQIPEQEPSPCRGPEPQTPEQD
LSPSQAPEPQTPEQDLSHSETQLPPTQQRNQGAQDPTEPAAGQASKSSQCLYSWHSQKPRPFPHWWPPKK

>Cp_EDAA1
MFHHQKICKPWCCKPHQKICKPWCCKPWGYGGSSGYGGDYGYCPPFWCKKPFKCCYPYPYPGCYPYPKPCCYPCPYPYPCGPG
YQYPCLAEEE

>Cp_EDAA2
MSFNKSIIGELYYNPCCYGGYRGYRGYGYCRPWCYQRPYKYGWGHHYKCCYPYPYQWGYGKGWPCFAEEE

>Cp_EDAA3
MNYHHQKLSHHWGCDPCWNGGWGGYGGHYGCYRPWGYYRPYGWGWGHSYGYPYRWGGGYGYGRCWPCFAEEE

>Cp_EDAA4
MTYHHQKLSHHWGCDPCWNGGWGGYGGHYGCYRPWGYYRPYSYGWGHNYGSCYSYPYRWGGGYGYGRCWPCFAEEQ

>Cp_EDAA5
MTFDELMNEELYYNPYCYKGWRGYRGHYGCYRPWGYQRPYRYGWGHQYDCHYPYRWGHGYGYGKFWPCFAEEQ

>Cp_EDAA6
MTFDESINDELYYNPWSHGCWHGSRGHYGCGRPWGYGRQSRWGWGHGYDCYYPYSSRWGHWYPYVKQWPC

>Cp_EDAA7
MTFDELMNEELYYNPYCYKGWRGYRGHYGCYRPWGYQRPYRYGWGHQYDCHYPYRWGHGYGYGKFWPCFAEEQ

>Cp_EDAA8
MTFHHQKLSHHWGCDPCSSGSWGGYRGHYDCYRPWGYSRPYGCGWGYNDGCYYPYSSRWGHGYGGYGYGGCGYGYGGHGYGKC
WPC

>Cp_EDAA9
MNYHHQKLSHHWGCDPCWNGGWGGYGGHYGCYRPWGYYRPYSYGWGHNSGSCYSYPYRWGGGYGYGRCWPCFAEEQ

>Cp_EDAA10
MTWSGYGYNDGCYSPCGYGGRWAYGSPCGYRGLCGYGGHSSHGGSWGYRGSYGYRGAYHSGYCYPFSSQQGHRYSYGNCGPC

>Cp_EDAA11
MTYHHQKLSHHWGCDPCWNGGWGGYGGHYGCYRPWGYYRPYSYGWGHNSGSCYSYPYRWGGGYGYGRCWPCFAEEQ

>Cp_EDAA12
MNYHHQKLSHHWGCDPCWNGGWGGYGGYYGCYRPWGYYRPYSYGWGHNSGSCYSYPYRWGGGYGYGRCWPCFAEEQ

>Cp_EDAA13
MTYHHQKLSHHWGCDPCWNGGWGGYGGHYGCYRPWGYYRPYSYGWGHNYGSCYSYPYRWGGGYGYGRCWPCFAEEQ

>Cp_EDAA14
MTYHHQKLSHHWGCDPCWNGGWGGYGGHYGCYRPWGYYRPYSYGWGHNYGSCYSYPYRWGGGYGYGRCWPCFAEEQ

>Cp_EDAA15
MNYHHQKLSHHWGCDPCWNGGWGGYGGHYGCYRPWGYYRPYSYGWGHNSGSCYSYPYRWGGGYGYGRCWPCFAEEE

>Cp_EDAA16
MNYHHQKLSHHWGCDPCWNGGWGGYGGHYGCYRPWGYYRPYSYGWGHNYGSCYSYPYRWGGGYGYGRCWPCFAEEE

>Cp_EDAA17
MTFDELMNEELYYNPYCYKGWRGYRGHYGCYRPWGYQRPYRYGWGHQYDCHYPYRWGHGYGYGKFWPCFAEEQ

>Cp_EDAA18
MTFDELMNEELYYNPYCYKGWRGYRGHYGCYRPWGYQRPYRYGWGHQYDCHYPYRWGHGYGYGKFWPCFAEEQ

>Cp_EDAA19
MTFDENFSDELYYKPYHYGGWGGRGYGYCRPWCYQRPYKCCWGYPKGCWYPDPCHWGWGYGYGKGWPCFAQEE

>Cp_EDAA20
MTFDENFSEKLDYKPCHYGGWRGRGYGWGRPWCYQRPYRCCWGYPKGCWYPYPCHWGWGYGYGKGWPCFAQEE

>Cp_EDAA21
MTFDENFSDELYYKPYHYGGWGGRGYGYCKPWCHQRPYKCCWGYPKGCWYPYPCHWGWGYGYGKDWPCFAQEE

>Cp_EDAA22
MTFDENFSDELYYKPYHYGGWGGRGYGWCRPWCYQRPYKCCWGYPKGCWYPYPCHWGWGYGYGKGWPCFAQEE

>Cp_EDKM
MSNLIKAIADMIDSYQSNSRKGRESERFRRCEFKKLVQQDPTPAKRSSSNKHKHTTSLPDSDAELMNKKELITANPCVQ

>Cp_EDP1
MPYYGQQHKHLPAPVCVTKCSQPCPPQYEQHCVPKCRPVYVTKCPPLYGPQYAYPCAPQCPPRCVTKCPPRCVTKCPPPCVTK
CPPPCVTKCPPPCVTKCPPPCVTKCPPPCVTKCPPPCVTKCPPPCVTKCPPPCVTKCPPPCVTKCPPPCMTKCPQQCVTQCPG
QYQSGKVQISSHCKKYCSAPKWPW

>Cp_EDP2
MASRQNQQQRKQTLTLPPALSNATSEPAPPPEAVPEPCPATVEEPENSPQEEEGPQEEYKQPLNQPLGPAPELEPEPVLCPEP
ESNPPEVKEIEYLQPDHQQYKHPPTLPPAPGMETSKEYQQAESEPELGRCPPPIREPEGPPFVQPSSPVEEQQQKQPHHWPPK
RK

>Cp_EDP3
MSSDQQQCKQTCPPPPKCQEKCPPPCKEPVKTPKCQEKCPPPSKEPKCPPPKQSQDWKQC

>Cp_EDPCV1
MAYQQQCKQPCLPPPCCVKQCKTKCVDPCPCPPQCVDPCPPCPPKCVDPCPPKCVDPCPPKCVDLCPPKCVDQFPCPPKCVDV
CPPKCVDLCPPKCVDQCPCPPKCVDVCPPKCVDVCPPKCVDVCPPCPPLQHCCQEKKHY

>Cp_EDPCV2
MAYQQQCKQTCLPPPCCVTKCVTKCLDPCYKVCVTKCVTKCLDPCCKVCVKKCTRCVHPCSCPPKHVDPCPPCLPKCPPVQHC
CKEKKPC

>Cp_EDPCV3
MAYQQQCKQTCLPPPCCVTKCTTKCLDPCCKVCVTKCVTKCVDPCCKVCVKKCTTCVHPCPCPCPQKCLPCPPCPQKCPPCPP
CPPCPQKCPPCPQKCPPCPPCPPCPQKCPPCPPCPQKCPPCPPCPPKCSPVQHCCKEKKLC

>Cp_EDPCV4
MAYQQQCKQTCLPPPCCVTKCTTKCLDPCCKVCVTKCVTKCVDPCCKVCVKKCTTCVHPCPCPCPQKCIPCPQKCIPCPPCPQ
KCPPCPPCPQKCPPCPPCPPCLPKCPPVQHCCKEKKLC

>Cp_EDPCV5
MAYQQQCKQTCLPPPCCVTKCTTKCLDPCCKVCVTKCVTKCVDPCCKVCVKKCTTCVHPCPCPCPQKCLPCPPCPQKCPPCPP
CPQKCPPCPPCLPKCPPVQHCCKEKKLC

>Cp_EDPCV6
MAYQQQCKQTCLPPPCCVTKCTTKCLDPCCKVCVTKCVTKCVDPCCKVCVKKCTTCVHPCPCPCPQKCLPCPPCPPCHQKCPP
CPQKCPPCPPCPPCPPKCPPVQHCCKEKKLC

>Cp_EDPCV7
MAYQQQCKQTCLPPPCCVTKCTTKCLDPCCKVCVTKCVTKCVDPCCKVCVKKCTKCVHPCPCPCPQKCIPCPPCPQKCPPCPP
CPPCPQKCPPCPPCPPCPQKCPPCLPCPQKCPPCPPCPPCLPKCPPVQHCCKEKKLC

>Cp_EDPCV8_partial
MAYQQQCKQTCLPPPCCVTKCTTKCLDPCCKVCVTKCVTKCVDPCCKVCVKKCTTCVHPCPCPCPQKCLPCPPCPQKCPPCPP
CPPCPQXXXXXXXXXX

>Cp_EDPCV9
MAYQQQCKQTCLPPPCCVTKCTTKCLDPCCKVCVTKCVTKCVDPCCKVCVKKCTTCVHPCPCPCPQKCPPCAPCPPCPQKCPP
CPPCPPCHQKCPPCPPCLPKCPPVQHCCKEKKLC

>Cp_EDPCV10
MAYQQQCKQPCLPPPCCVTKCTTKCLDPCCKVCVKKCTTCVHPCPCPCPCPQKCLPCPPCPPCPQKCPPCPPCPPCPEKCPPC
PPCPPCPPECPPVQHCCKEKKLC

>Cp_EDPCV11
MAYQQQCKQTCLPPPCCVTKCTTKCLDPCCKVCVTKCVTKCVDPCCKVCVKKCTTCVHPCPCPQKCIPCPPCPQKCPPCPPCP
QKCPPCPPCPPCLPKCPPVQHCCKEKKLC

>Cp_EDPCV12
MAYQQQCKQTCLPPPCCVTKCTTKCLDPCCKVCVTKCVTKCVDPCCNVCVKKCTTCVHPCPCPCPQKCIPCPPCPQKCPPCPP
CPQKCPPCPPCPPCLPKCPPVQHCCKEKKLC

>Cp_EDPCV13
MAYQQQCKQTCLPPPCCVTKCTTKCLDPCCKVCVTKCVTKCVDPCCKVCVKKCTTCVHPCPCPCPQKCIPCPPCPQKCPPCPQ
KCLPCPQKCIPCPPCPQKCPPCPQKCLPCPQKCLPCPPCPQKCPPCPPCPPCPQKCPPCPPCPPCPQKCPPCPPCPPCLPCPQ
KCPPCPPKCPPVQHCCKEKKLC

>Cp_EDPCV14
MAYQQQCKQTCLPPPCCVTKCTTKCLDPCCKVCVTKCVTKCVDPCCKVCVKKCTTCVHPCPCPCPQKCLPCPQKCLPCPPCPQ
KCPPCPPCPPCPQKCPPCLPCPPCPQKCPPCPPCPPCLPCPQKCPPCPPCPPKCPPVQHCCKEKKLC

>Cp_EDPCV15
MAYQQQCKQTCLPPPCCVTKCTTKCLDPCCKVCVTKCVTKCVDPCCKVCVKKCTTCVHPCPCPCPQKCLPCPPCPQKCPPCPP
CPPCPQKCPPCPPCPPCLPCPQKCPPCPPCPPKCPPVQHCCKEKKLC

>Cp_EDPE
MSLHQDQQQCKQGITLPPALCKEKCPEMVPCPEPVKCPEPIPCCPVKPPCKEPPVPIPTPCPEPIPCPQQKQQCKLPPVPVPL
PHPEPIPCSPEKPPCKEPPFPHPLPHPEPIPHCPEKPPCKEPKVPVPLPHPEPIPYCPDKSPCKQPPAPVTPPCPKPIPCSPE
KPPCKEPPFPHPLPHPEPIPCPQHKQQCKLPPVPVPLPHPEPIPHCPEKPPCKEPKVPVPLPHPELIPHCPEKPPCKEPPFPH
PLPHPEPIPHCPEKPPCKEPKVPVPLPHPEPIPCPQQKQQCKLPPVPVPTPCPEPIPCSPEKPPCKEPPAPVTPPCPKPRPCP
ENPPCKEPPVPVTPPCPEPVKCPPCVKTCPPIEQQQCKQQCQLPPNWK

>Cp_EDPL1
MSCHQHQQQCKQPCMPPPCKEPDRPKTTEPCPQQCTEPCPQQCTEPRPPKCVETCPPKCPPVQQC

>Cp_EDPQ1
MSYQHQQQCKQTCLPPPVCPPPQCPEPCPPPKCPEPCPPPKCPELCPPPQCPEPCPPLKCPEPCPPPKCQEPCPPPKCPEPCP
PPKCPEPCPPPQCQEPCRPPKCPSPKCPPMQKYN

>Cp_EDPQ2
MSYQHQQQCKQTCLPPPVCPPPQCPEPCPPPKCPEPCPPPKCPEPCPAPQCPEPCPPPKCPEPCPAPQCPEPCPPLKCPEPCP
PPKCPEPCPQPQCQEPCPPPQCQEPCKPPKCPSPKCPPMQKYN

>Cp_EDQM1
MCSRQEKDHCHKQDTCHGSGGGSSCHGSGGGSSCHGSGGGSSCHGSGGGSSCHGSGGGSSCHGSGGGSSCHGKPQKPCQQEQQ
QQQKHCCQVPSQKLK

>Cp_EDQM2
MCSRQEKDHCHKQDVSGGCHSSGSSCHSSGGSSCHSGGSSCHGSGGSSCHSSGGSSCHGGGSSCHSGGSCHGKPQQPCQQQQQ
QKICKVPCQKLK

>Cp_EDQM3
MCSRQEKDHCHKQDGCHSSGGCHSSGSSCHSGGGSSCHSGGSSCHGSGGSSCHSSGGSSCHGGGSSCHSGGSCHGKPQQHCQQ
QQQQKICKVPCQKLK

>Cp_EDQM4
MCSRQEKDHCHKQDGCHSSGGCHSSGSSCHSGGGSSCHSGGSSCHGSGGSSCHSSGGSSCHGGGSSCHSGGSCHGKPQQHCQQ
QQQQQKICKVPCQKLK

>Cp_EDQM5
MCSRQEKDHCHKQDGCHSSGGCHSSGSSCHSSGGSSCHSGGSSCHGSGGSSCHSSGGSSCHGGGSSCHSGGSCHGKPQQHCQQ
QQQQKICKVPCQKRK

>Cp_EDQM6
MCSRQEKDHCHKQDGCHSSGGCHSSRSSCHESGGSSCHSGGSSCHGSGGSSCHSSGGSSCHGGGSSCHSGGSCHGKPQQHCQQ
QQQQKICKVPCQKLK

>Cp_EDQM7
MCSRQEKDHCHKQDGCHSSGGCHSSGSSCHSSGGSSCHSGGSSCHGSGGSSCHSSGGSSCHGGGSSCHSGGSCHGKPQQHCQQ
QQQQKICKVPCQKLK

>Cp_EDQM8
MCSRQEKDHCHKQDGCHSSGGCHSSGSSCHSSGGSSCHSGGSSCHGSGGSSCHSSGGSSCHGGGSSCHSGGSCHGKPQQHCQQ
QQQQKICKVPCQKLK

>Cp_EDQL
MCSREPRGCHDSGSSSCHDSGSSTCHSSGGGSCHDVKPLPQCPTPVPCQTTTLPCQQQTKQPCQWPPQKHQK

>Cp_EDWM
MIYSSGRESYFNLNSTWYDPAGSWLDTRRTPFTYAYSTCCSSGGCPRGGHDNRCYEYRRSGCGENCHGSSGSCHGSGGHCCVR
RPSYFHGYSGGCHGHGRSVCSERSCHGSGSSCHGSGSSCHGSGSSCHGSGSSCHNTSGACHSTPIYVKPKQYVQQCCPPVQQC
CLPVKKCCPPVQKC

>Cp_EDYM1
MSYFAYQYKQRNYTPYSTTRLIPHAEPCVVKGPAPRVTKCADPCAVKHPAPCTTKCRDPCAGKPSVPCATKCFEPHAQRHPAK
HYPKFSEPAGVKCSTPCDTRYHEPYGLIHPQPFPERWNPCAPPYVHPYVTGYPQACGPTYVPSFPKYPYPYAPQWPNTWGYGN
CGPC

>Cp_LOR_partial
MCSHQEKKACHEIPTQAGGCHAGGGGSSGSGSGALLGPHILGSSSYGVGGGSSYCGSGESCQKIIIAGGSSGSSSGSSGGSYG
CGVGGGSGGGSGGSYGCGVGGGSGGGSGGSGQKIIIAGGESGGSYGCGVGGGSGGGSGGSGQKIIIAGGESGGSYXXXXXXXX
XXYGCGVGGGSGGGSGGSGQKIIIAGGGSGGSYGCGVGGGSGGGSGGSSGGCTGGGSGYSSGGSAGYCSGGSDDFGSGGSLQS
MQQKCPIVIPCMEQQQTKQPCQWPPNPRK

>Cp_PGLYRP3
MFRLAVFLCALCAVSWGFLCLRIVSPGKWGGRPANCSSPLKAVQTGYVIVLHTAGGSCKTGAECNQQMTNIQHYHMNNKGWCN
IAYNFLIGEDGKVYEGRGWNTEGAHTYGYNDISLGIAFMGDFTGRSPNAAAWIALKHLLHFAVENGYLSSDYLLMAHGDVSHT
ISPGQPIRKVLKTWPHYKH

>Cp_SCFN_partial
MPQLLDSISTLISVFYKPGKKDEDCSTISKREVKRFIQRGFADITVNPYDAHAIEAVLQLLDHDGDGVEDFNEFLLLVFRVAK
VCYWYLQPKQRLPQRTETGLSGERRQEPEAGRAEGSRDQPKPLGTEKGYYETWEPETRETERSRRQPREPEPRGDERANYETY
EPEKREEERRRRQPHGPEPRRDERSWYGTQRSEHRENERSHHRSCEPEPRGDERSRYERRDLETREVDRSRRHPHERERREDE
RSSHQPYEPEPQSRERRCLEPESRQDEKSGYWPRESERSRYETCEPEAREEERSHRQPREPEPRRDERSRSRPHEPQPQGDER
SHYRAHAPETREEERSRCPQQQPEPRRDERSRYEIYDHDTREEERSRCHPHEPEHEEEERSRHQPREPEHEEEERSADSHVSL
NXXXXXXXXXXSRRQPRKPESREDEGSRRQPREPEPREEERSRRQPREPEPREDEGSRRQPRKPEPREDEGSRRQPREPEPRE
DERRRRQPHEPEPREDNRSRRQPHGPELRGDGWSRHQSCEPEAREGERSRCEPCEPEQREEKRSHQQPREPQWREDERSRRQP
REPEPREDEGAADSHVSLNXXXXXXXXXXDEGSRRQPREPEPREDEGSRRQPREPEPREGERCSIQSQTPEPPHEEGNLPPSS
EPVENEGTLREPHDPESTDDSRRCPATHEPPPTGDEGSQLQPRESAAPGDEGNPQKTSESEPREDDGSQPQLCEPEQKEGDGV
LRQTPESQPLEEVESQEQPRGPEPRTEGSRHQPPQGGEASHLQPDIEPQQGDGSRHRPRDPQEERERSSHQAREPLPRLGEEP
EEGEWSQSHPEPANAEEEPGETDPDDVKASLPCNPLYVYLLEQTVEKQLYLAPPHQDHP

B

>Cp_EDbeta1
MSCGANLCIDGGSACGVARPRPCADSCNQPCVTQCPDSRVIIYPPPVVVTFPGPILTTFPQESVVESVGAPVVASGYGGTSGS
GAFGVGHGNCGPCGPC

>Cp_EDbeta2
MSCSRNVCTAGGSACGVARPRPFTDSCNQPCVTRCPDSRVIIYPPPVVVTFPGPILTTFPQESVVESVGAPVVASGYGGTSGS
GAFGVGHGNRDLCGPC

>Cp_Beta-A1
MSCSSLCYPECGVARPSPVSGSCNEPCVRQCPDSEVIIRPSPVVVTIPGPILSNFPQQSEVGAVGAPVVGAGYGGSFGLGGLY
GYGGHYGGLYGYGGLGGYGGRYGYGGLSGYGGLCGYGGRYGYGGLCGYGGRYGYGGLSGYGGLCGYGGGYGGGYGYGGACGSG
VSCHRYLSGSCTPC

>Cp_Beta-A2
MSCSSLCYPECGVARPSPVSGSCNEPCVRQCPDSEVIIRPSPVVVTIPGPILSNFPQQSEVGAVGAPVVGAGYGGSFGLGGLY
GYGGHYGGLYGYGGLGGYGGRYGYGGGYGGLCGYGGRYGYGGLSGYGGRYGGLCGYGGGYGGGYGYGGACGSGVSCHRYLSGS
CTPC

>Cp_Beta-A3
MSCSSLCYPECGVARTSPDSGSCNELCVRQCPDSEVIIRPSPVVVTIPGPILSNFPYRGHYGRLYCYGGLGGYGGHYGYGGLC
GYRGRYGYGGLCGYRGRYGYGGLSGYGGHYGGLCD

>Cp_Beta-A4
MSCSSLCYPECGVARPSPVSGSCNEPCVRQCPDSEVIIRPSPVVVTIPGPILSNFPQQSEVGAVGAPVVGAGYGGSFGLGGLY
GYGGHYGGLYGYGGLGGYGGRYGYGGGYGGLCGYGGRYGYGGLSGYGGLCGYGGGYGGGYGYGGACGSGVSCHRYLSGSCTPC

>Cp_Beta-A5
MSCSSLCYPECGVARPSPVSGSCNEPCVRQCPDSEVIIRPSPVVVTIPGPILSNFPQQSEVGAVGAPVVGAGYGGSFGLGGLY
GYGGHYGGLYGYGGLGGYGGHYGYGGLSGYGGLCGYGGRYGYGGLSGYGGRYGGLCGYGGGYGYGGACGSGVSCHRYLSGSCT
PC

>Cp_Beta-A6
MSCSSLCYPECGVARPSPVSGSCNEPCVRQCPDSEVIIRPSPVVVTIPGPILSNFPQQSEVGAVGAPVVGAGYGGSFGLGGLY
GYGGHYGGLYGYGGLGGYGGRYGYGGGYGGGYGGLCGYGGRYGYGGLSGYGGRYGGLCGYGGGYGGGYGYGGACGSGVSCHRY
LSGSCTPC

>Cp_Beta-A7
MSCSSLCYPECGVARPSPVSGSCNEPCVRQCPDSEVIIRPSPVVVTIPGPILSNFPQQSEVGAVGAPVVGAGYGGSFGLGGLY
GYGGHYGGLYGYGGLGGYGGHYGYGGLSGYGGGYGGLCGYGGRYGYGGLSGYGGRYGGLCGYGGGYGGGYGYGGACGSGVSCH
RYLSGSCTPC

>Cp_Beta-A8
MSCSSRCYPECGVARPSPVSGSCNELCVRQCPDSEVIIRPSPVVVTIPGQILSNFPYGGHYGRLYGYGGLGGYGGHYGYGGLC
GYGGRYGYGGLSGYGGHYGGLCD

>Cp_Beta-A9
MSCSSLCYPECGVARPSPVSGSCNEPCVRQCPDSEVIIRPSPVVVTIPGPILSNFPQQSEVGAVGAPVVGAGYGGSFGFGGLN
GYGGHYGGLYGYGRGYGGLSGYGGGYGGLCGYGGHYGYGGLSGYGGRYGGLCGYGGGYGYGGACGSGVSCHRYLSGSCTPC

>Cp_Beta-A10
MSCSSMCYPECGVTRPSPVSGSCNEPCVRQCPDSEVIIRPSPVVVTIPGPILSNFPQQSRVGAIGAPVVGPGYGASFSLGGLY
GSGSCYGGLYSYGILYGYGGLGGYRGWLWGIRSLWGIM

>Cp_Beta-A1L1
MSCSSLSYPECGVARPSPVSGTCNEPCIRQCPDSEVVIRPSPVVVTLPGPILSTFPQQSEVAAVGAPVVGAGYGGSFGLGGLY
GSGGRYGGLYGLGGFGGYGGLYGYGGLGGYGGLCGYGGGYGYGGLGGYGGLGRYGGLCGYGGGYGGLCGYGGGYGYGGLGGYG
GLCGYGGYGRRYRGGYCGPC

>Cp_Beta-A1L2
MSCSSLSYPECGVARPSPVSGSCNEPCVRQCPDSEVVIRPSPVVVTLPGPILSTFPQQSGVGAVGAPVVGAGYGGSFGLGGLY
GSGGHYGGLYGLGGLGGYGSHYGYGGLGGYGGLCGYGGGYGYGGLGGYGGLGRYGGLCGYGGGYGGLCGYGGGYGYGGLGGYG
GLCGYGGYGRRYRGGYCGPC

>Cp_Beta-B1
MSFNGPQTGAQGSLPCGVKCSEPYIATASEPCVVKCKDSRVIIYPPPVVVTFPGPILTTCPQESIVASSGPPDTGVAESAARI
SAAPRVTGSLGPHLDRCPASINIRHEAQYTPKYSYTYSSRWSHPGKSLETTGYSQTTRNIDRTK

>Cp_Beta-B2
MSCYGLRNIPCEVPRPTPAAVTYNEPCVIQCPDSIFESDSPPGIAIIPGPILTTFPHYSVVETSPLFDTERSFCSERSLGSQG
FMNLYN

>Cp_Beta-B3
MFSDEEFFYKNKQPQKPQKGQNPCLPQKKPKPCPPQKVPCTPKPPRCPTYPPYIPPPRPCPPQYPSICPQPYIGVWNEPCVTE
CGDSTAVVFAPPVVVNFPGPTLATCPQDSVVGSSLPRGIIGPYGPGGSLSSGGAFGTGSSFGSSVSSFSSAGSFGSGGYGGSG
GYLGSGGFLGSGGYCGSGGWNPCHYGRCGPC

>Cp_Beta-B4
MFSDEEFWYKSQQPQKPQKGQNPCLPQKKPKPCPPQKVPCTPKPPRCPTYPPYIPPPRPCPPQYPSIWPQPYIGVWNEPCVTE
CGDSTAVVFAPPVVVNFPGPTLATCPQDSVVGSSLPRGIIGPYGSGGSLGSGGSFGAGSSFGSSVSSFSSGGSFGSGGYGGSR
GFLGSGGYGGSGGYCGFGGWNPCHYGRCGPC

>Cp_Beta-B5
MSSYEQLCNTQCYAPCNVTCPQPIVDTCNEPCITSCSDSRAVVYPPLIVVTFPGTLLSFCPQESVEESSAHVGIRSS

>Cp_Beta-B6
MSSYRQLCNTQCYAPCNVTCPRPFVDACNEPCFTSCGDSSAVLYPPPVIVRFPGPILATCPQESVVGSSAPFGIGSSLGIGGP
YVSGSLGNYGGSYTSGLSARGNGCSYPSSSSQRFTTYRSGSCQPYQTQK

>Cp_Beta-B7
MSSYRQLCNTQCYAPCNVTCPRPFVDACNEPCFTSCGDSSAVLYPPPVIVRFPGPILATCPQESVVGSSAPFGIGSSLGIGGP
YVSGSLGNYGGSYTSGLSARGNGCSYPSSSSQRFTTYRSGSCQPYETQK

>Cp_Beta-B8
MSFCRDLCKYPSYPSCDVTCPQPFVDACNQPCVTSCGDSSVVVYPPPVVVRFPGPILATCPQESVVGSSEPLGIGSSFGYRGS
YLSGSSYGYKSLYNDRRSYTPGLSSLGRGSSDPCSSRWLNMYGCGPRQTQQE
>Cp_Beta-B9
MSFYGDPARSQCYLPCEGTCQQPVANVCNEPWVRSGGDSRGVGYAPLVVVTFPGPSSQYLLSGKHDWNGTAK

>Cp_Beta-B10
MSSHRQLVSPRCATPWEVTCPQPGANICSQPCVTSCEDSRVMVYAPPVVVAFPGPILSTCPQKSITGSEVPGEMGAYLDLEGH
MVLGAHMVSGLPMALGKHMVMKSHLVLGDHMVQTTHGSGGSYGHGGSYSASNSYGARGPYGAGRFLVFRGSYGSGGSYSHSRA
YTSRLSPLGTGNSCPYSSQRTSMSHYKNCGPY

>Cp_Beta-B11
MSSCKDLSCRPSPCYPDICPDPCVVARNEPCITSCADSTAVVYPPPVSVLFPGPILSSSPQHSLVGSTLPALPYGARGSFGGG
ALGGPIGYGSGYGGALEGGYGYGGLSGYGGSYGYGGLSGYGGSYGYGGLCGYGGGYGGGYGGLCGYGRRYGGRCYSSRRGSCG
PC

>Cp_Beta-B12
MSSCKDLSCRPSPCYPDICPDPCVVARNEPCITSCADSTAVVYPPPVSVLFPGPILSSSPQHSVVGSTLPALPYRAGGSFGGG
ALGGPIGYGSGYGGALEGGYGYGGLSSYGGSYGYGGLSGYGGSYGYGGLCGYGAGYGGGYGGLCGYGAGYGGGYGGLCGYGRR
YGGRCYSSRRGSCGPC

>Cp_Beta-B13
MSSCKDLSCRPSPCYPDICPDPCVVARNEPCITSCADSTAVVYPPPVSVLFPGPILSSCPQHSLVGSTLPALPYRAGGSFGGG
ALGGPIGYGSGYGGALEGGYGYGGLSSYGGSYGYGGLCGYGGGYGGGYGGLCGYGRRYGGRCYSSRRGSCGPC

>Cp_Beta-B14
MSSSKDLCYPRPPCYPDICPNPYVDAWNEPCVTSCGDSSAVVYAPPVVVRFPGPILATCPQDSVVGSTLPNLPYGYGSPYGGG
SFGGSVSSGGAYGGGYGAGYGGGYGGLYGYGKGYGRKCYSSRFGSCGPC

>Cp_Beta-B15
MSSSKALCYPRPPCYPDICPDPYVDACNEPCVTSCGDSSAVVYAPPVVVRFPGPILATCPQDSVVGSTLPNLPYGYGGSYGGG
SFSVSVGSGGAYGGGYGARYGGGYGGLYGYGKGYGRKCYSSRFGSCGPC

>Cp_Beta-B16
MSSSKALCYPRPPCYPDICPDPYVDACNEPCVTSCGDSSAVVYAPPVVVRFPGPILATCPQDSVVGSTLPNLPYGYGGSYGGG
SFSGSVGSGGAYGGGYGARYGGGYGGLYGYGKGYGRKCYSSRFGSCGPC

>Cp_Beta-B17
MSSSKALCYPRPPCYPDICPDPYVDACNEPCVTSCGDSSAVVYAPPVVVRFPGPILATCPQDSVVGSTLPNLPYGYGGSYGGG
SFSGSVGSGGAYGGGYGAGYGGGYGGLYGYGKGYGRKCYSSRFGSCGPC

>Cp_Beta-B18
MSSSKDLCYPRPSCYPDICPNPYVDAWNEPCVTSCGDSSAVVYAPPVVVRFPGPILSTCPQDSVVGSTLPNLPYGYGGSYGAG
SFGGSVISGGAYGGRYGAGYGGGYGGLYGYGKGYGRKCYSSRFGSCGPC

>Cp_Beta-B19
MSSSKALCYPRPPCYPDICPDPYVDAWNEPCVTSCGDSSAVVYAPPVVVRFPGPILATCPQDSIVGSTLPNLPYGYGGSYGGG
SFGGSVGSRGAYGGGYGAGYGGGYGGLYGYGKGYGRKCYSSRFGSCGPC

>Cp_Beta-B20
MSSSKALCYPRPPCYPDICPNPYVDACNEPCVTSCGDSSAVVYAPPVVVRFPGPILATCPQDSVVGSTLPNLPYGYRGSYESG
SFGGSVGSRGAYGGGYGAGYGGGYGGLYGYGKGYGRKCYSSRFGSCGPC

>Cp_Beta-B21
MSSSKALCYPRPPCYPDICPDPYVDAWNEPCVTSCGDSSAVVYAPPVVVRFPGPILATCPQDSVVGSTLPNLPYGYGGPYGGG
SFSGSVGSGVAYGGGYGAGYGGGYGGLYGYGKGYGRKCYSSRFGSCGPC

>Cp_Beta-B22
MSSSKALCYPRPPCYPDICPDPYVDAWNEPCVTSCGDSSAVVYAPPVVVRFPGPILATCPQDSVVGSTLPNLPYGYRGSYGGG
SFGGSVGSSGVYGGVYGGVYGGGYDGGYGGLCGYGRRYGRKSYSSRFGSCGPC

>Cp_Beta-B23
MSSSKALCYPRLPCHPDICPNPYVDAWNESCVTSCGDSSAVVYAPPVVVRFPGPILATCPQDSVVGSTLPNLPYRYEGPYGGG
SFGGSGGSGRAYGGRYNVGYGSRYGDLCGHGRRFGRKCYSSRFESCRPC

>Cp_Beta-B24
MFAEFLCWQPRPYCPDICPDPCAYVCNKPFVTSCGDSNGVVYAPPDVMRFPGQALTTCPQDSFVGTVGPRLFYSSWGGLGGHA
GGGFGGGSWGWYGGGSGGGFRGGYGGSFRGGYGGRYGSGYGGGYGGGYGGGFGSGSGGRFGGGLGGSYGYGGSYGGNSYANRW

>Cp_Beta-B25
MSPVKDLCCQPGPYYPDICPDPCAYVCNEPCVTTCGDSNAVVFAPPVVVRFPGPTLATCPQDSFVGTSLPNFPYRLGGGLGGR
IGGGLGGGYGGGYGGGYGGGSFGGFGGGFGGGFGGGIGGGYRGSYGYGGRYGRNCYANRWECCPW

>Cp_Beta-B26
MSPVKDLCYQPRPYCPDICPEPCAYVCNEPCVTTCGDSNAVVFAPPVVVRFPGPTLATCPQDSFVGTSLPNFPYRLGGGLGGR
IGGGLGGGYGGGYGGGYGVGSFGGFGGGSGGGFGGGFGGGIGGGYRGSYGYGGRYGRNCYANRWECCPW

>Cp_Beta-B27
MSPVKDLCCQPRPYCPDICPDPCAYVCNEPCVTSCGDSSAVVFAPPVVVRFPGPTLATCPQDSFVGTSLPNFPYTPWGGLGGR
AGAGLGGGSWSGYGGGFGVGAGGGFGGGFGGGFGGGFGAGYGGSYGNWGRYGRRCSYTSYRCPC

>Cp_Beta-B28
MSCCPPQDCIPDICPCPYIDVRNEPCISSCGDSTAVVYAPPVVVNFPGPTMATCPQDSFIGTSLPNMPVRAGASYGSGGGFSG
SIGSRGSYGAGFGGGYGGGFRGGYGGGHGGGYGGGFGGGLRCGYGGSYGYGGPCGYGRRSQRGITVLGGGYSGSSYGNCGPC

>Cp_Beta-B29
MSYCPPQDCIPDICPRPYIDVCNEPCISSCGDSNAVVFAPPVVVRFPGPTMATCPQDSIVGSSLPNMPIRAGGLYGSGGGFSG
SITSGGSYGGGFGGGYSGGYGGGSSIVYGGGAGGGYGGGAGGGYGAGYGGSYGCGGSRGYIRKSYRSISGGGYSGFNRGNCGP
C

>Cp_Beta-B30
MSYCPPQDCIPDICPRPYIDVCNEPCISSCGDSNAVVFAPPVVVRFPGPTMATCPQDSIVGSSLPNMPIRAGGSYGSGGGFSG
SITSGGSYGGGFGGGYSGGYGGGSSIVYGGGAGGGYGGGAGGGYGAGYGGSYGCGGSRGYIRKSYRSISGGGYSGFNRGNCGP
C

>Cp_Beta-B31
MSYCPPQDCIPDICPRPYIDVCNEPCISSCGDSTAVVFAPPVVVRFPGPTMATCPQDSFVGSSLPNMPIRAGASYGSGGGFSG
SITSGGSYGGGLGGGYSGGYGGGSSIVYGGGAGGGYGGGAGGGYGAGYGGSYGCGGSRGYIRKSYRSISGGGYSGFNRGNCGP
C

>Cp_Beta-B32
MSYCPPQDCYPDICPRPCIDVRNEPCISSCGDSTAVVYAPPVVVRFPGPTMATCPQDSFVGSSLPNLPIRPGGSYGGSISYGG
GYGGGYGGGNSVVGSGGGFGGSTGYGGVYGGGAGSGYGGGYGGGAGGGYGGCYGGSYGSGGSRGYSKKSYRSISGGGYSGVNR
GNCVQS

>Cp_Beta-B33
MSYCPPQDCYPDICPRPCIDVRNEPCISSCGDSTAVVYAPPVVVRFPGPTMATCPQDSFVGSSLPNLPIRPGGSYGGSLTYAG
GYGGGYGGGNSVVGSGGGFGGSTGYGGVYGGGVGGGYGGGYGGGAGGGYGGGAGGGYGGCYGGSYGSGGSRGYSKKSYRSICG
GGYSGVNRGNCGPC

>Cp_Beta-B34
MTYCPPQDCYPDICPRPCIDVRNEPCISSCGDSTAVVFAPPVVVRFPGPTMATCPQDSFVGSSLPNLPIRPGGSYGGSITYGG
GYGGGYGGGNSVVGSGGGFGGSTGYGGVYGGGVGGGYGGGYGGGAGGGYGGGAGGGYGGCYGGSYGSGGSYGYNKKSYRSISG
GGYCGVKSGNYGPC

>Cp_Beta-B35
MSYCPPQDCYPDICPRPCIDVRNEPCISSCGDSTAVVFAPPVVVRFPGPTMATCPQDSFVGTSLPNLPIRPVGSNGGSISYGG
GYGGGYGGGNSVVGSGGGFGGSTGYGGVYGGGVGGGYGGVYGGGVGGGYGGGYGAGVGCGYGGGAAGGYGGCYGGSYGSGGSR
GYSKKSYRSISGGRSSGVKSGNPEKPSME

>Cp_Beta-B36
MSFNGVPCNDQCHNPCEVTCPQPIVNSSNQPCVVSCGDSRVVIYPPPVVVTFPGPILSTCPQDSIVGSSAASGSRISGSASVI
SSTPGVTGCSKPYAESVFVRSEPQYTPKYSYTYSSPWIHPGNTSGSGHYRSSYVQKACPRNEEPQQNEKQDAEQCS

>Cp_Beta-B17L
MSSSKALCYPRPPCYPDICPDPYVDACNEPCVTSCGDSSAVVYAPPVVVRFPGPILATCPQDSVVGSTLPNLPYGYGGSYGGG
SFSGSVGSGGAYGGGYGARYGGGYGGLYGYGKGYGRKCYSSRFGSCGPC

>Cp_Beta-B18L
MSSSKALCYPRPPCYPDICPNPYVDAWNEPCVTSCGDSSAVVYAPPVVVRFPGPILATCPQDSVVGSTLPNLPYGYRGSYGSG
SFGGSVSSGGAYAGGYGAGYGGGYGGLYGYGKGYGRKCYSSRFGSCGPC

>Cp_Beta-O1
MTFSSLCYPECGVARPSPVTGSSNEPCVRQCPDSEVVIRPSPVVVTLPGPILSNFPQQSEVAAVGAPVVGAGFGGSFGLGGLY
GYGGHYGGLYGLGRLGGYGGRYGYGGLLGYGGHCGYPGLYGYGGLWGYGGYGRRYLSGYCGPC

>Cp_Beta-O2
MTFSSLCYPECGVARPSPVTGTCNEPCVRQCQDSEVVIRPSPVVVTLPGPILSNFPQQSEVAAVGAPVVGAGFGGSFGLGGLY
GYGGHYGGLYGLGRLGGYGGHYGYGGLLGHGGYCGYPGLYGYGGLWGYGGYGRRYLGGYCGPC

>Cp_Beta-O3
MTFSSLCYPECGVARPSPVTGSANEPCVRQCPDSEVVIRPSPVVVTLPGPILSNFPQQSEVAAVGAPVVGAGFGGSYGLGGLY
GYGGHYGGLYGLGRLGGYGGRYGYGGLLGNGGYCGYPGLYGYGRLWGNGGHCGYPGLYGYGGLWGYGGHCGYPGLYGYGGLSG
SGVSNHRYLSGSSGPC

>Cp_Beta-O4
MTFSSLCYPECGVARPSPITGSSNEPCVRQCPDSEVVIRPSPVVVTLPGPILSNFPQESEVAAVGAPVVGAGFGGSYGLGGLY

GYGGHYGGLYGLGRLGGYGGHYGYGGLLGYGGHCGYPGLYGYGGLWGYGGYGRRYLGGYCGPC

>Cp_Beta-O5
MTFSSLCYPECGVARPSPVTGSSNEPCVRQCPDSEVVIRPSPVVVTLPGPILSNFPQESEVAAVGAPVVGAGFGGSFGLGGLY
GYGGHYGGLYGLGRLGGYRGLYGYGRLLGHGGYCGYPGLYGYGGLWGYGGYGRRYLGGYCGPC

>Cp_Beta-O6
MTFSSLCYPECGVARPSPVTGSCNEPCVRQCQDSQVVIRPSPVVVTLPGPIMSNFPQHSAVGAVGAPVVGAGFGGSYGLGGLN
GSGGQYGGLSGLGGYGGYGGLCGSGVSCHRYLSGSGGLC

>Cp_Beta-O7
MTFSSLCYPECGVARPSPVTGSCNEPCVRQCQDSQVVIRPSPVVVTLPGPIMSNFPQHSAVGTVGAPVVGAGFGGSYGLGGLN
GSGGQYGGLSGLGGYGGYGGLCGSGVSCHRYLSGSCGPC

>Cp_Beta-O8
MTFSSLCYPECGVAQPSPVTGSCNEPCVRQCPDSQVVIRPSPVVMTLPGPILSNFPQHSVVGAVGAPVVGAGFGGSYGLGGLN
GSGGHYGGWSGLGGYGGYGGLSGSGVSYHRYLSGSCGPC

>Cp_Beta-O9
MTFSSLCYPECGVARPSPVTGTCNEPCVRQCQDSEVVIRPSPVVVTLPGPIMSNFPQHSGVGAIGAPVVGPGFGGSFGHGGFG
YGGLYGGLHGLGGYGGYGGHYGYGGLGGYLGGYGYGGLCGSGVSCHRYLSGNCGPC

>Cp_Beta-O10
MTFSSLCYPECGVARPSPVTGTCNEPCVRQCQDSEVVIRPSPVVVTLPGPIMSNFPQHSGVGAVGAPVVGPGFGGSFGHGGFG
YGGLYGGLHGLGGYGGYGGHYGYAGLGGYLGGYGYGGLCGSGVSCHRYLSGNCGPC

>Cp_Beta-O11
MTFSSLCYPECGVARPSPVTGTCNEPCVRQCQDSEVVIRPSPVVVTLPGPIMSNFPQHSGVGAVGAPVVGPGFGGSFGHGGFG
YGGLYGGLYGLGGYGGYGGHYGYGGLGGYLGGYGYGGLCGSGVSCHRYLSGNCGPC

>Cp_Beta-O12
MTFSSLCYPECGVARPSPVTGTCNEPCVRQCQDSEVVIRPSPVVVTLPGPIMSNFPQHSGVGAVGAPVVGPGFGGSFGHGGLG
YGGLYGGLYGLGGYGGYGGHYGYGGLGGYLGGYGYGGLCGSGVSCHRYLSGNCGPC

>Cp_Beta-O13
MTFSSLCYPECGVARPSPVTGTCNEPCVRQCQDSEVVIRPSPVVVTLPGPIMSNFPQHSGVGAVGAPVVGPGFGGSFGHGGFG
YGGLYGGLYGLGGYGGYGGHYGYAGLGGYLGGYGYGGLCGSGLSCHRYLSGNCGPC

>Cp_Beta-O14
MTFSSLCYPECGVARPSPVTGTCNEPCVRQCQDSEVVIRPSPVVVTLPGPIMSNFPQHSGVGAVGAPVVGPGFGGSFGRGGFG
YGGLYGGLYGLGGYGGYGGHYGYGGLGGYLGGYGYGGLCGSGLSCHRYLSGNCGPC

>Cp_Beta-O15
MTFSSLCYPECGVARPSPVTGTCNEPCVRQCQDSEVVIRPSPVVVTLPGPIMSNFPQHSGVGAVGAPVVGPGFGGSFGHGGFG
YGGLYGGLYGLGGYGGYGGHYGYGGLGGYGGLGGYLGGYGYGGLCGSGVSCHRYLSGNCGPC

>Cp_Beta-O16
MTFSSLCYPECGVARPSPVTGTCNEPCVRQCQDSEVVIRPSPVVVTLPGPIMSNFPQHSGVGAVGAPVVGPGFGGSFGHGGFG
YGGLYGGLYGLGGYGGYGGHYGYGGLGGYGGLGGYLGGYGYGGLCGSGVSCHRYLSGNCGPC

>Cp_Beta-O17
MTFSSLCYPECGVARPCPVTGTCNEPCVRQCQDSEVVIRPSPVVVTLPGPILSNFPQHSGVGALGAPVVGPGFGGSFGHG
GFGYGGLYGGLYGLGGYGGYGGRYGYGGLWGHGGYCGYPGLYGYGGLLGYGGYGRRYLGGRCGPC

>Cp_Beta-O18
MTFSSLCYPECGVARPCPVTGTCNEPCVRQCQDSEVVIRPSPVVVTLPGPILSNFPQHSAVGALGAPVVGPGFGGSFGHGALY
GYGGLYGGWCGLGGYGGYCGPYGYGGLGGYLGCYGYGGICGSGVSCHRYLSGSCGPC

>Cp_Beta-O19
MTFSSLCYPECGVAQPCPVTGTCNEPCVRQCQDSEVVIRPSPVVVTLPGPIMSHYTQESVVGALGAPVVGPGFGGSFGHGGLY
GYGGRYSGWYGLGGYGGYCGPYGYGSLGGYGGLGGYVGGYGYGGICGSGVSCHRYLSGSCGTC

>Cp_Beta-O20
MTFSSLCYPECGVARPCPVTGTCNEPCVRQCPDSEVVIRPSPVVVTLPGPILSNFPQHSAVGAVGAPVVGPGFGGSFGHGGYG
YGGLYGGLYGLGGYGGYGGHYGYGGLWGHGGYCGYPGLYGYGGLWGYGGYGRRYLGGHCGAW

>Cp_Beta-O21
MTFSSLCYPECGVARPCPVTGTCNEPCVRQCPDSEVVIRPSPVVVTLPGPIMSNFPQHSGVGAVGAPVVGPGFGGSFGHGGYG
YGGLYGGLYGLGGYGGYGGHYGYGGLLGHGGYCGYPGLYGYGGLWGYGGYGRRYLGGRCGTW

>Cp_Beta-O22
MTFSSLCYPECGVARPCPVTGTCNEPCVRQCQDSEVVIRPSPVVVTLPGPILSNFPQHSAVGAVGAPVVGPGFGGSFGHGGYG
YGGLYGGLYGLGGYGGYGGHYGYGGLWGHGGYCGYPGLYGYGGLWGYGGYGRRYLGERCGTC

>Cp_Beta-O23
MTFSSLCYPECGVARPCPVTGTCNEPCVRQCQDSEVVIRPSPVVVTLPGPIMSNFPQHSGVGAVGAPVVGPGFGGSFGHGGFG
YGGLYGGLYGLGGYGGYGGHYGYGGLWGHGGYCGYPGLYGYGGLWGYGGYGRRYLGGRCGTC

>Cp_Beta-O24
MTFSSLCYPECGVARPCPVTGTCNEPCVRQCQDSEVVIRPSPVVVTLPGPIMSNFPQHSGVGAVGAPVVGPGFGGSFGHGGFG
YGGLYGGLYGLGGYGGYGGHYGYGGLWGHGGYCGYPGLYGYGGLWGYGGYGRRYLGGRCGTC

>Cp_Beta-O25
MTFSSLCYPECGVARPCPVTGTCNEPCVRQCQDSEVVIRPSPVVVTLPGPIMSNFPQHSGVGAVGAPVVGPGFGGSFGHGGFG
YGGLYGGLYGLGGYGGYGGHCGYGGLWGHGGYCGYPGLYGYGGLWGYGGYGRRYLGGRCGTC

>Cp_Beta-O26
MTFSSLCYPECGVARPCPVTGTCNEPCVRQCPDSEVVIRPSPVVVTLPGPIMSNFPQHSGVGAVGAPVVGPGFGGSFGHGGFG
YGGLYGGLYGLGGYGGYGGHYGYGGIIGPWGILRLPGSLWLRGIMGIWGIWP

>Cp_Beta-O27
MTFSSLCYPECGVARPCPVTGTCNEPCVRQCQDSEVVIRPSPVVVTLPGPIMSNFPQHSGVGAVGAPVVGPGFGGSFGHGGYG
YGGLYGGLYGLGGYGGYGGHYGYGGLWGHGGYRGYPGLYGYGGVMGIWGIWP

>Cp_Beta-O28
MISSSLCYPECGVARPCPVTGTCNEPCVRQCQDSEVVIRPSPVVVTLPGPILSNFPQHSAVGALGAPVVGPGFGGSFGHGGYG
YGGLYGGLYGLGGYGGYGGRYGYGGLYGFGGLGGYGYGGLCGSRLSCHRYLSGNCGPC

>Cp_Beta-O29
MISSSLCYPECGVARPCPVTGTCNEPCVRQCQDSEVVIRPSPVVVTLPGPILSNFPQHSAVGAVGAPVVGPGFGGSFGHGGFG
YGGLYGGLYGLGGYGGYGGRYGYGGLYGFGGLGGYGYGGLCGSRLSCHRYLSGNCGPC

C

>Cp_Beta-p1
MSCSSLCYPECGVARPSPVSGTCNEPCVRQCPDSEVVIRPSPVVVTIPGPILSNFPQQSEVGAVGAPVVGAGYGGSFGLGGLN
GYGGHYGGLYGXXXXXXXXXX

>Cp_Beta-p2
MSCSSLCYPECGVARPSPVSGSCNEPCVRQCPDSEVVIRPSPVVVTLPGPILSTFPQQSEVAAVGAPVVGAGYGGSFGLGGLY
GSGGYYGGLYGLGGFGGYGSHYGYGGLGGYGGLGGYGGGYGGXXXXXXXXXX

>Cp_Beta-p3
MSPVKDLCCQPGPYCPDICPDPCAYVCNEPCVRTCGDSSAVVFAPPVVVRFPGPTLATCPQDSFVGTSLPNFPYTPWGGLGGR
AGAGLGGGSWSGYGGGFGVGARGGLGGGFGAGFGGGFGGGFXXXXXXXXXX

>Cp_Beta-p4
MSCSSLSYPECGVARPSPVSGSCNEPCVRQCPDSEVVIRPSPVVVTIPGPILSNFPQQSEVGAVGAPLVGAGYGGSFGLGGLN
GYGGHYGGLYGXXXXXXXXXX

>Cp_Beta-p5
MTFSSLCYPECGVARPSPVTGSFNEPCVRQCPDSEVVIRPSPVVVTLPGPILSNFPQESEVAAVGAPVVGAGFGGSFGLGGLY
GYGGHYGGVDGLGGFSRXXXXXXXXXXX

>Cp_Beta-p6
MTFSSLCYPECGVARPSPVTGSANEPCVRQCQDSEVVIRPSPVVVTLPGPILSNFPQQSEVAAVGAPVVGAGFGGSFGLGDCT
AMXXXXXXXXXXCWAFFI

>Cp_Beta-p7
MSCSSLCYPECGVARPSPVSGTCNEPCVRQCPDSEVVIRPSPVVVTIPGPILSNFPQQSEVGAVGAPVVGAGYGGSFGLGGLN
GYGGHYGGLYGLGGFGGYGGLYGYGGLGGYGGLCGYGGGYGYGGLGRYGGLCGYGGGYGGLCGYGGGYGYGGLGGYGGLCGYG
GGYGYGGLGRXXXXXXXXXXX

>Cp_Beta-p8
MSCSSLSYPECGVARPSPVSGSCNEPCIRQCPDSEVVIRPSPVVVTLPGPILSTFPQQSEVAAVGAPVVGAGYGGSFGLGGLY
GSGGHYGGLYGLGGFGGYGGLYGYGGXXXXXXXXXX

>Cp_Beta-p9
MSCSSLSYPECGVARPSPVSGSCNEPCVRQCPDSEVVIRPSPVAVTLPGPILSTFPQQSEVAAVGAPVVGAGYGGSFGLGGLY
GSGGHYGGLYGLGGFGGYGGLYGYGGLGGYGGLCGYGGGYGYGGLGGYGGLGRYGGLCGYGGGYGGLCGYGGXXXXXXXXXX

>Cp_Beta-p10
MSCSSLSYPECGVARPSPVSGSCNEPCVRQCPDSEVVIRPSPVAVTLPGPILSTFPQQSEVAAVGAPVVGAGYGGSFGLGGLY
GSGGQYGGLYGLGGFGGYGGLYGYGGLGRYGGLCGYGGGYGYGGLGGYGGLGRYGGLCGYRGGYGYGGLGGYGGLGRYGGLCG
YGGGYGYGGLGGYXXXXXXXXXXX

>Cp_Beta-p11
XXXXXXXXXXCGVARPSPVSGSCNEPCVRQCPDSEVVIRPSPVVVTLPGPILSNFPQQSEVAAVGAPVVGAGFGGSYGLGGLY
GYGGHYGGLYGLGGYGGYGGRYGYGGLLGNGGYYGYPGLYGYGGLWGNGGHCGYPGLYGYGRLWGYGGHCGYPGLYGYGGLSG
SGVSCHRYLSGSRGPC

>Cp_Beta-p12
MTFSSLCYPECGVARPCPVTGTCNEPCVRQCQDSEVVIRPSPVVVTLPGPIMSNFPQHSGVGAVGAPVVGPGFGGSFGHGGXX
XXXXXXXX

>Cp_Beta-p13
MTFSSLCYPECGVARPCPVTGTCNEPCVRQCPDSEVVIRPSPVVVTLPGPILSNFPQHSGVGALGAPVVGPGFGGSFGHGGXX
XXXXXXXX

>Cp_Beta-p14
MTFSSLCYPECGVARPSPVTGTCNEPCVRQCQDSEVVIRPSPVVVTLPGPIMSNFPQHSGVGAVGPPVVGPGFGGSFGHGGFG
YGGLYGGLHGLGGYGGYGGHYGYGGLGGYLXXXXXXXXXX

>Cp_Beta-p15
XXXXXXXXXXVARPSPVTGSSNEPCVGQCPDSEVVIRPSPVVVTLPGPILSNFPQQSEVAAVGAPVVGAGFGGSFGLGGLYGY
GGHYGGLYGLGRLGGYRGLYGYGGLLGHGGYCGYPGLYGYGGLWGYGGYGRRYLGGYCGPR

>Cp_Beta-p16
XXXXXXXXXXACNEPCVRQCQDSEVVIRPSPVVVTLPGPILSNFPQHSAVGALGAPVVGPGFGGSFGHGGFGYGGLYGGLYGL
GGYGGYGGHYGYGGLWGHGGYCGYPGLYGYGGLWGYGGYGRRYLGGRCGTC

>Cp_Beta-p17
XXXXXXXXXXNPSPVVMTLPGPILSNFPQHSVVGAVGAPVVGAGFGGSYGLGGLNGSGGHYGGLSGLGGYGGYGGLCGSGVTC
HRYLSGSCGPC

Supplementary Figure S1. Amino acid sequences of proteins encoded by EDC genes of *C. picta bellii* (Cp). (A) Amino acid sequences of EDC proteins other than beta-proteins. (B) Amino acid sequences of beta-keratins of completely sequenced genes. (C) Amino acid sequences of beta-keratins of partially sequenced genes. Amino acid residues K and Q (potential transglutamination sites), C (potential disulfide bonding sites), P, G and S are highlighted by specific colors corresponding to those in Figure 3. XXXXXXXXXX, missing amino acid residues (number unknown).

A

>Cm_CRNN
MTQLLSNIKGIINAFYVSAKKDGACPTLSKGELRQLICQEFADVTVVPPGLQTIDKMLQLLDTDSDGRLDFNGFLVLVFQVAK
ACYREVSQGQQPGHGERSASPGEAKCGEHTQEPQTPERDPTPRQAPEFQISERDPSPHQAPEPQTPEHDPSPHQALKPQTPER
DPSPHQVLEPQKPEQDLSPRQALEPPTPERDLSPRQALEPQMSEWDPSPRQAPEPQKPEQNPSSHQAPEPQTPKQDSSPRQTP
EPQTPERDLSPRQAPEFQISEQDPSPHQAPKPQTPEQDPSPRQALEPQMSEWDPSPRQAPEPQKPEQDPSSHQAPEPQTPKQD
SSPCQAPEPQTPEQDPSPQQVREPQTQEQDPSLRQAPEPQTPEQDSSPRQASEPQIPEQNPSPHQDLEPQTPEQDLNHSETEL
PPTQQRNQGVQDPREPAAGQASKSSQCLYSWHYQKPLPFPHWWPPKK

>Cm_EDAA1
MTYHHQKISHHWGCDPRWNGGWGGYRGHYDCYRPWGYYRPYSYGWCPNYDSCYSYPYRWGSGYGYGRCWPCFAEEQ

>Cm_EDAA2_partial
MTFDESINEELFYNPWSHGCWHGSRGHYGCGRPWGYGRQSRLGWGHGYDCYYPYSSRWGHWYPYGXXXXXXXXXX

>Cm_EDAA3
MTFDELMNDELYYNPYCYQGWRGYRGHYGCYRPWGSWKPYRYGWGHQYGGHYPYRWGHGYGYGKFWPCLAEEQ

>Cm_EDAA4
MTYHHQKISHHWGCDPCSYGSWGGYRGHYDCYRPWSYSRPYGYGCSYNDGCYYPYSSRWGHGYGGYGYGSHGYGKCWQE

>Cm_EDAA10L
MTWSGYGCNDGCYSPCGYGGQWACGSPCGYRGLCGYGGRSGHGGSCGYRGSYGYRGSYDSGHCYPFAFQRGHRYSYGNCGPC

>Cm_EDAAO1
MTFYENFSDELYYKPYYYGGWGGSRGYGYCRPWCYRRPYKCCWGYPKGCWHPYPCHWGWGHGYGKGWPCFAEEE

>Cm_EDAAO2_partial
MTFDENFSDELYYKPYYSGARGGSRCNGYHKPCCYQRPXXXXXXXXXX

>Cm_EDbeta1
MSCGGNLCIDGGSACGVARPKPYTDSCNQPCVTQCPDSRVVIYPPPVVVTFPGPILTTFPQESVVESVGAPVVASGYGGTSGS
GAFGVGHGNCGPCGPC

>Cm_EDbeta2
MSCSRNVCTAGGSACGVARPRPFADSCNQPCVTQCPDSRVVIYPPPVVVTFPGPILTTFPQESVVESVGAPVVASGYGGTSDS
GAFGVGRGNRDLCGPC

>Cm_EDKM
MSALIKAIADMIDSYQRNAKKGCESERIRRCEFKKLLQQEPSPAKISSSNKYEHTTSLPDSDAELMIKKELITANPCVY

>Cm_EDP1
MPYYGQQHKQLCLPPPACVTKCSQPYPPQYEQQCVPKCRPVYVTKCPPWYGPQYAYPCAPQCPPPCVTKCPPPCPPPCVTKCP
PPCPPPCVTKCPPPCVTKCPQHCVTQYPDQYQSGKVQISSHGKKYCSGPKWPW

>Cm_EDP2
MASQQNQQQRKQTLTLPLALSNATSEPAPTPEAGPEPCPATVEERENSPQEEEESQEEYKRPLNQPLGPAPELEPEPVLGPEP
ESNPSEVKEIEYLQLDQQQYKHPPTLPPAPGIETSKEYQQAEPELEPEPGRCPPPISEAEGPLFVQPSSPVEEQQQKQPHHWP
PKRK

>Cm_EDP3
MSADQQQCKQTCPPPPKCQEKCPPPCKEPCKPSKCQEQCPPPCKDPCPPKCPPPQQSQDWKHC

>Cm_EDP3L_partial
XXXXXXXXXXDQQQCKQTCPPPPKCQEKCPPPCKEPCKPSKCQEQCPPPCKDPCPPKCPPPQQSQDWKHC

>Cm_EDPCV1
MVYQQQCKQTCLPPPCCVTKCTTKCLDPCCKVCVTKCVKKCVDPCCNVCVKKCTTKCLDPCCKVCVTKCVKKCVDPCCKVCVK
KCTTCVHPCPCPCPQKCPPCPPCFPKCPPVEHCCKEKKFW

>Cm_EDPCV2
MVYQQQCKQPCLPPPCCVTKCTTKCLDPCCKVCVTKCVTKCVDPCCKVCVKKCTTCVHPCPCPCPEKCIPCPEKCIPCPQKCP
PCPPCPEKCPPVQHCCKEKKLC

>Cm_EDPCV3
MVYQQQCKQTCLPPPCCVTKCTTKCLDPCCKVCVTKCVTKCVDPCCKVCVKKCTSCVHPCPCPCPQKCIPCPQKCIPCPQKCI
PCPPCPPCLPKCPPVQHCCKEKKFW

```
>Cm_EDPCV4
MAYQQQCKQPCLPPPCCVTQCTTKCLDPCCKVCVTKCVTKCMDPCCKVCVKKCTTCVHPCPCPPKHVDPCPLCFPKCPPVQHC
CKEKKLC

>Cm_EDPCV5
MSYQHQCKQPCLPPPCCVKQCKTKCVDPCPCPPKCVDPCPPKCMDPCPPKCVDPCPLKCVDQWPCPPKCVDLCPPKCVDPCPP
KCVDPCPPCPPLQHCCKEKKFC

>Cm_EDPCV6_partial
MVYQLQCKQTCLPPPCCVTKCTTKCLDPCCKVCVTKCVKKCVDPCCKVCVXXXXXXXXXX

>Cm_EDPCV7
MVYQQQCKQPCLPPPCCVTKCTTKCLDPCCKVCVTKCVTKCVDPCCKVCVKKCTTCVHPCPCPCPEKCIPFPEKCIPCPEKCI
PCPQKCIPCPEKCIPCPPCPEKCPPVQHYCKEKKIC

>Cm_EDPCV8
MVYQQQCKQTCLPPPCCVTKCTTKCLDPCCKVCETKCVTKCVDPCCKVCVKKCTTCVHPCPCPCPQKCPPKCPPVQHCCKEKK
FW

>Cm_EDPL1-like
MSCQQPQQQCKQPCMPPPCKEPGHPHTTDPGPQQCTEPRAQLCPEPCPPKCVETCSPKCLSSAQSHVPSCAQSHVLQNAWRHV
PQNAHLYSSARNSSVTNKIHSSLARKKPIPNAGSQVM

>Cm_EDQL_partial
MCSREPHGCHDTGSSSCHDTGSSSSPDTGSSFCXXXXXXXXXXSSSCHDTGSSSCHGSGGGTCHDVKPLPQCPIPVPCQTTTI
PCQQQTKQPCQWPPQKHQK

>Cm_EDQM1
MCSRQEKDQCHKQDHCHKQDHCHKEDHCHGSGGGSSCHGSGGSSCHGKPQKPCQQEQQQQQKHCCPVPSQKLK

>Cm_EDQM2
MCSRQEKDHCHKQDHCHSSGSSCHGSGGSSCHSGGSSCHSSGGSSCHGGGSSCHSGGSSCHGKPQQHCQQQQQQKICKVPCQK
LK

>Cm_EDQM3
MCSRQEKDHCHKQDHCHSSGSSCHGSGGSSCHSGGSSCHSSGGSSCHGGGSSCHSGGSSCHGKPQQHCQQQQQQKICKVPCQK
LK

>Cm_EDQM4_partial
MCSRQEKDHCHSSXXXXXXXXXX

>Cm_EDWM1
MIYSSGRESYFNRNSTWYDPAGSWLDTRRTPFTYAYSTCCSSGCGPRGGHDNRCYEYRRSGCAENCHGSSGSCHGSGGHCCVR
RPSYFHGSSGGCHGHGWSVCSERSCHSSGSSCHGSGSSCHGSGSSCHGTSGACHSAPIYVKPKQHVQQCCPPVQKCCPPMQQC
CLPVKKC

>Cm_EDWM2
MIYSSGRESYFNRNSTWYDPAGSWLDTRRTPFTYAYSTCCSSGCGPRGGHDNRCYEYRRSGCAENCHGSSGSCHGSGGHCCVR
RPSYFHGSSGGCHGHGWSVCSERSCHSSTVLEHHATVLDRHATVPLEHATVHQFM

>Cm_EDYM1
MSYFAYQYKQRNYTPYSATRLVPPAEPCVVKGPAPPGTKCAETCAVKHPAPCTTQCRDPCAAKPSVPCATKCFEPHAQRHPAQ
YIPKFSEPVGVKCSTPCVTRYHEPYGLIHPQPFPERWNPCAPPYVHGGYPQACGPTYVPSFPKYPYPYAPQWPDTWGYGNCGP
C

>Cm_LOR_partial
MCSHQEKQDCREIPAQAGGHHASGAGSSGSCPGALRGQHILGSSGCGGGGGSSCCSSGESCQKIIITGGGSGGSSGGSGGSYG
CGVGGGSGIGSGGSGQKIIIADGGSGGLSGCGVGGGSGSGSGGSYGWGVGGGCGGEGXXXXXXXXXX

>Cm_PGLYRP3
MSLCILDFIVWFFCLHIVTPGKWGGRPANCSSPLKSVQPGYVIVLHTAGGSCKTRAECNQQMTNIQDYHMNNKGWCNIAYNFL
IGEDGKVYEGRGWHTEGAHTYGFNDISLGIAFIGDFTGRSPNAAAWKALKHLLHFAVENGYLSSNYLLMAHGDVSNTLSPGQP
IRKTLKKWPHYKH

>Cm_SCFN_partial
MPQLLDSISTIISVFYKHGKKDEDCSTISKREVKRFIQREFDNITVNPYDAHTIEAVLQLLDRDGDGAVDFNEFLLLVFRVAK
VCYWYLQPKQHLPQRTETGLSGERRQEPEAGRAEGSRDQPEPPGTEKGYYETRESETREIERSCRQPREPEPRGDQGGHYEIR
EPKPRENERSRHQLGEPEPRGDERRRYETREPEPREEERRHPQPREPEPRGNERSHREPFEQPREPEPRGESPXXXXXXXXXX
PHEPEPREDESRRRQPREPEPREDERSHRQPREPQPREEERSRRQPHEPEPREDESRRRQPREPEPREDERSHRQPREPQPRE
EERSRRQPHEPEPREDESRRRQPREPEPREDERSHRQPREPQPREEERSRRQPHEPEPREDESRRRQPREPEPREDERSHRQP
```

REPQPREEERSRRQPHEPEPREDESRRRQPREPEPREDERSHRQPREPQPREEERSRRQPHEPEPREDESRRRQPREPEPRED
ERSHRQPREPQPREEERSRRQPHEPEPREDESRRRQPREPEPREDERSHRQPREPQPREEERSRRQPHEPEPREDESRRRQPR
EPEPREDERSHRQPREPQPREEERSRRQPHEPEPREDESRRRQPREPEPREDERSHRQPREPQPREEERSRRQPHEPEPREDE
SRRRQPREPEPREDERSHRQPREPQPREEERSRRQPHEPEPREDESRRRQPREPEPREDERSHRQPREPQPREEERSRRQPHE
PEPREDESRRRQPREPEPREDERSHRQPREPQPREEERSRRQPHEPEPREDESRRRQPREPEPREDERSHRQPREPQPREEER
SRRQPHEPEPREDESRRRQPREPEPREDERSHRQPREPQPREEERSRRQPHEPEPREDESRRRQPREPEPREDERSHRQPREP
QPREEERSRRQPHEPEPREDESRRRQPREPEPREDERSHRQPREPQPREEERSRRQPHEPEPREDESRRRQPREPEPREDERS
HRQPREPQPREEERSRRQPHEPEPREDESRRRQPREPEPREDERSHRQPREPQPREEERSRRQPHEPEPREDESRRRQPREPE
PREDERSHRQPREPQPREEERSRRQPHEPEPREDESRRRQPREPEPREDERSHRQPREPQPREEERSRRQPHEPEPREDERSR
RQPREPEPREEERSRHQPQEPESREDEGSCCHPRKPEPREDGGSHLQPPEPESREDERSHHQPREPESQEDESSLHQPREPEP
REDERSRRQPNEPERREGERCSIQPQTPEPPHEEGYLPPSSEPEAREDLGTLHEPNDPESTDDYRKFPATHEPSPTGDEGSQL
QPRESALPGDEGSPQKRRESEPREDDGSQPQLCEPEHREGDGVLRHTPESQPLEEAGSQEQPHDLEPRTEGNRHQLPQGGEAS
HVQRDAEPQQGNGSRHQPREPQEESERSSHQAREPLPQLGEEPQGEWSQSHPEPANAEEEPGETDPDEAKASLPCNPLYVYLL
EQTVEKQLYLAPPCQKRP

B

>Ps_EDAA1_partial
XXXXXXXXXXGCYPYPCRWGCGYGSGNCWPCFAAEE

>Ps_EDAA2_partial
XXXXXXXXXXGCYPYPCRWGCGYGSGNCWPCFAAEE

>Ps_EDAA3_partial
MTFHHQKISRPWGCDPCCYGGWGGCRGRYDCYRAGXXXXXXXXXXAACSSSPSRWGCGYGYGNCWPCFAEQE

>Ps_EDAA4_partial
MTFHHQKISRPWGCDPCCYGGWGGCRGRYDCYRAGXXXXXXXXXXSHPCSSSPSRWGCGYGYGNCWPCFAEQE

>Ps_EDAA5
MTFDGSIIELCYNPWSHGCWHGSRSHFSYTRPCGNWGASRGGWNHVYDCSRPCSSQWGHWYPCGKHWPC

>Ps_EDAA6
MTYQHQKISHCWGYSPCSYGSGGGYRGHYDCYRPGCYSRPYRCGWGYDNGCYYPSSSRWGHGCGYQYGRCWPC

>Ps_EDAA7
MTFDDSPISELCYCPGSYGGCCGYGGSGYCRPWCYPRPYKCCWRRQYKCCYPYPYRWGCGYGSGNCWPCFAAEE

>Ps_EDAA8
MIFDENFRDELYYWSYHYGGGSRGYGCCRPCFAQEE

>Ps_EDbeta1
MSCSRSVCAEGAAACVAPPQPCGDTWTQPCVTQCPDSRVVIFPPPVVVTFPGPILTTFPQESVVDSAGTPAVASGYGGSHGAG
ALGLGRGACGLCGPC

>Ps_EDKM_partial
MSKFIKAITDLISSYQDNSRKGRESERFQRCXXXXXXXXXX

>Ps_EDP1L1_partial
MTYYGRKHQQHCLPSPACVAKCPQPCRPQYEQHCAPKCQPVYVTKCTPLYXXXXXXXXXXCVTKCPPQCVTKCPPPCVTKCPP
QCVTKCPPPCVTKCPPQCVTKCPPPCVTKCPPRCVTKCPQQCVTQYPGQCQSGNIKMSSQCKKYCSAPNWPW

>Ps_EDP1L2
MTYYGRKHQQHCLPSPACVAKCPQPCRPQYEQHCAPKCQPVYVTKCPPLYGPQYAFPCAAQCPPRCVTKCPPPCVTKCPPQCV
TKCPPPCVTKCPPQCVTKCPPPCVTKCPPQCVTKCPPPCVTKCPPQCVTKCPPPCVTKCPPQCVTKCPPPCVTKCPPQCVTKC
PPPCVTKCPPQCVTKCPPQCVTKCPPRCVTKCPQQCVTQYPGQCQSGNIKMSSQCKKYCSAPNWPW

>Ps_EDP2
MASPQNQQQRRQSLPLPPALSNAAPEPEPSPGPRTVKEPENAPREEEKPQKEPLDQPPGPVPELEPEPEPEPAPEPNPPEA
EEAGYLQPEQQQYKQPPALPPAPGAETSTECEEAKPEPEPEPGRCPPPISEPEGPGPVQPSPPGEEKQQKQPCRWPPARK

>Ps_EDP4
MSSQQHQCKQTCPPPKCQEKCPPPCKEPCPPPKCPSPCKEPCPPPKCQDPCKEPCPPPKCPSPCKEPCPPPKCPSPCKEPCPP
PKCPSPCKEPCPPPKCPSPCKEPCPPPKCPSPCKEPCPPPKCPSPCKEPCPPPKCPSPCKEPCPPPKCPSPCKEPCPPPKCPS
PCKEPCPPPKCPSPCKEPCPPPKCPSPCKEPCPPPTPPPLSPSGFLFVSLFLPPPFTLLPRDPVSHKGWGGGAGRKLCVSAEA
RGSAGAP

>Ps_EDP5
MPHHQQQCKQPCQPPPVCPPLCQETCPTPKCPEPCSPKCPEPCSPKCPEPCSPKCPEPCSPKCPEPCSPKCPEPCSPKCPEPC
SPKCPEPCPKPCPEPCPEPKCPEPKCPEPKCPEPCPPPHCQEKCPPPHCQEKCPPPHCQEKCPPPHCQEKCPPPHCQEKCPPP
HCQEKCPPPHCQEKCPPPKCPPTQK

>Ps_EDP6
MSSQQHQCKQTCPPPKCQEKCPPPCKEPCPPPKCPSPCKEPCPPPKCPSPCKEPCPPPKCPSPCKEPCPPPKCPSPCKEPCPP
PKCPSPCKEPCPPPKCPSPCKEPCPPPKCPSPCKEPCPPPKCPSPCKEPCPPPKCPSPCKEPCPPPKCPSPCKEPCPPPKCPS
PCKEPCPPPKCPSPCKEPCPPPKCPSPCKEPCPPPKCPSPCKEPCPPPKCPSPCKEPCPPPKCPSPCKEPCPPPKCPSPCKEP
CPPPKCPSPCKEPCPPPKCPSPCKEPCPPPKCPSPCKEPCPPPKCPSPCKEPCPPPKCQDKWEQTLPGTMVPATLTQLPLSAA
SHTAPCLAFNYGKRRPPAECGVPHSYPLGGGLTQTDRHTQLPAETWPCVGLLSASPRPVSQASKNQGRSLQAST

>Ps_EDPCV1
MSHQQQCKQTCPPPPCCVKHCETKCVDPCPCPPKCVDPCPCPPKCPPCCPCPPCPPKCIDPCPCPPKCVDPCPCPPKCPPCPP
CPPVQQCCQEKKSC

>Ps_EDPCV2
MAHQQQCKQTCPPPQCCVTKCTTKCLDPCCNVCETKCVTKCVDPCCNVCVKKCTTCVHPCPCPPKCVDPCPCPCPPKCVDPCP
CPCPPKCVDPCPCPCPPKCVDPCPCPCPQKCVPCPPKCPPVQQCCKEKRLC

>Ps_EDPCV3
MAHQQQCKQTCPPLQCCATKCTTKCLDPWCNICGTKCVAKCTDPCCNVCVKKCTKCVHPCPCPPKCVDSCPCPCSCPQKCPPC
PLICPPVQQCCKEKKLC

>Ps_EDPCV4
MAHQQQCKQTCPPPQCCVTKCTTKCLDPCCNVCETKCVTKCVDPCCNVCVKKCTTCVHPCPCPPKCVDPCPCPCPCPCPQKCV
PCPPCPPKCPPVQQCCKEKRLC

>Ps_EDPL1
MSCQQPQQQCKQACMPPPCKEPCLPKTTEPCPQQCTEPCPQQGTEPCPPKCAEPRPGPRRDPRLSSSARTAAPRINCSRRARR
RQIPTREPSPLAPGCFTVALLVFQLRTGLAAGTGRWEGFGTDTGSQAAQF

>Ps_EDQL
MCSREPRGCPDSERSSCPSSERSSCHGSEATTCHDVKPHPQYPTTVPCQTPTSPCQQQTKQTCPWPPQKHQK

>Ps_EDQM1_partial
XXXXXXXXXXGSGGGSCHGKPQKPCVPDQQQHKDCCQVPSQKLK

>Ps_EDQM2
MCSRQGGDHCHSSDSCLSSGSSCHGSGGSSCHSSGGSSCHGGGSSCHSGGSSCQGGGSSCHSGGSSCHGGGSSCHGGGSCHGK
PQQPCQQQQPQQQQKVCNVPCKKLK

>Ps_EDYM1
MSYFAYQYKQRNYTPYSTTRLLACAEPCVVKGPAPCGTKCVEPCATKRPAPCVPKCRDPCAGKAPVHCEPKCLEPHAQRGPAH
CAPKFSEPAGVKCSVPWVPRCHEPYGPIPARPFPERWNPCAPPYGQPFVTGYPQACGPSYGPSFPKYSYPCAPQWPGGWGYGG
CGPC

>Ps_LOR_partial
MCSHQQKQDCHEIPAGPGGDCSCTGGGSVGGGSGAVLHQSTLGNSSGGSACCGVGGGSGGVGGIAAGGCLGGGSGGVGGGPGQ
KGVVCGGSGGXXXXXXXXXX

>Ps_PGLYRP3
MSQLAVLLWAVSAVSWGLSCLHIITPGKWGGRPANCSTPLKDVQTGYVVTLHTAGRSCSTQAECNQELLNIQQHHMNSKGWCN
LAYNFLIGEDGNVYEGRGWNTEGAHTYGSPNAAAWVALKRLLHFAVEAGYLSSNYLIMAHGDVSNTISPGQPIPNVLKTWPHY
KH

>Ps_SCFN
MPQLLDSISTIISIFCTQGEKDEGCSTVSRREMKRFLQREFADVTLTPYDPPTIEAVLQLLDHDEDGAVDFNDFLLLVFRVAK
VCYWYLQPKQRLPPRAEKEMSGKQGPAPKAGRAKGSCEQPCEKGGYEMPEPEARETEKSHWQPHETEPRRDQGGRHKIHEPKP
RENSRSHHQRGEPELQGDESWSYEPCEPEPREAETRRHQPRESEPRGDERSCCEPFEYEPKPSEHPHEPEPRGAESWCYEPHE
PEPMEEERYRHQPREPKPQGDERRCHEIRVSEERENDRRHHQPRESEPQGAERSWAGACEPEAREEERHHPQPRQPEIQKDEW
RHYGIRDHERREVDRRQQHLHDPEWRGDDRSSHQPREPEPQGYERRRSEQEPIEGGKSHYWPREQKPRGGAKTHYQPSRPEQR
RDERHGYERCDREARREESRHQPREPELRGAERHHYETDAAELRENDGTSHQSHETQPREDETRLYDRHILQRRNRDEHCPQR
RESGTREDKMSHSERHAPKSQGAKRRHHQPNSPEHQGDDRYSHERCETEYRRNDKRHNQPGQPKLRRDERSHYQSWEPELRVD
KWSCRQPREPEPREDDKSHRQPCDPEPREGERSCHQPRPREDKRSLHLPHEPESREDGSSPRQPWQPEPREDDKSHHQPCDPE
PREGERSCHQPRPREDKRSLHLPREPESREDGSSPRQPWQPEPREDDKSHHQPCDPEPREGERSCHQPRLREDKRSLHLPREP
ESREDGSSPRQPWQPEPREDDKSHRQPCDPEPREGERSCRQTHPRKDKRSLHLPREPESREEERRHQPSEFEPREAEQSLHQP
HELEPKGEERSHRQPHEPEPREEEQSLHQPREPEPRGEERSHRQPHEPETTEKERSLHKPHEPELREERSYRQPLDPEPREEE
QSLHRPHEPELRGEERRHRPRDFEHREDEQSLHQPREPEPRGEEKSLHQPDPEPRKDEQSHHQPLDPEPRKDEQSHHQPLDPE
PRKDEQSHHQPLDPEPRKDEQSHHQPLDPEPRKDEQSFHQPHELEPKEGERSHQQPNKPEPREEESSH

RQPREPEPREEQRRCHQPPEPEAGVEEVSQQQPGSHEPEQEKGHLPLWREPEAREDVGTQRDADAPGDYTRCPAMPKPSPTGE
VGIPLQPWDGSLAGGEGSRQAATDSERREDSQPQLREPEHREEDGILRETPESEPRAATGSQHQPCDLEGQREGSRPQRPPGG
DRSCLPLDTEPPLANRSLPCPKEPQEHASKRSSPQASELQPWLGEAPQEGEWSQSHPESASSQEEPDETGAEDAKACLPCSPL
YVYLLEQTVEKQLYLAPPHQEWP

C

>Ps_Beta-1
MSCYPECGVARPSPVSGTCNEPCVRQCPDSEVVIRPSPIAVTIPGPIMSTFPQQSEVGAVGAPVVGSGYGGSFGAGALSGYGA
PYGGLYGLGGFGGYGGHFGGLCGYGGRYGYGGLNGYGRGFGGLCGYGGGYGGLCGYGGGYGGLCGYGGGYGGLCGYRGGYGYG
GACGTGVSCHRYLSACGSPC

>Ps_Beta-2
MSCYSECGVARPSPVSGTFNEPCVRQCPDSQVLIMPSPIAVTIPGPIMSTFPQHSEVGAVGAPVVGSGYGGSFGAGGLFGSGA
GYGGLFGLGGSGGYGGLCGYGGRYGYGGGYGTGVSCHRYLSACGSPC

>Ps_Beta-3
MACSSLSYPECGVARPSPVSGTFNEPCVRQCPDSHVLIRPSPIAMTIPGPIMSTFPQHSEVGAVGAPLVGSGYGGSFGAGGLF
GSGAGYGGLSVLGGSGGYGGLCGYGGRYGYGGGYGSGVSCHRYLSACGSPC

>Ps_Beta-4
MSCYPECGVARPSPVSGTFNEPCVRQCPDSQVLIRPSPIAMTIPGPIMSTFPQHSEVGAVGAPFVGSGYGGSFGAGGLFGSGA
GYGGLFGLGGSGGYGGRYGYGGGYGYGGSCGTGVSCHRYLSACGSPC

>Ps_Beta-5
MSCYPECGVARPSPVSGTFNEPCVRQCPDSQVLIMPSPVAMTIPGPIMSTFPQHSEVGAVGAPLVGSGYGGSFGAGGLFGSGA
GYGGLSGLGGSGGYGGLCGYGGRYGYGGGYGIGLSCHRYLSACGSPC

>Ps_Beta-6
MSCYSECGVAQPRPVSGTFNEPCVRQCPDSHVLIMPSPIAVTIPGPIMSTFPQHSEVGAVGAPLVGSGYGGSFGAGGLFGSGA
GYGGLLGLGGSGGYGGLCGYGGRYGYGGGYGSGVSCHRYLSACGSPC

>Ps_Beta-7
MIKSLCAPRCYPCPDICPDPCAYVCNEPCVTSCGDSTAVVYAPPVAIRFPGPILATCPQDSVMGTSLPQIPYGPYGGGAVFAL
GVL

>Ps_Beta-8
MSSHKDLCCPRPSCCPDVCPQPYVDAWNQPCVTSCGDSSAVVYPPPVIVRFPGPILATCPQESFVGTSLPNVPYGYGGSYAGG
NFGGSVSAGSVSGGTYGGLYSYGKRYDRKCYPSRFGGCGPC

>Ps_Beta-9
MSSCKDLCLPRPACCPNVCPQPYVDAWNQPCVTSCGDSSAVVYPPPVIVRFPGPILATCPQESFVGTALPNVPYGYGGSYGGG
SISGSVGSGHVSGGAYGGGYAGFYGYGSKYDRKCSSSLFGRCGPC

>Ps_Beta-10
MASRKDLCCPRPPCCPDICPQPYVDAWNGPCVTSCGDSSAVVYPPPVVVRFPGPILATCPQDSVVGTALPNVPYGYGGSYGGS
NFGGSLSSGSVSGGAYGGLCSYGKRYERKCYSSRFGGCGSC

>Ps_Beta-11
MSSRKELCCPRPQCYPDVCPQPYVDAWNGPCVTSCGDSSAVVYPPPVVVRFPGPILATCPQESVVGTALPNVPYGSGGAYAGG
KFGGSVSSGGVYGRGYTGGYGAGYGGLFGDGSKYGRNCYSSRFGGCGPC

>Ps_Beta-12
MASRKDLCCPLPPCCPDICPQPYVDAWNGPCVTSCGDSSAVVYPPPVVVRFPGPILSTCPQDSVVGTALPNVPYGYGGSYRGS
NFGGSLGSGSVSGGAYGGLCSYGKRYERKCYSSRFGGCGPC

>Ps_Beta-13
MSSRKELCCPRPQCYPDVCPQPYVDAWNGPCVTSCGDSSAVVYPPPVVVRFPGPILATCPQESVVGTALPNVPYGSGGAYAGG
KFGGSVSSGGVYGRGYTGGYGAGYGGSFGDGSKYGRNCYSSRFGGCGPC

>Ps_Beta-14
MSSRKELCCPRPQCYPDICPQPYVDAWNGPCVTSCGDSSVVVYPPPVVVRFPGPILATCPQESVVGTALPNVPYGSGGAYAGG
KFGGLAGSGGAYGSGYTGGYGAGYGGSFGDGSKYGRNCYSSRFGGCGPC

>Ps_Beta-15
MSSCRELCYQPSPCWPDICPDPCAVARNEPCITSCGDSTAVVYPPPVSVLFPGPILSTLPQHSVVGSTLPAIPYGARSSSGGG
ILGGSLGYGGGYDGAYGGGYGGGIGAFGYGGLCGYGKRYGRRCYPYRCGPCWPC

>Ps_Beta-16
MSFCRDLCPSPSYPACQVTCPQPFVDACNGPCVTSCGDSTAVVYPPPVIVNFPGPILATCPQESIVGSSEPLGIGSAIGYGGS
NLSVSSYGYRPSLGYGGSSGSQSLNSLRRSYTSGVSSVGRGGSDPCSSRWLMMYGCGPRPTQQH

>Ps_Beta-17
MSTARLWLNNLNSEDSEQDQDSKTKDPPKDCPKGCKCPKCEPCKPCPRPCPRPCPRPCPPCWPCPRPCPPRCPPICPQPCVNV
CNEPCVTSCGDSTAVVYAPPVQVIFPGPILATCPQDSVVGSSLPAGMFGPRPSGGFLGAGSSVGSGGYLGAGGYLGAVGSFGP
GGCVGGGGSSFGTVCCNPCRYGSCWPWSSQEGSPRKTTLPGNDKRAADSRLS

>Ps_Beta-18
MSTARLWLNNLNSEDSEQDQDSKTKDPPKDCPKGCKCPKCCKCPPIKPCKPYPCFPRPCPRPCPPCWPCPRPCPPRCPPICPQ
PCVNVCNEPCVTSCGDSTAVVYAPPVQVIFPGPILATCPQDSVVGSSLPAGMFGPRPSGGFLGAGSSVGSGGYLGAGGYLGAV
GSFGPGGCVGGGGSSFGTVCCNPCRYGSCWPC

>Ps_Beta-19
MSFQGPATCTQSSQPCEVKCGEPCVLTHNEPCVVQCPDTRVIIYPPPVVVTFPGPILTTCPQESIVTAAGPPDISLAESSGMI
SSATGGSGSVGPQIHGCAPKATAGLESRYTSKYSSTSRLSGQGLGESYCYGNRQL

>Ps_Beta-20
MSFYGPCNVPCEGPHPMPSAVTYHEPCVIQCPDSMHETDSPPGVAIIPGPTLTTLSHYSLVGSSALLDMERPFGPHMCLGSEG
CMNSFD

>Ps_Beta-21
MSFYGPCNVPCEGPHPMPSAVTYHEPCVIQCPDSMHETDSPPGVAIIPGPTLTTLSHYSLVGSSALLDMERPFGPHMCLGSEG
CMNSFD

>Ps_Beta-22
MYSSMAFIGKDQKPQEPQQDEKTCPPPHKRCPPPEKTCPPPQKTCPPPCPPCPPCPKCPPCPPCRPCPPLCPPRCPLICPEPC
INVCNEPCVTGCGDSTALIFAPPVRVIFPGPILATCPQDSVVGSSLPFGVLGPRRAGGSLGSGGAFGAGSSFNAGGYLGSGRY
LGSGGSSFGTVCCNPCSCVRPRPC

>Ps_Beta-23
MYSTMALIGKDQKPREPQQDQKIPPPHKGCPPCPECPPQCPPCPKCPPCPKCPPCPKCPPCPKCPPCPKCPPCPKCPPCPKCP
PCPKCPPCPKCPPCPKCPPCPKCPPCPPCRPCRPLCPPRCPPICPEPCINVCNEPCVTSCGDSTAVVFAPPVRVIFPGPILAT
CPQDSVVGSSLPFGILGPRRAGGSLGSGGAFGAGSSFNAGGYLSSGGYLGSGGSSFGTVSCNPCSCVRPWPC

>Ps_Beta-24
MYSTMALIGKDQKPREPQQDQKIPPPQKCCPPCPECPPPCKPCPPCPPCPPCPPCPKCPPCPPCRPFCPPRCPAICPEPCINV
CNEPCVTSCGDSTAVVFAPPVRVIFPGPILATCPQDSVVGSSLPFGILGPRRAGGSLGSGGAFGAGSSFNAGGYLSSGGYLGS
GGSSFGTVSCNPCSCVRSWPC

>Ps_Beta-25
MSTAKLWLNNLNSEDSHQDQDTKTKHQKKCSPDCLPCPQDPQCPECPKCPDPSKCPDCLPCPQDPQCPECRKCPDPSKCPDCL
PCPQDAQCPECPKCPVPPKCPDCLPCPQDPQCPECPKCPDPPKCPDCLPCPQDPQCPECPKCPDPPKCPDCLPCPQNHQCPEC
PKCPDPCKCPECRPCPQDPQCPECPKCPDPPKCPDCLPCPQNHQCPECPKCPDPCKCPECRPCPQDPQCPECPKCPYPPKCPN
CLPCPQDPQCPECPQCPDPSKCPECPKCPNPPKCPDCPKCPEPTKCPDPSKCPDCPKCPPCLPCPLCRPCPRLCPPRCPPICP
PPCVNVCNEPCVTHCGDSTAVVYAPPVQVIFPGPILSTCPQDSVVGSSLPAGMFGPRPSGGLLGAGGLLGLGGSFGSGGCFGG
GGSSFGTVRCNPCRCGRCWPC

>Ps_Beta-26
MSRPICPECPKCPDPSKCPDCLSCPQDPQCPKCPKCPNPSKCPDCFPCPQDPQCPECPKCPYPPKCPDCLPCPQDPQCPECPK
CPEPPKCPDCPKCPEPTKCPDPSKCPDCPKCPPCLPCPPCRPCPRPCPPRCPPICPPPCVNVCNDTCVTHCGDSTAVVYAPPV
QVCGDSTAVVYAPPVQVIFPGPILSTCPQDSVVGSSLPAGMFGPRPSGGLLGAGGLLGLGGSFGSGGCFGGGGSSFGTVRCNP
CRCGRCWPC

>Ps_Beta-27
MSTAKLGLNDLKSQDSQQDQDTKTKDPTKGCPECPQRTECPKGCNCPKCCECPKCEPCEPCPPCPRPCPRPCPPCWPCPRPCP
PRCPPICPSPCVNVCNEPCVTRCGDSTAVVYAPPVQVIFPGPILSTCPQDSMVGSSLPAGMFGPRPSGGYLGAGGSVGPRGSF
GPGGCVGGGGSSFGTVCCNPCHYGRRWPC

>Ps_Beta-28
MACSSLSYPECGVARPSPVSGTFNEPCVRQCPDSHVLIMPSPVAMTIPGPIMSTFPQHSEVGAVGAPLVGSGYGGSFGAGGLF
GSGAGYGGLSGLGGSGGYGELCGYGGRYSYGGGYGTGLSCHRYLSACGSPC

>Ps_Beta-29
MACSSLSYPECGVARPRPVSGTFNEPCVRQCPDSHVLIMPSPIAVTIPGPIMSTFPQHSEVGAVGAPLVGSGYGGSFGAGGLF
GSGAGYGGFSGLGGAGGYGGLCGYGGRSGYGGGYGTGVSCHRYLSARGSPC

>Ps_Beta-30
MSCYSECGVARPRPVSGTFNEPCVRQCPDSQVLIMPSPVAVTIPGPIMSTFPQHSEVGAVGAPLVGSGYGGSFGAGGLFGSGA

GYGGLFGLGGSGGYGGLCGYGGRSGYGGGYGTGVSCHRYLSACGSPC

>Ps_Beta-31
MACSSLSYPECGVARPRPVSGTFNEPCVRQCPDSHVLIMPSPIAVTIPGPIMSTFPQHSEVGAVGAPLVGSGYGGSFGAGGLF
GSGAGYGGLSGLGGSGGYGGLCGYGGRYGSGRGYGTGVSCHIKAKGKTKD

>Ps_Beta-32
MSCYTECGVARPSPVSGTFNEPCVRQCPDSQVLIMPSPVAMTIPGPIMSTFPQHSEVGAVGAPLVGSGYGGSFGAGGLFGSGA
GYGGLFGLGGSGGYRELCGYGGRYGYGGGYGTGVSCHRYLSACGSPC

>Ps_Beta-33
MSCYTECGVARPSPVSGTFNEPCVRQCPDSQVLIMPSPVAMTIPGPIMSTFPQHSEVGAVGAPVVGSGYGGSFGAGGLFGSGA
GYGGFSGLGASGGYGGLCGSPVVGSGYGGSFGAGGLFGSGAGYGGLFGLGGSGGYGGLCGYGDRYGYGGGYGSGVSCHRYLSA
CGSPC

>Ps_Beta-34
MSCYSECGVARPRPVSGTFNEPCVRQCPDSQVLIMPSPIAMTIPGPIMSTFPQHSEVGAVGAPLVGSGYGGSFGAGGLFGSGA
GYGGLSGLGGSGGYGELCGYGGRYSYGGGYGTGLSCHRYLSACGSPC

>Ps_Beta-35
MACSSLSYPECGVARPRPVSGTFNEPCVRQCPDSHVLIMPSPIAVTIPGPIMSTFPQHSEVGAVGAPVVGSGYGGSFGAGGLF
GSGAGYGGLFGLGGSGGYGGLCGYGGRYGYGGGYGSGVSCHRYLSACGSPC

>Ps_Beta-36
MACSSLSYPECGVARPRPVSGTFNEPCVRQCPDSQVLIMPSPVAMTIPGPIMSTFPQHSEVGAVGAPLVGSGYGGSFGAGGLF
GSGAGYGGFSGLGGAGGYGGLCGYGGRYGYGGGYGTGVSCHRYLSACGSPC

>Ps_Beta-37
MSCYTECGVARPRPVSGTFNEPCVRQCPDSQVLIMPSPVAMTIPGPIMSTFPQHSEVGAVGAPLVGSGYGGSFGAGGLFGSGA
GYGGLSGLGGSGGYGGLCGYGGRYGSGRGYGTGVSCHRYLSACGSPC

>Ps_Beta-38
MSCYPECGVARPSPVSSTCNEPCVRQCPDSEVVIRPSPIAVTIPGPIMSTFPQQSEVAALGAPVVGSGYGGSFGAGALSGYGA
PYGGYYGLGGLGGYGGRFGYGGLCGYGGGYGYGRGFGGLCGYGGGYGGLCGYRGGYGYGGSCGTGVSCHRYLSGSCTPC

>Ps_Beta-39
MSCYPECGVARPSPVSGTCNEPCVRQCPDSEVVIRPSPIAVTIPGPIMSTFPQQSEVGAVGIGAPVVGSGYGGSFGAGALSGY
GAPYGGYYGLGGFGGYGGRFGYGGLCGYGGGYGYGRGFGGLCGYGGGYGGLCGYGGGYGGLCGYRGGYGYGGSCGTGVSCHRY
LSACGSPC

>Ps_Beta-40
MSCYPECGVARPSPVSGTCNEPCVRQCPDSEVVIRPSPIAVTIPGPIMSTFPQQSEVAALGAPVVGSGYGGSFGAGALSGYGA
PYGGYYGLGGFGGYGGRFGYGGLCGYGGGYGYGRGFGGLCGYGGGYGGVWGEVTVMEDLVAPGYLVIGT

>Ps_Beta-41
MSCYTECGVARPRPVSGTFNEPCVRQCPDSQVLIMPSPVAMTIPGPIMSTFPQHSEVGAVGAPLVGSGYGGSFGAGGLFGSGA
GYGGLFGLGGSGGYGGLCGYGGRYGYGGGYGTGVSCHRYLSACGSPC

>Ps_Beta-42
MSCYPECGVARPSPVSGTCNEPCVRQCPDSEVVIRPSPIAVTIPGPIMSTFPQQSEVAALGAPVVGSGYGGSFGAGGLFGSGA
GYGGFSGLGASGGYGGLCGYGGRYGYGGGYGTGVSCHRYLSACGSPC

>Ps_Beta-43
MSCYPECGVARPSPVSGTCNEPCVRQCPDSEVVIRPSPIAVTIPGPIMSTFPQQSEVAALGAPVVGSGYGGSFGAGALSGYGA
GGYGGLCGYGGGYGGLCGYGGGYGGLCGYGGGYGGLCGYGGGYGGLCGYGGGYGGLCGYGGGYGGLCGYGGGYGGLCGYGGGY
GGLCGYRGGYGYGGACGTGVSCHRYLSACGSPC

>Ps_Beta-44
MACSSLSYPECGVARPSPVSGTFNEPCVRQCPDSHVLIMPSPVAMTIPGPIMSTFPQHSEVGAVGAPLVGSGYGGSFGAGGLF
GSGAGYGGLSGLGGSGGYGELCGYGGRYSYGGGYGTGLSCHRYLSACGSPC

>Ps_Beta-45
MSCYPECGVARPSPVSGTCNEPCVRQCPDSEVVIRPSPIAVTIPGPIMSTFPQQSEVGALGAPVVGSGYGGSFGAGALSGYGA
PYGGMYGLGGLGGYGGHFGYGGLCGYGGGYGYGRGFGGLCGYGGGYGGLCGYGGGYGGLCGYRGGYGYGGSCGTGVSCHRYLS
GSCTPC

>Ps_Beta-46
MSCYPECGVARPSPVSGTCNEPCVRQCPDSEVVIRPSPIAVTIPGPIMSTFPQQSEVAALGAPVVGSGYGGSFGAGALSGYGA
PYGGYYGLGGFGGYGGRFGYGGLCGYGGGYGYGRGFGGLCGYGGGYGGLCGYGGGYGGLCGYRGGYGYGGSCGTGVSCHRYLS
GSCTPC

>Ps_Beta-47
MACSSLYYPECGVARPSPVSGTCNEPCVRQCPDSEVVIRPSPIAVTIPGPIMSTFPQQSEVAALGAPVVGSGYGGSFGAGALS
GYGAPYGGYYGLGGFGGYGGRFGYGGLCGYGGGYGYGRGFGGLCGYGGGYGGLCGYGGGYGGLCGYGGGYGGLCGYRGGYGYG
GACGTGVSCHRYLSGSCTPC

>Ps_Beta-48
MSCSSLYYPECGVARPSPVSGTCNEPCVRQCPDSEVVIRPSPIAVTIPGPIMSTFPQQSEVAALGAPVVGSGYGYGAPYGGMY
GLGGLGGYGGHFGYGGLCGYGGGYGYGRGFGGLCGYGGGYGGLCGYGGGYGGLCGYRGGYGYGGSCGTGVSCHRYLSGSCTWP
KEP

>Ps_Beta-49
MSCYPECGVARPSPVSGTCNEPCVRQCPDSEVVIRPSPIAVTIPGPIMSTFPQQSEVGAVGAPVVGSGYGGSFGAGALSGYGA
PYGGYYGLGGFGGYGGRFGYGGLCGYGGGYGYGRGFGGLCGYGGGYGGLCGYGGGYGGLCGYRGGYGYGGACG
TGVSCHRYLSACGSPC

>Ps_Beta-50
MSCYPECGVARPSPVSGTCNEPCVRQCPDSEVVIRPSPIAVTIPGPIMSTFPQQSEVGAVGAPVVGSGYGGSFGAGALSGYGA
PYGGLYGLGGFGGYGGHFGGLCGYGGRYGYGGLNGYGRGFGGLCGYGGGYGGLCGYGGGYGGLCGYRGGYGYGGACGTGVSCH
RYLSACGSPC

>Ps_Beta-51
MACVPQDCGSDICPRPYIDVCNSPCVSSCGDSTAVVFAPPVVVRFPGPTLATCPQDSIVGSALPQLPYGPGGFPGVGGGVGGP
FAGGYGGVSGGRFGGNYGGYSGGSGGGYAAGGCGGGYSGGYGGSCGSRRSYRSISSCGGGYSSKGGCGPC

>Ps_Beta-52
MACIPQNCCLDICPCPYIDVCNSPCSSSCSDSTAVVYAPPAVVRFPGPTLATCPQDSFVGSVLPQLPSGPGGFPGDGGGVGGS
LGSGG

>Ps_Beta-53
MACVPQDCGSDICPRPYIDVCNSPCISSCGDSTAVVFAPPVVVRFPGPTLATCPQDSFVGTSLPQLPSGPGGFPGVGGGVGGA
YGGRFGGNYGGYSGGYRGGYRGGYGGGCGGGYCGGNGGSCGFRKSYSSISSCGGGYSSKGGCGPC

>Ps_Beta-54
MACVPQDCGSDICPRPYIDVCNSPCVTSCGDSTAVVYAPPVVVRFPGPTLATCPQDSFVGTSLPQLPSGPGGFPGVGGGAGGA
YGGRFGGNYGGYSGGYRGGYRGGYGGGCGGGYCGGNGGSCGFRKSYSSISSCGGGYSSKGGCGPC

>Ps_Beta-55
MSRAPPVCVKFPGPTLATCPQESFVGTSIPYLAGGPGGFPGSGVGSRGPFTAKSSSSGVYAGGYNGGSGGGYVYGSGFQGGYG
GSCGYGRKLYGSRSVCGGQYSGYQGRNCGPC

>Ps_Beta-56
MKFPCAPRCYPCPDICPEPCAYVCNEPCVTSCGDSTAVVYAPPVAVRFPGPILATCPQDSVVGTTLPLPPYGPYRGGAGGGAG
SFLGGGGSGVFGGGSGGGAGGLGGLGGGYCYGSSSGGYGRHCSYTCVPCPRYRPC

>Ps_Beta-57
MTFPCAPRCHPCPDLCPEPCAVVCNDPCVTSCGDSTAVVFAPPVAIRFPGPTLATCAQDSVVGSSAPWLPYGPYGPSRGGAGG
GAGSGAGGGVYGGVFGGGVYGGALGGGSGSGFGGCSGGGYGGSFRQWSRYDRKCYANRYDCCPPC

>Ps_Beta-58
MKFPCAPRCHPCPDICPEPCAYVCNEPCVTSCGDSTAVVYAPPVAIRFPGPILATCPQDSVVGSSLPQIPYGPYGGGAGGGGA
LGGAGGLGGGALVGAGSGFGGGSGGGFGSGFGGCHGGYWGRRCYANRYDCCPW

>Ps_Beta-59
MKSLCAPRCYPCPDICPDPCAYVCNEPCVTSCGDSTAVVYAPPVAIRFPGPILATCPQDSVVGTSLPQIPYGPYGGGAVFALG
VL

>Ps_Beta-60
MAFSSLCYPECGVARPSPVSGTCNEPCVRQCPDSEVVIRPSPVAVTLPGPVLSTFPQQSEVAAVGEPVVGAGYGGSFANGGLY
GYGGRYGGLFGYGGYGYGGRYGYGGYPGFYGYGGYCGYPGFFGPC

>Ps_Beta-61
MAFSSLCYPECGVARPSPVSGTCNEPCVRQCPDSEVVIRPSPVAVTLPGPVLSTFPQQSEVAAVGEPVVGAGYGGSFANGGLY
GYGGRYGGLFGYGGYGYGGRYGYGGYPGFYGYGGYCGYPGFYGNGGYCGYPGYYGNGGYCGYPGYYGNGGYCGYPGYYGNGGY
CGYPGYYGNGGYCGYPGYYGNGGYCGYPGYYGNGGYCGYPGYYGNGGYCGYPGYYGNGGYCGYPGYYGNGGYCGYPGYYGNGG
YCGYPGYYGNGGYCGYPGYYGNGGYCGYPGYYGNGGYCGYPGYYGNGGYCGYPGYYGYGGVVGSGVSCHRYRSGSCAPC

>Ps_Beta-62
MAFSSLCYPECGVARPSPVSGTCNEPCVRQCPDSEVVIRPSPVVVTLPGPVLSTFPQQSEVAAVGEPVVGAGYGGSFANGGLY
GYGGRYGGLFGYGGYGYGGRYGYGGYWGRPLRLWGLMGLPRLLWEWGILRLPRLLW

>Ps_Beta-63_partial
MAFSSLCYPECGVARPSPVSGTCNEPCVRQCPDSEVVIRPSPVAVTLPGPVLSTFPQQSEVAAVGEPVVGAGYGGSFANGGLY
GYGGRYGGLFGYGGYGYGGRYGYGGYPGFYGYGGYCGYPGFYGYGGYCGYPGFYGNGGYCGYPGXXXXXXXXXX

>Ps_Beta-64
MAFSSLCYPECGVARPSPVSGTCNEPCVRQCPDSEVVIRPSPVAVTLPGPVLSTFPQQSEVAAVGEPVVGAGYGGSFANGGLY
GYGGRYGGLFGYGGYGYGGRYGYGGYPGFYGYGGYCGYPGFFGGGDCGYPGYYGNWGYPGYYGGLCGGYGGYGRRYLGGYCGP
C

>Ps_Beta-65
MAFSSLCYPECGVARPSPVSGTCNEPCVRQCPDSEVVIRPSPVVVTLPGPVLSTFPQQSEVAAVGEPVVGAGYGGSFANGGLY
GYGGRYGGLFGYGGYGYGGRYGFGGYPGFYGYGGYCGYPGFYGNGGFWGYPGRYAHGGYCGYPGYYGGLCGGYGGYGRRYLGG
YCGPC

>Ps_Beta-66
MAFSSLCYPECGVARPSPVSGTCNEPCVRQCPDSEVVIRPSPVAVTLPGPVLSTFPQQSEVAAVGEPVVGAGYGGSFANGGLY
GYGGRYGGLFGYGGYGYGGRYGYGGYPGFYGYGGYCGYPGFFGGGGFWGYPGRYAHGGYCGYPGYYGNGGYCGYPGFYGYGGV
VGSGVSCHRYRSGSWAPC

>Ps_Beta-67
MAFSSLCYPECGVARPSPVSGTCNEPCVRQCPDSEVVIRPSPVVVTLPGPVLSTFPQQSEVAAVGEPVVGAGYGGSFANGGLY
GYGGRYGGLFGYGGYGYGGRYGYGGLWGYPGFYGNGGYWGYPGRYAHGGYCGYPGFYGNWGYCGYPGYYGGLCGGYGGYGRRY
LGGYCGPC

>Ps_Beta-68
MAFSSLCYPECGVARPSPVSGTCNEPCVRQCPDSEVVIRPSPVVVTLPGPVLSTFPQQSEVAAVGEPVVGAGYGGSFANGGLY
GYGGRYGGLFGYGGYGYGGRYGYGGLWGYPGFYGNGGYWGYPGRYAHGGYCGYPGYYGNWGYPGYYGGLCGGYGGYGRRYLGG
YCGPC

>Ps_Beta-69
MTFSSMCYPECGVARPSPISVSCNEPCVRQCPDSHVTIIPTPVDVTLPGPILETHPQQSTVGAVEDPALGVGYGSSLGLGGLG
DNGGFYGLRKFGGFGGYLGGCGYGGYCGYPGYYGYGGYCGYPGYYGYGRYCGYPGFYGSRRLYGSGVSCHSYLGGYCGPC

>Ps_Beta-70
MTFSSMCYPECGVARPSPISVSCNEPCVRQCPDSHVTIIPTPVDVTLPGPILETHPQQSTVGAVEDPALGVGYGSSLGLGGLG
DNGGFYGLRKFGGFRGYLGGYRYGRYCGYPGYYGYGGYCGYPGYYGYGRYCGYPGFYGSRRLYGSGVSCHSYLGGYCGPC

>Ps_Beta-71
MAFSSLCYPECGVARPSPVSGTCNEPCVRQCPDSEVVIRPSPVVVTLPGPVLSTFPQQSEVAAVGEPVVGAGYGGSFANGGLY
GYGGRYGGLFGYGGYGYGGRYGYGGLWGYPGFYGNGGYWGYPGRYAHGGYCGYPGFYGNGGFWGYPGRYAHGGYCGYPGYYGN
WGYPGYYGGLCGGYGGYGRRYLGGYCGPC

>Ps_Beta-72_partial
MALSSLCYPECGVARPSPVSGTCNEPCVRQCPDSEVVIRPSPVVVTLPGPVLSTFPQQSEVAAVGEPVVGAGYGGSFANGGLY
GYGGRYGGLFGYGGYGYGGRYGYGGLWGYPGFYGNGGYWGYPGFFGNRGDWGYPRXXXXXXXXXX

>Ps_Beta-73
MAFSSLCYPECGVARPSPVSGTCNEPCVRQCPDSEVVIRPSPVVVTLPGPVLSTFPQQSEVAAVGEPVVGAGYGGSFANGGLY
GYGGRYGGLFGYGGYGYGGRYGYGGYPGFYGYGGYCGYPGYYGNGGFWGYPGRYAHGGYCGYPGYYGNGGYCGYPGYYGNGGY
CGYPGYYGYGGVVGSGVSCHRYRSGNCAPC

>Ps_Beta-74
MSCYPECGVARPSPVSGTFNEPCVRQCPDSEVVIRPSPIAVTIPGPIMSTFPQHSEVGAVGAPLVGSGYGGSFGAGGLFGSGA
GYGGLFGLGGLGGYGGHFGYGGLCGYGGRYGYGGGYGYGGSCGTGVSCHRYLSACGSPC

>Ps_Beta-75_partial
XXXXXXXXXXIRFPGPTLATFAQDSVVGSSAPWLPYGPYGPSRGGAGGGAGSGAGGGVYGGVFGGGVYGGALGGGSGSGFGGC
SGGGYGGSFRQWSRYDRKCYANRYDCCPPC

>Ps_Beta-76
MSFAPIIPLTLVLYVESTPLLSSSGLPPSLKTSFNGEPCTDRCHNPYEVTCPQPIGNSSNQPCVVACGDSRVVVCPPLVVVTC
PGPILSTCPQDSIVGSSIPSGSRIFGSTSMISWHQESLGTQSHMQGESS

Supplementary Figure S2. Amino acid sequences of EDC genes of *Chelonia mydas* (Cm) and *Pelodiscus sinensis* (Ps). (A) Amino acid sequences of EDC proteins of Cm (beta-keratins are not included). (B) Amino acid sequences of EDC proteins of Ps (beta-keratins are not included). (C) Amino acid sequences of beta-keratins of Ps. Amino acid residues C, G, K, P, Q, and S are highlighted by specific colors (see main text and legend of Suppl. Fig. S1).

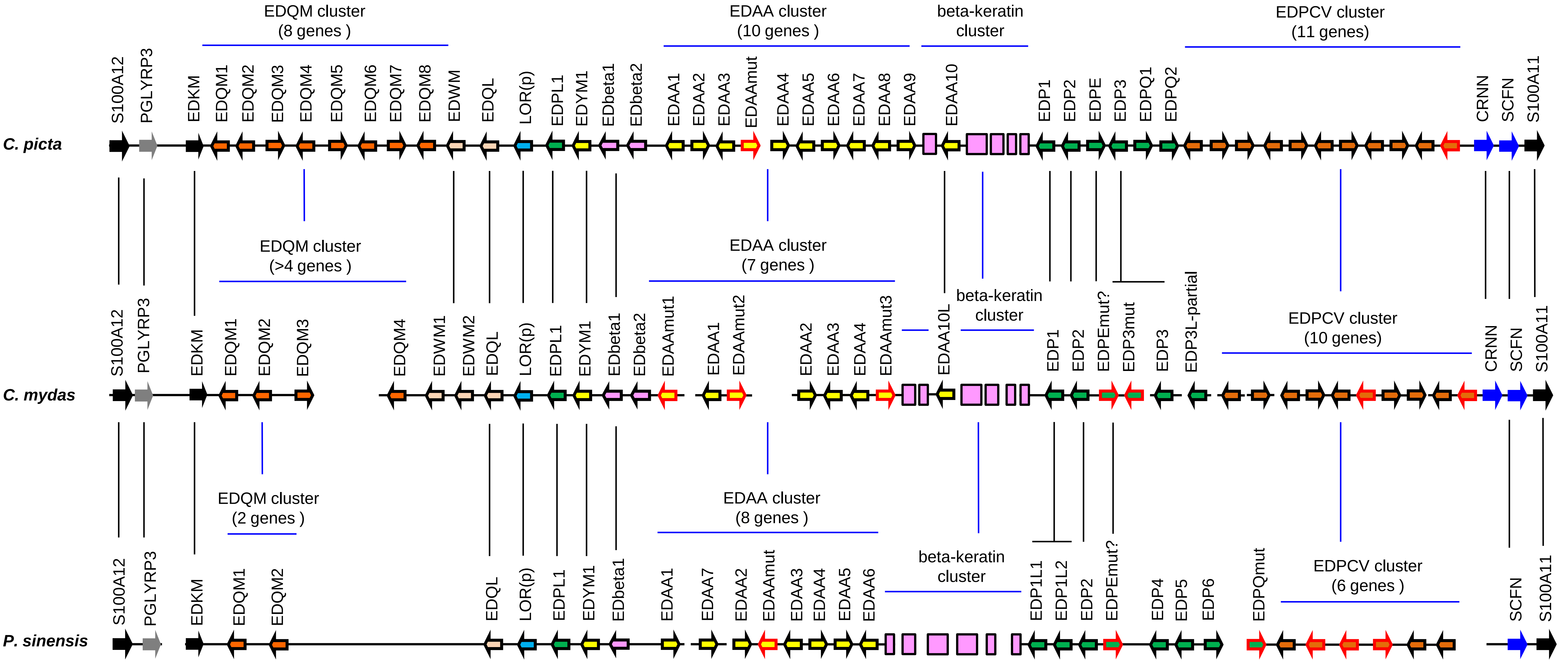

Supplementary Figure S3. Comparison of the EDCs in 3 species of turtles. The arrangement of genes on the EDC of *C. picta*, *C. mydas*, and *P. sinensis* is schematically depicted. Simple EDC (SEDC) genes with 2 exons are represented by colored arrows with a black frame whereas other genes are shown as filled arrows without frame; red frames indicate SEDC genes that are inactivated by mutations. Clusters of more than 2 beta-keratin genes are shown as boxes. Colors indicate groups of genes as defined in the text. Black vertical lines connect orthologs. Note that the schemes are not drawn to scale. The complete sequences of SEDC genes containing multiple internal repeats, such as LOR and EDPE, could not be faithfully predicted because of uncertainties in the genomic sequence assembly (p, partial sequence available; mut, mutation inactivating the gene). Note that the EDC of the soft-shelled turtle (*P. sinensis*) lacks the genes EDWM and CRNN and contains fewer functional genes of the families EDQM and EDPCV.

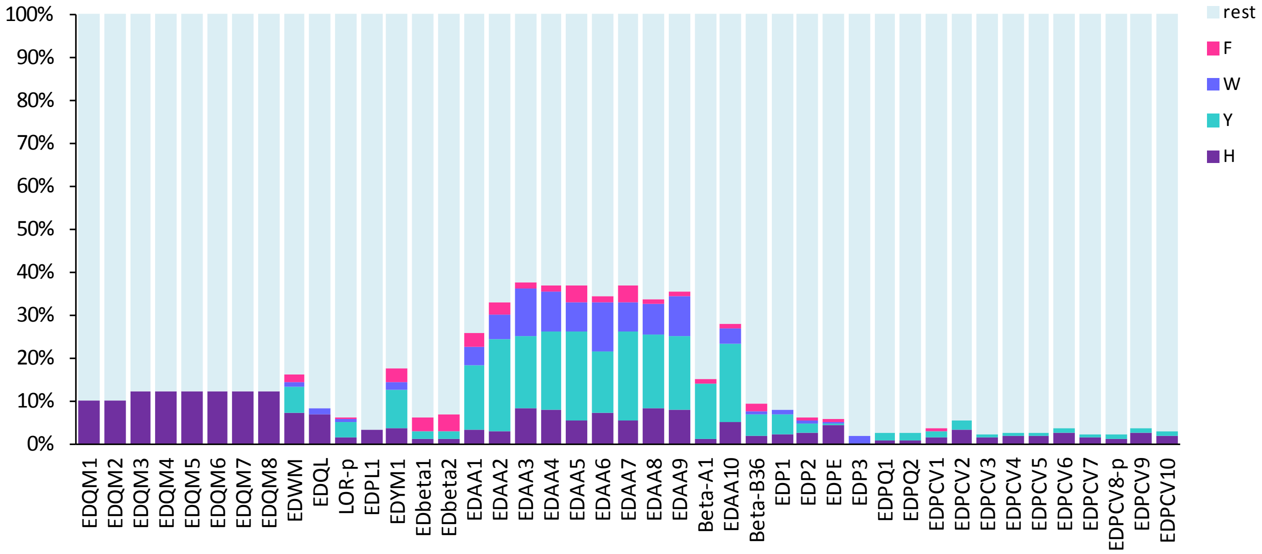

Supplementary Figure S4. Aromatic amino acid contents of turtle SEDC proteins. The contents of aromatic amino acid residues (% of total residues) in *Chrysemys picta* SEDC proteins are depicted for comparison with Figure 3A. Note that only the beta-keratins encoded by the first (Beta-A1) and last (Beta-B36) gene of the beta-keratin cluster are included here.

A

```
>Cp_CRNN
MTQLLSNIKGIINAFYVFAKKDGACPTLSKGELRQLIHQEFADVTVVPQGLQTIDKLLQLLDTDSDGRLDFNGFLVLIFQVAK
ACYGEVSQGQRPGHGGSSASQGEANCGERTKEPP
TPERDPSPRQAPEPQ
TPERDSIPCQAPEPQ
IPEQDSSPCQAPELQ
IPEQDSNPCQAPEAQ
IPERDPSPCQAPELQ
TPEQDSSPRQAPEPQ
IPEQDSSPRQALEPQ
IPEQDSIPCQAPEAQ
IPERDPSPHQAPEPQ
TPERDPSPHQGLEPQ
TPERDPSPCHAPEPQ
IPEQDPSLHQAPEPQ
TPEQDSSSRHTPEPQ
IPEQEPSPCRGPEPQ
TPEQDLSPSQAPEPQ
TPEQDLSHSETQLPP
TQQRNQGAQDPTEPAAGQASKSSQCLYSWHSQKPRPFPHWWPPKK
```

B

```
>Cm_SCFN_partial
MPQLLDSISTIISVFYKHGKKDEDCSTISKREVKRFIQREFDNITVNPYDAHTIEAVLQLLDRDGDGAVDFNEFLLLVFRVAK
VCYWYLQPKQHLPQRTETGLSGERRQEPEAGRAEGSRDQ
PEPPGTEKGYYETRESETREIERSCRQPREPEPRGDQGGHYEIRE
PKPRENERSRHQLGEPEPRGDERRRYETREPEPREEERRHPQPRE
PEPRGNERSHREPFE-----------QPREPEPRGESPXXXXPHE
PEPREDESRRRQPREPEPREDERSHRQPREPQPREEERSRRQPHE
PEPREDESRRRQPREPEPREDERSHRQPREPQPREEERSRRQPHE
PEPREDESRRRQPREPEPREDERSHRQPREPQPREEERSRRQPHE
PEPREDESRRRQPREPEPREDERSHRQPREPQPREEERSRRQPHE
PEPREDESRRRQPREPEPREDERSHRQPREPQPREEERSRRQPHE
PEPREDESRRRQPREPEPREDERSHRQPREPQPREEERSRRQPHE
PEPREDESRRRQPREPEPREDERSHRQPREPQPREEERSRRQPHE
PEPREDESRRRQPREPEPREDERSHRQPREPQPREEERSRRQPHE
PEPREDESRRRQPREPEPREDERSHRQPREPQPREEERSRRQPHE
PEPREDESRRRQPREPEPREDERSHRQPREPQPREEERSRRQPHE
PEPREDESRRRQPREPEPREDERSHRQPREPQPREEERSRRQPHE
PEPREDESRRRQPREPEPREDERSHRQPREPQPREEERSRRQPHE
PEPREDESRRRQPREPEPREDERSHRQPREPQPREEERSRRQPHE
PEPREDESRRRQPREPEPREDERSHRQPREPQPREEERSRRQPHE
PEPREDESRRRQPREPEPREDERSHRQPREPQPREEERSRRQPHE
PEPREDESRRRQPREPEPREDERSHRQPREPQPREEERSRRQPHE
PEPREDESRRRQPREPEPREDERSHRQPREPQPREEERSRRQPHE
PEPREDESRRRQPREPEPREDERSHRQPREPQPREEERSRRQPHE
PEPREDESRRRQPREPEPREDERSHRQPREPQPREEERSRRQPHE
PEPREDESRRRQPREPEPREDERSHRQPREPQPREEERSRRQPHE
PEPREDERSRRQPREPEPREEERSRHQPQEPESREDEGSCCHPRK
PEPREDGGSHLQPPEPESREDERSHHQPREPESQEDESSLHQPRE
PEPREDERSRRQPNEPERREGERCSIQPQTPEPPHEEGYLPPSSE
PEAREDLGTLHEPNDPESTDDYRKFPATHEPSPTGDEGSQLQPRE
SALPGDEGSPQKRRESEPREDDGSQPQLCEPEHREGDGVLRHTPE
SQPLEEAGSQEQPHDLEPRTEGNRHQLPQGGEASHVQRDAEPQQGNGSRHQPREPQEESERSSHQAREPLPQLGEEPQGEWSQ
SHPEPANAEEEPGETDPDEAKASLPCNPLYVYLLEQTVEKQLYLAPPCQKRP
```

Supplementary Figure S5. S100 fused-type proteins (SFTPs) of turtles contain sequence repeats. The amino acid sequences of 2 exemplary SFTPs (CRNN, cornulin (A); SCFN, scaffoldin (B)) of turtles (Cm, *C. mydas*; Cp, *C. picta bellii*) are shown. Sequence repeat elements are aligned. For comparison with other EDC proteins, amino acid residues C, G, K, P, Q, and S are highlighted with the same colors as in the amino acid sequences shown in Supplementary Figures S1 and S2. The sequence repeats of CRNN are not perfect. Sequence repeat elements of SCFN are composed of 3 internal imperfect repeats with the consensus sequence P(E/Q)PRE(D/E)E(R/S)(S/R)(R/H)RQP(R/H)E. Sequence repeats differ among turtle species (not shown). X, unknown residues because of incomplete gene sequence.

A

```
Hs Lor      MSY-----QKKQPTPQPP
Hs PRR9     MSFS--EQQCKQPCVPPP
Hs SPRR1A   MNS---QQQ-KQPCTPPP
Hs SPRR1B   MSS---QQQ-KQPCTPPP
Hs SPRR2A   MSY--QQQQCKQPCQPPP
Hs SPRR2B   MSY--QQQQCKQPCQPPP
Hs SPRR2D   MSY--QQQQCKQPCQPPP
Hs SPRR2E   MSY--QQQQCKQPCQPPP
Hs SPRR2F   MSY--QQQQCKQPCQPPP
Hs SPRR2G   MSY--QQQQCKQPCQPPP
Hs SPRR4    MSS--QQQQRQQQQCPPQ
Ac EDCP     MSY-----QCKQRCLPPP
Ac EDPQ2    MSY--QQQQCKQPCQPPP
Ac EDPQ3    MSSDSF--QCTQPCKAPP
Ac EDSPR2   MS-----QQCKQGCKAPP
Ac EDSQ     MSY-----QVKQASLPPP
Ac EDEPT    MSY-----QARQPCTAPP
Ac EDSPR1   MACPH--QQCKQPCLPPP
Ac EDPSQ    MYC--TDQQCKQACLPPP
Gg EDCQCM   MSYY---EQCKQPCLPPP
Gg EDPE     M-------QCKQEVTLPP
Gg EDYM1    MSYWY---QYKQQCFIPS
Cp EDYM1    MSYFAY--QYKQRNYTPY
Cp EDAA1    MFH--HQKICKPWCCKPH
Cp EDP3     MSS--DQQQCKQTCPPPP
Cp EDPCV1   MAY---QQQCKQPCLPPP
Cp EDPCV2   MAY---QQQCKQTCLPPP
Cp EDPCV3   MAY---QQQCKQTCLPPP
Cp EDPCV4   MAY---QQQCKQTCLPPP
Cp EDPCV5   MAY---QQQCKQTCLPPP
Cp EDPCV6   MAY---QQQCKQTCLPPP
Cp EDPCV7   MAY---QQQCKQTCLPPP
Cp EDPCV8   MAY---QQQCKQTCLPPP
Cp EDPCV9   MAY---QQQCKQTCLPPP
Cp EDPCV10  MAY---QQQCKQPCLPPP
Cp EDPE     MSLHQDQQQCKQGITLPP
Cp EDPQ1    MSY-QHQQQCKQTCLPPP
Cp EDPQ2    MSY-QHQQQCKQTCLPPP
Cp EDPL1    MSCHQHQQQCKQPCMPPP
```

B

```
PGLYRPs   Hs PGLYRP3  ALYNIISTWPHF------KH-*
          Ac PGLYRP3  PIRAEISKWPNY------KHN*
          Cp PGLYRP3  PIRKVLKTWPHY------KH-*
SFTPs     Gg Crnn     QEHLQP-QWPP--------RK*
          Cp Crnn     KPRPFPHWWPP--------KK*
SEDCs     Hs Ivl      QQ-KQEVQWPP-------KHK*
          Hs Lor      QQ-KQAPTWPS---------K*
          Ac EDCQ1    QQVKQPTQWPS------QNQK*
          Ac EDCQ2    QQVKQPTQWPP------QNAK*
          Ac EDEPK    QQRKQPSTWPL---------K*
          Ac EDPKC    HQKKQPCYWPH--------HK*
          Ac Lor1     Q-TKQMNTWPSG-------QK*
          Gg EDGH     QQIKQSSQWPPS------QKK*
          Gg EDPE     QQVKQPSPWPLT-------QK*
          Gg EDQrep   KKYCSASKWPW----------*
          Gg EDQL     QQIKQPVQWPT------QQQK*
          Gg Lor1     QQT-QPISWPPQT-----KHK*
          Cp Lor      QQTKQPCQWPPNP------RK*
          Cp EDQL     QQTKQPCQWPPQ-----KHQK*
          Cp EDAA5    HGYGYGKFWPCFA----EEQ-*
          Cp EDP2     QQQKQPHHWPP-------KRK*
          Cp EDYM1    YPYPYAPQWPNTWGYGNCGPC*
```

Supplementary Figure S6. Conserved amino acid sequence motifs at the amino-terminus (A) and carboxy-terminus (B) of EDC proteins. The amino acid sequence motifs of the lizard (*Anolis carolinensis*, Ac), chicken (*Gallus gallus*, Gg) and human (*Homo sapiens*, Hs) were discussed in detail in Strasser et al. (2014). The present study shows that these motifs are also conserved in several but not all EDC proteins of the turtle (*Chrysemys picta*, Cp). *, end of the protein.

```
              1                                                                                                      106
Cp_EDQM1  MCSRQEKDHCHKQDTCHGSGG----GSSCHGSGGGSSCHGSGGGSSCHGSGGGSSCHGSGGGSSCHGSGG----GSSCHGKPQKPCQQEQQQQQKHCCQVPSQKLK
Cp_EDQM2  MCSRQEKDHCHKQD---VSGGCHSSGSSCH-SSGGSSCHS--GGSSCHGSGG-SSCHSSGG-SSCHGGGSSCHSGGSCHGKPQQPCQQ--QQQQKIC-KVPCQKLK
Cp_EDQM3  MCSRQEKDHCHKQDGCHSSGGCHSSGSSCH-SGGGSSCHS--GGSSCHGSGG-SSCHSSGG-SSCHGGGSSCHSGGSCHGKPQQHCQQ--QQQQKIC-KVPCQKLK
Cp_EDQM4  MCSRQEKDHCHKQDGCHSSGGCHSSGSSCH-SGGGSSCHS--GGSSCHGSGG-SSCHSSGG-SSCHGGGSSCHSGGSCHGKPQQHCQQ-QQQQQKIC-KVPCQKLK
Cp_EDQM5  MCSRQEKDHCHKQDGCHSSGGCHSSGSSCH-SSGGSSCHS--GGSSCHGSGG-SSCHSSGG-SSCHGGGSSCHSGGSCHGKPQQHCQQ--QQQQKIC-KVPCQKRK
Cp_EDQM6  MCSRQEKDHCHKQDGCHSSGGCHSSRSSCH-ESGGSSCHS--GGSSCHGSGG-SSCHSSGG-SSCHGGGSSCHSGGSCHGKPQQHCQQ--QQQQKIC-KVPCQKLK
Cp_EDQM7  MCSRQEKDHCHKQDGCHSSGGCHSSGSSCH-SSGGSSCHS--GGSSCHGSGG-SSCHSSGG-SSCHGGGSSCHSGGSCHGKPQQHCQQ--QQQQKIC-KVPCQKLK
Cp_EDQM8  MCSRQEKDHCHKQDGCHSSGGCHSSGSSCH-SSGGSSCHS--GGSSCHGSGG-SSCHSSGG-SSCHGGGSSCHSGGSCHGKPQQHCQQ--QQQQKIC-KVPCQKLK
```

Supplementary Figure S7. Amino acid sequence alignment of epidermal differentiation proteins containing a glutamine (Q)-rich motif (EDQMs) of *C. picta bellii* (Cp). Amino acid residues implicated in covalent protein cross-linking (C-C, Q-K) are highlighted.

```
            1                                                                                           94
 Cp_EDAA1   M-FHHQKICKPWCCKPHQKICKPWCCKPWGYGGSSGYGGDYGYCPPFWCKKPFKCCYPYPYPGCYPYPKPCCYPCPYPYPCGPGYQYPCLAEEE
 Cp_EDAA2   MSFN-KSIIGELYYNPC------------CYGGYRGYRG-YGYCRPWCYQRPYKYGWGHHYKCCYPYPYQWGYGK----------GWPCFAEEE
 Cp_EDAA3   MNYHHQKLSHHWGCDPC------------WNGGWGGYGGHYGCYRPWGYYRPYGWGWGHSYG----YPYRWGGGYGYGR------CWPCFAEEE
 Cp_EDAA4   MTYHHQKLSHHWGCDPC------------WNGGWGGYGGHYGCYRPWGYYRPYSYGWGHNYGSCYSYPYRWGGGYGYGR------CWPCFAEEQ
 Cp_EDAA5   MTFD-ELMNEELYYNPY------------CYKGWRGYRGHYGCYRPWGYQRPYRYGWGHQYDCHYPY--RWGHGYGYGK------FWPCFAEEQ
 Cp_EDAA6   MTFD-ESINDELYYNPW------------SHGCWHGSRGHYGCGRPWGYGRQSRWGWGHGYDCYYPYSSRWGHWYPYVK------QWPC-----
 Cp_EDAA7   MTFD-ELMNEELYYNPY------------CYKGWRGYRGHYGCYRPWGYQRPYRYGWGHQYDCHYPY--RWGHGYGYGK------FWPCFAEEQ
 Cp_EDAA8   MTFHHQKLSHHWGCDPCSSGSWGGYRG--HYDCYRPWGYSRPYGCGWGYNDGCYYPYSSRWGHGYGGYGYGGCGYGYGG-HGYGKCWPC-----
 Cp_EDAA9   MNYHHQKLSHHWGCDPC------------WNGGWGGYGGHYGCYRPWGYYRPYSYGWGHNSGSCYSYPYRWGGGYGYGR------CWPCFAEEQ
Cp_EDAA10   MTWSGYGYNDGCYSPCGYGGRWAYGSPCGYRGL-CGYGGHSSHGGSWGYRGSYGYRGAYHSGYCYPFSSQQGHRYSYGN------CGPC-----
Cp_EDAA11   MTYHHQKLSHHWGCDPC------------WNGGWGGYGGHYGCYRPWGYYRPYSYGWGHNSGSCYSYPYRWGGGYGYGR------CWPCFAEEQ
Cp_EDAA12   MNYHHQKLSHHWGCDPC------------WNGGWGGYGGYYGCYRPWGYYRPYSYGWGHNSGSCYSYPYRWGGGYGYGR------CWPCFAEEQ
Cp_EDAA13   MTYHHQKLSHHWGCDPC------------WNGGWGGYGGHYGCYRPWGYYRPYSYGWGHNYGSCYSYPYRWGGGYGYGR------CWPCFAEEQ
Cp_EDAA14   MTYHHQKLSHHWGCDPC------------WNGGWGGYGGHYGCYRPWGYYRPYSYGWGHNYGSCYSYPYRWGGGYGYGR------CWPCFAEEQ
Cp_EDAA15   MNYHHQKLSHHWGCDPC------------WNGGWGGYGGHYGCYRPWGYYRPYSYGWGHNSGSCYSYPYRWGGGYGYGR------CWPCFAEEE
Cp_EDAA16   MNYHHQKLSHHWGCDPC------------WNGGWGGYGGHYGCYRPWGYYRPYSYGWGHNYGSCYSYPYRWGGGYGYGR------CWPCFAEEE
Cp_EDAA17   MTFD-ELMNEELYYNPY------------CYKGWRGYRGHYGCYRPWGYQRPYRYGWGHQYDCHYPY--RWGHGYGYGK------FWPCFAEEQ
Cp_EDAA18   MTFD-ELMNEELYYNPY------------CYKGWRGYRGHYGCYRPWGYQRPYRYGWGHQYDCHYPY--RWGHGYGYGK------FWPCFAEEQ
Cp_EDAA19   MTFD-ENFSDELYYKPY------------HYGGWGG-RG-YGYCRPWCYQRPYKCCWGYPKGCWYPDPCHWGWGYGYGK------GWPCFAQEE
Cp_EDAA20   MTFD-ENFSEKLDYKPC------------HYGGWRG-RG-YGWGRPWCYQRPYRCCWGYPKGCWYPYPCHWGWGYGYGK------GWPCFAQEE
Cp_EDAA21   MTFD-ENFSDELYYKPY------------HYGGWGG-RG-YGYCKPWCHQRPYKCCWGYPKGCWYPYPCHWGWGYGYGK------DWPCFAQEE
Cp_EDAA22   MTFD-ENFSDELYYKPY------------HYGGWGG-RG-YGWCRPWCYQRPYKCCWGYPKGCWYPYPCHWGWGYGYGK------GWPCFAQEE
```

Supplementary Figure S8. Amino acid sequence alignment of epidermal differentiation proteins rich in aromatic amino acids (EDAAs) of *C. picta bellii* (Cp). Aromatic amino acids are highlighted.

```
              1                                                                                        90
Cp_EDPCV1     MAYQQQCKQPCLPPPCCVKQCKTKCVDPC--PCPPQCVDPCPPCPPKCVDPCPPKCVDPCPPKCVDLCPPKCVDQFPCPPKCVDVCPP--
Cp_EDPCV2     MAYQQQCKQTCLPPPCCVTKCVTKCLDPCYKVCVTKCVTKCLDPCCKVCVKKCTRCVHPCSCP---------------------------
Cp_EDPCV3     MAYQQQCKQTCLPPPCCVTKCTTKCLDPCCKVCVTKCVTKCVDPCCKVCVKKCTTCVHPCPCPCP--QKCLPCPPCPQKCPPCPPCPPCP
Cp_EDPCV4     MAYQQQCKQTCLPPPCCVTKCTTKCLDPCCKVCVTKCVTKCVDPCCKVCVKKCTTCVHPCPCPCP--QKCIPC---PQKCI---PCPPCP
Cp_EDPCV5     MAYQQQCKQTCLPPPCCVTKCTTKCLDPCCKVCVTKCVTKCVDPCCKVCVKKCTTCVHPCPCPCP--QKCLPC-------------PPCP
Cp_EDPCV6     MAYQQQCKQTCLPPPCCVTKCTTKCLDPCCKVCVTKCVTKCVDPCCKVCVKKCTTCVHPCPCPCP--QKCLPC----------PPCPPCH
Cp_EDPCV7     MAYQQQCKQTCLPPPCCVTKCTTKCLDPCCKVCVTKCVTKCVDPCCKVCVKKCTKCVHPCPCPCP--QKCIPCPPCPQKCPPCPPCPPCP
Cp_EDPCV8     MAYQQQCKQTCLPPPCCVTKCTTKCLDPCCKVCVTKCVTKCVDPCCKVCVKKCTTCVHPCPCPCP--QKCLPC-------------PPCP
Cp_EDPCV9     MAYQQQCKQTCLPPPCCVTKCTTKCLDPCCKVCVTKCVTKCVDPCCKVCVKKCTTCVHPCPCPCP--QKCPPC----------APCPPCP
Cp_EDPCV10    MAYQQQCKQPCLPPPCCVTKCTTKCLDPCCKVCV----------------KKCTTCVHPCPCPCPCPQKCLPC----------PPCPPCP
Cp_EDPCV11    MAYQQQCKQTCLPPPCCVTKCTTKCLDPCCKVCVTKCVTKCVDPCCKVCVKKCTTCVHPCPCP----QKCIPCPP-------------CP
Cp_EDPCV12    MAYQQQCKQTCLPPPCCVTKCTTKCLDPCCKVCVTKCVTKCVDPCCNVCVKKCTTCVHPCPCPCP--QKCIPCPP-------------CP
Cp_EDPCV13    MAYQQQCKQTCLPPPCCVTKCTTKCLDPCCKVCVTKCVTKCVDPCCKVCVKKCTTCVHPCPCPCP--QKCIPCPPCPQKCPPCPQK//CP
Cp_EDPCV14    MAYQQQCKQTCLPPPCCVTKCTTKCLDPCCKVCVTKCVTKCVDPCCKVCVKKCTTCVHPCPCPCP--QKCLPCP---QKCL---PCPPCP
Cp_EDPCV15    MAYQQQCKQTCLPPPCCVTKCTTKCLDPCCKVCVTKCVTKCVDPCCKVCVKKCTTCVHPCPCPCP--QKCLPCPPCPQKCPPCPPCPPCP

              91                                                                 158
Cp_EDPCV1     -KCVDL-----CPPKCVD--QCPCPPKCVDVCPPKCVDV-CPP---KCVDVCPPCPPLQHCCQEKKHY
Cp_EDPCV2     ---------------------------------PKHVD--------PCPPCLPKCPPVQHCCKEKKPC
Cp_EDPCV3     QKCPPC------PQKCPPCPPCPPCPQKCPPCPPCPQK--CPP-CPPCPP---KCSPVQHCCKEKKLC
Cp_EDPCV4     QKCPPC---PPCPQKCPPC--------------------------PPCPPCLPKCPPVQHCCKEKKLC
Cp_EDPCV5     QKCPPC---PPCPQKC-----------------------------PPCPPCLPKCPPVQHCCKEKKLC
Cp_EDPCV6     QKCPPC------PQKCPPC--------------------------PPCPPCPPKCPPVQHCCKEKKLC
Cp_EDPCV7     QKCPPCPPCPPCPQKCPPCL---PCPQKCPPC-------------PPCPPCLPKCPPVQHCCKEKKLC
Cp_EDPCV8     QKCPPCPPCPPCPQXXXXXXXXXXXXXXXXXXXXXXXXXXXXXXXXXXXXXXXXXXXXXXXXXXXXXX
Cp_EDPCV9     QKCPPCPPCPPCHQKC-------------------PPCPPCLP----------KCPPVQHCCKEKKLC
Cp_EDPCV10    QKCPPCPPCPPCPEKCPPC----------------PPCPPC----------PPECPPVQHCCKEKKLC
Cp_EDPCV11    QKCPPC---PPCPQKCPPC----------------PPCPPCLP----------KCPPVQHCCKEKKLC
Cp_EDPCV12    QKCPPC----------------PPCPQKCPPC---PPCPPCLP----------KCPPVQHCCKEKKLC
Cp_EDPCV13    QKCPPCPPCPPCPQKCPPCPPCPPCPQKCPPCPPCPPCLPCPQKCPPCPP---KCPPVQHCCKEKKLC
Cp_EDPCV14    QKCPPCPPCPPCPQKCPPCLPCPPCPQKCPPCPPCPPCLPCPQKCPPCPPCPPKCPPVQHCCKEKKLC
Cp_EDPCV15    QKCPPCPPCPPC--------------------------LPCPQKCPPCPPCPPKCPPVQHCCKEKKLC
```

Supplementary Figure S9. Amino acid sequence alignment of epidermal differentiation proteins rich in proline, cysteine and valine (EDPCV) of *C. picta bellii* (Cp). Proline and amino acids implicated in covalent protein cross-linking (C-C, Q-K) are highlighted. At the position indicated by "//" a part of the amino acid sequence of EDPCV13 has been removed to facilitate alignment of the carboxy-terminal sequences. X, unknown residues because of incomplete gene sequence.

```
                        1                                                                                                                                                                                           207
EDbeta      Cp_EDbeta1  MSCGANLC-IDGGSACGVARPRPCADSCNQPCVTQCPDSRVIIYPPPVVVTFPGPILTTFPQESVVESVGAPVV--ASGYGGTSGSGA-------FGVGHGN---------------------------------------------------------------------------------------CGPCGPC---
            Cp_EDbeta2  MSCSRNVC-TAGGSACGVARPRPFTDSCNQPCVTRCPDSRVIIYPPPVVVTFPGPILTTFPQESVVESVGAPVV--ASGYGGTSGSGA-------FGVGHGN---------------------------------------------------------------------------------------RDLCGPC---

Beta A       Cp_Beta-A1  MSCS-SLC-YP---ECGVARPSPVSGSCNEPCVRQCPDSEVIIRPSPVVVTIPGPILSNFPQQSEVGAVGAPVV--GAGYGGSFGLGGLY----GYGGHYGGLYGYGGLGGYGGRYGYGGL----SGYGGLCGYGGRYGYGGLCGYGGRYGYGGLSGYGG----LCGYG---G--GYGGGYGYGGACGSGVSCHRYLSGSCTPC---
             Cp_Beta-A2  MSCS-SLC-YP---ECGVARPSPVSGSCNEPCVRQCPDSEVIIRPSPVVVTIPGPILSNFPQQSEVGAVGAPVV--GAGYGGSFGLGGLY----GYGGHYGGLYGYGGLGGYGGRYGYGG------GYGGLCGYGGRYGYGGLSGYGGRYG--------G----LCGYG---G--GYGGGYGYGGACGSGVSCHRYLSGSCTPC---
             Cp_Beta-A3  MSCS-SLC-YP---ECGVARTSPDSGSCNELCVRQCPDSEVIIRPSPVVVTIPGPILSNFPY--------------RGHYGRLYCYGGLG----GYGGHYG----YGGLCGYRGYGGL----CGYRGRYGYGGLSGYGG--------------------------------------------------HYGGLCD-----
             Cp_Beta-A4  MSCS-SLC-YP---ECGVARPSPVSGSCNEPCVRQCPDSEVIIRPSPVVVTIPGPILSNFPQQSEVGAVGAPVV--GAGYGGSFGLGGLY----GYGGHYGGLYGYGGLGGYGGRYGYGG------GYGGLCGYGGRYGYGGLSGYGG---------------LCGYG---G--GYGGGYGYGGACGSGVSCHRYLSGSCTPC---
             Cp_Beta-A5  MSCS-SLC-YP---ECGVARPSPVSGSCNEPCVRQCPDSEVIIRPSPVVVTIPGPILSNFPQQSEVGAVGAPVV--GAGYGGSFGLGGLY----GYGGHYGGLYGYGGLGGYGGHYGYGGL----SGYGGLCGYGGRYGYGGLSGYGGRYG--------G----LCGYG---G--GYG----YGGACGSGVSCHRYLSGSCTPC---
             Cp_Beta-A6  MSCS-SLC-YP---ECGVARPSPVSGSCNEPCVRQCPDSEVIIRPSPVVVTIPGPILSNFPQQSEVGAVGAPVV--GAGYGGSFGLGGLY----GYGGHYGGLYGYGGLGGYGGRYGYGG--GYGGGYGGLCGYGGRYGYGGLSGYGGRYG--------G----LCGYG---G--GYGGGYGYGGACGSGVSCHRYLSGSCTPC---
             Cp_Beta-A7  MSCS-SLC-YP---ECGVARPSPVSGSCNEPCVRQCPDSEVIIRPSPVVVTIPGPILSNFPQQSEVGAVGAPVV--GAGYGGSFGLGGLY----GYGGHYGGLYGYGGLGGYGGHYGYGGLSGYGGGYGGLCGYGGRYGYGGLSGYGGRYG--------G----LCGYG---G--GYGGGYGYGGACGSGVSCHRYLSGSCTPC---
             Cp_Beta-A8  MSCS-SRC-YP---ECGVARPSPVSGSCNELCVRQCPDSEVIIRPSPVVVTIPGQILSNFPY--------------GGHYGRLYGYGGLG----GYGGHYG----YGGLCGYGGRYGYGGL---------------SGYGG--------------------------------------------------HYGGLCD-----
             Cp_Beta-A9  MSCS-SLC-YP---ECGVARPSPVSGSCNEPCVRQCPDSEVIIRPSPVVVTIPGPILSNFPQQSEVGAVGAPVV--GAGYGGSFGFGGLN----GYGGHYGGLYGYGR--GYGGLSGYGG------GYGGLCGYGGHYGYGGLSGYGGRYG--------G----LCGYG---G--GYG----YGGACGSGVSCHRYLSGSCTPC---
            Cp_Beta-A10  MSCS-SMC-YP---ECGVTRPSPVSGSCNEPCVRQCPDSEVIIRPSPVVVTIPGPILSNFPQQSRVGAIGAPVV--GPGYGASFSLGGLY----GSGSCYGGLYSYGILYGYGGLGGYRG----------------WLWG------------------------------------------------------IRSLWGIM---
           Cp_Beta-A1L1  MSCS-SLS-YP---ECGVARPSPVSGTCNEPCIRQCPDSEVVIRPSPVVVTLPGPILSTFPQQSEVAAVGAPVV--GAGYGGSFGLGGLY----GSGGRYGGLYGLGGFGGYGGLYGYGGL----GGYGGLCGYGGYGYGGLGGYGGLGRYGGLCGYGGGYGGLCGYG---GGYGYGGLGGYGGLCGYGGYGRRYRGGYCGPC---
           Cp_Beta-A1L2  MSCS-SLS-YP---ECGVARPSPVSGSCNEPCVRQCPDSEVVIRPSPVVVTLPGPILSTFPQQSGVGAVGAPVV--GAGYGGSFGLGGLY----GSGGHYGGLYGLGGLGGYGSHYGYGGL----GGYGGLCGYGGYGYGGLGGYGGLGRYGGLCGYGGGYGGLCGYG---GGYGYGGLGGYGGLCGYGGYGRRYRGGYCGPC---

Beta O       Cp_Beta-O1  MTFS-SLC-YP---ECGVARPSPVTGSSNEPCVRQCPDSEVVIRPSPVVVTLPGPILSNFPQQSEVAAVGAPVV--GAGFGGSFGLGGLY----GYGGHYGGLYGLGRLGGYGGRYGYGGL----LGYGGHCGYPGLYGYGGL-------------------------------------------WGYGGYGRRYLSGYCGPC---
             Cp_Beta-O2  MTFS-SLC-YP---ECGVARPSPVTGTCNEPCVRQCQDSEVVIRPSPVVVTLPGPILSNFPQQSEVAAVGAPVV--GAGFGGSFGLGGLY----GYGGHYGGLYGLGRLGGYGGHYGYGGL----LGHGGYCGYPGLYGYGGL-------------------------------------------WGYGGYGRRYLGGYCGPC---
             Cp_Beta-O3  MTFS-SLC-YP---ECGVARPSPVTGSANEPCVRQCPDSEVVIRPSPVVVTLPGPILSNFPQQSEVAAVGAPVV--GAGFGGSYGLGGLY----GYGGHYGGLYGLGRLGGYGGRYGYGGL----LGNGGYCGYPGLYGYGRLWGNGGHCGYPGLYGYGG----LWGYG---GHCGYPGLYGYGGLSGSGVSNHRYLSGSSGPC---
             Cp_Beta-O4  MTFS-SLC-YP---ECGVARPSPITGSSNEPCVRQCPDSEVVIRPSPVVVTLPGPILSNFPQESEVAAVGAPVV--GAGFGGSYGLGGLY----GYGGHYGGLYGLGRLGGYGGHYGYGGL----LGYGGHCGYPGLYGYGGL-------------------------------------------WGYGGYGRRYLGGYCGPC---
             Cp_Beta-O5  MTFS-SLC-YP---ECGVARPSPVTGSSNEPCVRQCPDSEVVIRPSPVVVTLPGPILSNFPQESEVAAVGAPVV--GAGFGGSFGLGGLY----GYGGHYGGLYGLGRLGGYRGLYGYGRL----LGHGGYCGYPGLYGYGGL-------------------------------------------WGYGGYGRRYLGGYCGPC---
             Cp_Beta-O6  MTFS-SLC-YP---ECGVARPSPVTGSCNEPCVRQCQDSQVVIRPSPVVVTLPGPIMSNFPQHSAVGAVGAPVV--GAGFGGSYGLGGLN----GSGGQYGGLSGLGGYGGYGGL----------------------------------------------------------------CGSGVSCHRYLSGSGGLC---
             Cp_Beta-O7  MTFS-SLC-YP---ECGVARPSPVTGSCNEPCVRQCQDSQVVIRPSPVVVTLPGPIMSNFPQHSAVGTVGAPVV--GAGFGGSYGLGGLN----GSGGQYGGLSGLGGYGGYGGL----------------------------------------------------------------CGSGVSCHRYLSGSCGPC---
             Cp_Beta-O8  MTFS-SLC-YP---ECGVAQPSPVTGSCNEPCVRQCPDSQVVIRPSPVVMTLPGPILSNFPQHSVVGAVGAPVV--GAGFGGSYGLGGLN----GSGGHYGGWSGLGGYGGYGGL----------------------------------------------------------------SGSGVSYHRYLSGSCGPC---
             Cp_Beta-O9  MTFS-SLC-YP---ECGVARPSPVTGTCNEPCVRQCQDSEVVIRPSPVVVTLPGPIMSNFPQHSGVGAIGAPVV--GPGFGGSFGHGG-F----GYGGLYGGLHGLGGYGGYGGHYGYGGL----GGY------LGGYGYGGL-------------------------------------------CGSGVSCHRYLSGNCGPC---
            Cp_Beta-O10  MTFS-SLC-YP---ECGVARPSPVTGTCNEPCVRQCQDSEVVIRPSPVVVTLPGPIMSNFPQHSGVGAVGAPVV--GPGFGGSFGHGG-F----GYGGLYGGLHGLGGYGGYGGHYGYAGL----GGY------LGGYGYGGL-------------------------------------------CGSGVSCHRYLSGNCGPC---
            Cp_Beta-O11  MTFS-SLC-YP---ECGVARPSPVTGTCNEPCVRQCQDSEVVIRPSPVVVTLPGPIMSNFPQHSGVGAVGAPVV--GPGFGGSFGHGG-F----GYGGLYGGLYGLGGYGGYGGHYGYGGL----GGY------LGGYGYGGL-------------------------------------------CGSGVSCHRYLSGNCGPC---
            Cp_Beta-O12  MTFS-SLC-YP---ECGVARPSPVTGTCNEPCVRQCQDSEVVIRPSPVVVTLPGPIMSNFPQHSGVGAVGAPVV--GPGFGGSFGHGG-L----GYGGLYGGLYGLGGYGGYGGHYGYGGL----GGY------LGGYGYGGL-------------------------------------------CGSGVSCHRYLSGNCGPC---
            Cp_Beta-O13  MTFS-SLC-YP---ECGVARPSPVTGTCNEPCVRQCQDSEVVIRPSPVVVTLPGPIMSNFPQHSGVGAVGAPVV--GPGFGGSFGHGG-F----GYGGLYGGLYGLGGYGGYGGHYGYAGL----GGY------LGGYGYGGL-------------------------------------------CGSGLSCHRYLSGNCGPC---
            Cp_Beta-O14  MTFS-SLC-YP---ECGVARPSPVTGTCNEPCVRQCQDSEVVIRPSPVVVTLPGPIMSNFPQHSGVGAVGAPVV--GPGFGGSFGRGG-F----GYGGLYGGLYGLGGYGGYGGHYGYGGL----GGY------LGGYGYGGL-------------------------------------------CGSGLSCHRYLSGNCGPC---
            Cp_Beta-O15  MTFS-SLC-YP---ECGVARPSPVTGTCNEPCVRQCQDSEVVIRPSPVVVTLPGPIMSNFPQHSGVGAVGAPVV--GPGFGGSFGHGG-F----GYGGLYGGLYGLGGYGGYGGHYGYGGL----GGYGGLGGYLGGYGYGGL-------------------------------------------CGSGVSCHRYLSGNCGPC---
            Cp_Beta-O16  MTFS-SLC-YP---ECGVARPSPVTGTCNEPCVRQCQDSEVVIRPSPVVVTLPGPIMSNFPQHSGVGAVGAPVV--GPGFGGSFGHGG-F----GYGGLYGGLYGLGGYGGYGGHYGYGGL----GGYGGLGGYLGGYGYGGL-------------------------------------------CGSGVSCHRYLSGNCGPC---
            Cp_Beta-O17  MTFS-SLC-YP---ECGVARPCPVTGTCNEPCVRQCQDSEVVIRPSPVVVTLPGPILSNFPQHSGVGALGAPVV--GPGFGGSFGHGG-F----GYGGLYGGLYGLGGYGGYGGRYGYGGL----WGHGGYCGYPGLYGYGGL-------------------------------------------LGYGGYGRRYLGGRCGPC---
            Cp_Beta-O18  MTFS-SLC-YP---ECGVARPCPVTGTCNEPCVRQCQDSEVVIRPSPVVVTLPGPILSNFPQHSAVGALGAPVV--GPGFGGSFGHGALY----GYGGLYGGWCGLGGYGGYCGPYGYG---------GLGGYLGCYGYGGI-------------------------------------------CGSGVSCHRYLSGSCGPC---
            Cp_Beta-O19  MTFS-SLC-YP---ECGVAQPCPVTGTCNEPCVRQCQDSEVVIRPSPVVVTLPGPIMSHYTQESVVGALGAPVV--GPGFGGSFGHGGLY----GYGGRYSGWYGLGGYGGYCGPYGYGSL----GGYGGLGGYVGGYGYGGI-------------------------------------------CGSGVSCHRYLSGSCGTC---
            Cp_Beta-O20  MTFS-SLC-YP---ECGVARPCPVTGTCNEPCVRQCPDSEVVIRPSPVVVTLPGPILSNFPQHSAVGAVGAPVV--GPGFGGSFGHGG-Y----GYGGLYGGLYGLGGYGGYGGHYGYGGL----WGHGGYCGYPGLYGYGGL-------------------------------------------WGYGGYGRRYLGGHCGAW---
            Cp_Beta-O21  MTFS-SLC-YP---ECGVARPCPVTGTCNEPCVRQCPDSEVVIRPSPVVVTLPGPIMSNFPQHSGVGAVGAPVV--GPGFGGSFGHGG-Y----GYGGLYGGLYGLGGYGGYGGHYGYGGL----LGHGGYCGYPGLYGYGGL-------------------------------------------WGYGGYGRRYLGGRCGTW---
            Cp_Beta-O22  MTFS-SLC-YP---ECGVARPCPVTGTCNEPCVRQCQDSEVVIRPSPVVVTLPGPILSNFPQHSAVGAVGAPVV--GPGFGGSFGHGG-Y----GYGGLYGGLYGLGGYGGYGGHYGYGGL----WGHGGYCGYPGLYGYGGL-------------------------------------------WGYGGYGRRYLGERCGTC---
            Cp_Beta-O23  MTFS-SLC-YP---ECGVARPCPVTGTCNEPCVRQCQDSEVVIRPSPVVVTLPGPIMSNFPQHSGVGAVGAPVV--GPGFGGSFGHGG-F----GYGGLYGGLYGLGGYGGYGGHYGYGGL----WGHGGYCGYPGLYGYGGL-------------------------------------------WGYGGYGRRYLGGRCGTC---
            Cp_Beta-O24  MTFS-SLC-YP---ECGVARPCPVTGTCNEPCVRQCQDSEVVIRPSPVVVTLPGPIMSNFPQHSGVGAVGAPVV--GPGFGGSFGHGG-F----GYGGLYGGLYGLGGYGGYGGHYGYGGL----WGHGGYCGYPGLYGYGGL-------------------------------------------WGYGGYGRRYLGGRCGTC---
            Cp_Beta-O25  MTFS-SLC-YP---ECGVARPCPVTGTCNEPCVRQCQDSEVVIRPSPVVVTLPGPIMSNFPQHSGVGAVGAPVV--GPGFGGSFGHGG-F----GYGGLYGGLYGLGGYGGYGGHCGYGGL----WGHGGYCGYPGLYGYGGL-------------------------------------------WGYGGYGRRYLGGRCGTC---
            Cp_Beta-O26  MTFS-SLC-YP---ECGVARPCPVTGTCNEPCVRQCPDSEVVIRPSPVVVTLPGPIMSNFPQHSGVGAVGAPVV--GPGFGGSFGHGG-F----GYGGLYGGLYGLGGYGGYGGHYGYGGI----IGPWGILRLPGSLWLRGI------------------------------------------------------MGIWGIWP---
            Cp_Beta-O27  MTFS-SLC-YP---ECGVARPCPVTGTCNEPCVRQCQDSEVVIRPSPVVVTLPGPIMSNFPQHSGVGAVGAPVV--GPGFGGSFGHGG-Y----GYGGLYGGLYGLGGYGGYGGHYGYGGL----WGHGGYRGYPGLYGYGGV------------------------------------------------------MGIWGIWP---
            Cp_Beta-O28  MISS-SLC-YP---ECGVARPCPVTGTCNEPCVRQCQDSEVVIRPSPVVVTLPGPILSNFPQHSAVGALGAPVV--GPGFGGSFGHGG-Y----GYGGLYGGLYGLGGYGGYGGRYGYGGL----YGFGG----LGGYGYGGL-------------------------------------------CGSRLSCHRYLSGNCGPC---
            Cp_Beta-O29  MISS-SLC-YP---ECGVARPCPVTGTCNEPCVRQCQDSEVVIRPSPVVVTLPGPILSNFPQHSAVGAVGAPVV--GPGFGGSFGHGG-F----GYGGLYGGLYGLGGYGGYGGRYGYGGL----YGFGG----LGGYGYGGL-------------------------------------------CGSRLSCHRYLSGNCGPC---

Beta B       Cp_Beta-B1  MSFNGPQTGAQGSLPCGVKCSEPYIATASEPCVVKCKDSRVIIYPPPVVVTFPGPILTTCPQESIVASSGP---------PDTG--------VAESAARISAAPRVTGS---LGPHLDRCPASINIRHEA--------QYTPKYSY---------------TYSSRWSHPGKSLETTGYSQT---------------TRNIDRTK
             Cp_Beta-B2  MSCYGLR-----NIPCEVPRPTPAAVTYNEPCVIQCPDSIFESDSPPGIAIIPGPILTTFPHYSVVETSP-------------------------LFDTERSFCSERSLGSQGFMNLYN------------------------------------------------------------------------------
             Cp_Beta-B3  MFSDEEFF-YK/PPQYPSICPQPYIGVWNEPCVTECGDSTAVVFAPPVVVNFPGPTLATCPQDSVVGSSLPRGIIGPYGPGGSLSSGGAFGTGSSFGSSVSSFSSAGSF----------------------GSGGYGGSGGY-------------LGSGGFLGSGGYCG---------------SGGWNPCHYGRCGPC---
             Cp_Beta-B4  MFSDEEFW-YK/PPQYPSIWPQPYIGVWNEPCVTECGDSTAVVFAPPVVVNFPGPTLATCPQDSVVGSSLPRGIIGPYGSGGSLGSGGSFGAGSSFGSSVSSFSSGGSF----------------------GSGGYGGSRGF-------------LGSGGYGGSGGYCG---------------FGGWNPCHYGRCGPC---
             Cp_Beta-B5  MSSYEQLCNTQCYAPCNVTCPQPIVDTCNEPCITSCSDSRAVVYPPLIVVTFPGTLLSFCPQESVEESSA------------------------HVGIRSS---------------------------------------------------------------------------------------------------
             Cp_Beta-B6  MSSYRQLCNTQCYAPCNVTCPRPFVDACNEPCFTSCGDSSAVLYPPPVIVRFPGPILATCPQESVVGSSAP---------FGIG-----------SSLGIGGPYVSGS----------------LGNYGG------SYTSGLS-----------------ARGNGCSYPSSSSQRFTTYRSG-------------SCQPYQTQK
             Cp_Beta-B7  MSSYRQLCNTQCYAPCNVTCPRPFVDACNEPCFTSCGDSSAVLYPPPVIVRFPGPILATCPQESVVGSSAP---------FGIG-----------SSLGIGGPYVSGS----------------LGNYGG------SYTSGLS-----------------ARGNGCSYPSSSSQRFTTYRSG-------------SCQPYETQK
             Cp_Beta-B8  MSFCRDLCKYPSYPSCDVTCPQPFVDACNQPCVTSCGDSSVVVYPPPVVVRFPGPILATCPQESVVGSSEP---------LGIG-----------SSFGYRGSYLSGS----------------SYGYKSLYNDRRSYTPGLS-----------------SLGRGSSDPCSS-RWLNMYGCG-------------PRTQ--QE
             Cp_Beta-B9  MSFYGDPARSQCYLPCEGTCQQPVANVCNEPWVRSGGDSRGVGYAPLVVVTFPGPSSQYLLSGKHDWNGTAK------------------------------------------------------------------------------------------------------------------------------
            Cp_Beta-B10  MSSHRQLVSPRCATPWEVTCPQPGANICSQPCVTSCEDSRVMVYAPPVVVAFPGPILSTCPQKSITGSEVP---------GEMGAYLDLEGHMVLGAHMVSGLPMALGKHMVMKSHLVLGDHMVQTTHGSGGSYGHGGSYSASNSYGARGPYGAGRFLVFRGSYGSGGSYSHSRAYTSRLSPLGTGNSCPYSSQRTSMSHYKNCGPY
            Cp_Beta-B11  MSSCKDLS-CRPSPCYPDICPDPCVVARNEPCITSCADSTAVVYPPPVSVLFPGPILSSSPQHSLVGSTLPAL---PYGARGSFGGGALGGPIGYGSGYGGA---LEGGYGYGGLSGYGGSYGYGGLSGYGGSYGYGGLCGYGGYG-----------GGYGGLCGYG-------------RRY---GGRCYSSRRGSCGPC---
            Cp_Beta-B12  MSSCKDLS-CRPSPCYPDICPDPCVVARNEPCITSCADSTAVVYPPPVSVLFPGPILSSSPQHSLVGSTLPAL---PYRAGGSFGGGALGGPIGYGSGYGGA---LEGGYGYGGLSSYGGSYGYGGLSGYGGSYGYGGLCGYGAGYG-----------GGYGGLCGYGAGYGGGYGGLCGYGRRY---GGRCYSSRRGSCGPC---
            Cp_Beta-B13  MSSCKDLS-CRPSPCYPDICPDPCVVARNEPCITSCADSTAVVYPPPVSVLFPGPILSSCPQHSLVGSTLPAL---PYRAGGSFGGGALGGPIGYGSGYGGA---LEGGYGYGGLSSYGGSYGYGGL------------CGYGGGYG-----------GGYGGLCGYG-------------RRY---GGRCYSSRRGSCGPC---
            Cp_Beta-B14  MSSSKDLC-YPRPPCYPDICPNPYVDAWNEPCVTSCGDSSAVVYAPPVVVRFPGPILATCPQDSVVGSTLPNL---PYGYGSPYGGG------SFGGSVSS----GGA----------------------YGGGYGAGYGG---------------GYGGLYGYGKGYG---------------RKCYSS-RFGSCGPC---
            Cp_Beta-B15  MSSSKALC-YPRPPCYPDICPDPYVDACNEPCVTSCGDSSAVVYAPPVVVRFPGPILATCPQDSVVGSTLPNL---PYGYGGSYGGG------SFSVSVGS----GGA----------------------YGGGYGARYGG---------------GYGGLYGYGKGYG---------------RKCYSS-RFGSCGPC---
            Cp_Beta-B16  MSSSKALC-YPRPPCYPDICPDPYVDACNEPCVTSCGDSSAVVYAPPVVVRFPGPILATCPQDSVVGSTLPNL---PYGYGGSYGGG------SFSGSVGS----GGA----------------------YGGGYGARYGG---------------GYGGLYGYGKGYG---------------RKCYSS-RFGSCGPC---
            Cp_Beta-B17  MSSSKALC-YPRPPCYPDICPDPYVDACNEPCVTSCGDSSAVVYAPPVVVRFPGPILATCPQDSVVGSTLPNL---PYGYGGSYGGG------SFSGSVGS----GGA----------------------YGGGYGAGYGG---------------GYGGLYGYGKGYG---------------RKCYSS-RFGSCGPC---
            Cp_Beta-B18  MSSSKDLC-YPRPSCYPDICPNPYVDAWNEPCVTSCGDSSAVVYAPPVVVRFPGPILSTCPQDSVVGSTLPNL---PYGYGGSYGAG------SFGGSVIS----GGA----------------------YGGRYGAGYGG---------------GYGGLYGYGKGYG---------------RKCYSS-RFGSCGPC---
            Cp_Beta-B19  MSSSKALC-YPRPPCYPDICPDPYVDAWNEPCVTSCGDSSAVVYAPPVVVRFPGPILATCPQDSIVGSTLPNL---PYGYGGSYGGG------SFGGSVGS----RGA----------------------YGGGYGAGYGG---------------GYGGLYGYGKGYG---------------RKCYSS-RFGSCGPC---
            Cp_Beta-B20  MSSSKALC-YPRPPCYPDICPNPYVDACNEPCVTSCGDSSAVVYAPPVVVRFPGPILATCPQDSVVGSTLPNL---PYGYRGSYESG------SFGGSVGS----RGA----------------------YGGGYGAGYGG---------------GYGGLYGYGKGYG---------------RKCYSS-RFGSCGPC---
            Cp_Beta-B21  MSSSKALC-YPRPPCYPDICPDPYVDAWNEPCVTSCGDSSAVVYAPPVVVRFPGPILATCPQDSVVGSTLPNL---PYGYGGPYGGG------SFSGSVGS----GVA----------------------YGGGYGAGYGG---------------GYGGLYGYGKGYG---------------RKCYSS-RFGSCGPC---
            Cp_Beta-B22  MSSSKALC-YPRPPCYPDICPDPYVDAWNEPCVTSCGDSSAVVYAPPVVVRFPGPILATCPQDSVVGSTLPNL---PYGYRGSYGGG------SFGGSVGSSGVYGGV----------------------YGGVYGGGYDG---------------GYGGLCGYGRRYG---------------RKSYSS-RFGSCGPC---
            Cp_Beta-B23  MSSSKALC-YPRLPCHPDICPNPYVDAWNESCVTSCGDSSAVVYAPPVVVRFPGPILATCPQDSVVGSTLPNL---PYRYEGPYGGG------SFGGSGGS----GRA----------------------YGGRYNVGYGS---------------RYGDLCGHGRRFG---------------RKCYSS-RFESCRPC---
            Cp_Beta-B24  MF-AEFLC-WQPRPYCPDICPDPCAYVCNKPFVTSCGDSNGVVYAPPDVMRFPGQALTTCPQDSFVGTVGPRL---FYSSWGGLGGHA-------GGGFGGG---SWGWYGGGSGGGFRGGYGGSFRGGYGGRYGSGYGGGYGGGYG-----------GGFGSGSGGRFGGGLGGSYGY---------GGSYGGNSYANRW-----
            Cp_Beta-B25  MSPVKDLC-CQPGPYYPDICPDPCAYVCNEPCVTTCGDSNAVVFAPPVVVRFPGPTLATCPQDSFVGTSLPNF---PYRLGGGLGGRI-------GGGLGGG---YGGGYGGG--------------------YGGGSFGGFGGG---------------FGGGFGGGIGGGYRGSYGY---------GGRYGRNCYANRWECCPW
            Cp_Beta-B26  MSPVKDLC-YQPRPYCPDICPEPCAYVCNEPCVTTCGDSNAVVFAPPVVVRFPGPTLATCPQDSFVGTSLPNF---PYRLGGGLGGRI-------GGGLGGG---YGGGYGGG--------------------YGVGSFGGFGGGSG-------------GGFGGGFGGGIGGGYRGSYGY---------GGRYGRNCYANRWECCPW
            Cp_Beta-B27  MSPVKDLC-CQPRPYCPDICPDPCAYVCNEPCVTSCGDSSAVVFAPPVVVRFPGPTLATCPQDSFVGTSLPNF---PYTPWGGLGGRA-------GAGLGGG---SWSGYGGG--------------------FGVGAGGGFGGG---------------FGGGFGGGFGAGYGGSYGN---------WGRYGRRCSYTSYRC-PC
            Cp_Beta-B28  MSCCP------PQDCIPDICPCPYIDVRNEPCISSCGDSTAVVYAPPVVVNFPGPTMATCPQDSFIGTSLPNM---PVRAGASYGS---------GGGFSGS---IGSRGSYGA--GFGGGYGGGFRGG----YGGGHGGGYGGGFG-----------GGLRCGYGGSYGYGGPCGYGRRSQRGITVLGGGYSGSSYGNCGPC---
            Cp_Beta-B29  MSYCP------PQDCIPDICPRPYIDVCNEPCISSCGDSNAVVFAPPVVVRFPGPTMATCPQDSIVGSSLPNM---PIRAGGLYGS---------GGGFSGS---ITSGGSYGG--GFGGGYSGGYGGGSSIVYGGGAGGGYGGGAG-----------GGYGAGYGGSYGCGGSRGYIRKSYRSIS--GGGYSGFNRGNCGPC---
            Cp_Beta-B30  MSYCP------PQDCIPDICPRPYIDVCNEPCISSCGDSNAVVFAPPVVVRFPGPTMATCPQDSIVGSSLPNM---PIRAGGSYGS---------GGGFSGS---ITSGGSYGG--GFGGGYSGGYGGGSSIVYGGGAGGGYGGGAG-----------GGYGAGYGGSYGCGGSRGYIRKSYRSIS--GGGYSGFNRGNCGPC---
            Cp_Beta-B31  MSYCP------PQDCIPDICPRPYIDVCNEPCISSCGDSTAVVFAPPVVVRFPGPTMATCPQDSFVGSSLPNM---PIRAGASYGS---------GGGFSGS---ITSGGSYGG--GLGGGYSGGYGGGSSIVYGGGAGGGYGGGAG-----------GGYGAGYGGSYGCGGSRGYIRKSYRSIS--GGGYSGFNRGNCGPC---
            Cp_Beta-B32  MSYCP------PQDCYPDICPRPCIDVRNEPCISSCGDSTAVVFAPPVVVRFPGPTMATCPQDSFVGSSLPNL---PIRPGGSYGGSISYGG-GYGGGYGGGNSVVGSGGGFGGSTGYGGVYGGGAGSGYGGGYGGGAGGGYGG-------------------CYGGSYGSGGSRGYSKKSYRSIS--GGGYSGVNRGNCVQS---
            Cp_Beta-B33  MSYCP------PQDCYPDICPRPCIDVRNEPCISSCGDSTAVVFAPPVVVRFPGPTMATCPQDSFVGSSLPNL---PIRPGGSYGGSLTYAG-GYGGGYGGGNSVVGSGGGFGGSTGYGGVYGGGVGGGYGGGYGGGAGGGYGGGAG-----------GGYGGCYGGSYGSGGSRGYSKKSYRSIC--GGGYSGVNRGNCGPC---
            Cp_Beta-B34  MTYCP------PQDCYPDICPRPCIDVRNEPCISSCGDSTAVVFAPPVVVRFPGPTMATCPQDSFVGSSLPNL---PIRPGGSYGGSITYGG-GYGGGYGGGNSVVGSGGGFGGSTGYGGVYGGGVGGGYGGGYGGGAGGGYGGGAG-----------GGYGGCYGGSYGGSGGSRGYGYNKKSYRSIS--GGGYCGVKSGNYGPC---
            Cp_Beta-B35  MSYCP------PQDCYPDICPRPCIDVRNEPCISSCGDSTAVVFAPPVVVRFPGPTMATCPQDSFVGTSLPNL---PIRPVGSNGGSISYGG-GYGGGYGGGNSVVGSGGGFGGSTGYGGVYGGGVGGGYGGVYGGGVGGGYGGGYGAGVGCGYGGGAAGGYGGCYGGSYGSGGSRGYSKKSYRSIS--GGRSSGVKSGNPEKPSME
            Cp_Beta-B36  MSFNGVPCNDQCHNPCEVTCPQPIVNSSNQPCVVSCGDSRVVIYPPPVVVTFPGPILSTCPQDSIVGSSAA---------SGSR--------ISGSASVISSTPGVTGC---SKPYAE----SVFVRSEP--------QYTPKYSY---------------TYSSPWIHPGNTSGSGHYRSSYVQKACPRNEEPQQNEKQDAEQCS
           Cp_Beta-B17L  MSSSKALC-YPRPPCYPDICPDPYVDACNEPCVTSCGDSSAVVYAPPVVVRFPGPILATCPQDSVVGSTLPNL---PYGYGGSYGGG------SFSGSVGS----GGA----------------------YGGGYGARYGG---------------GYGGLYGYGKGYG---------------RKCYSS-RFGSCGPC---
           Cp_Beta-B18L  MSSSKALC-YPRPPCYPDICPNPYVDAWNEPCVTSCGDSSAVVYAPPVVVRFPGPILATCPQDSVVGSTLPNL---PYGYRGSYGSG------SFGGSVSS----GGA----------------------YAGGYGAGYGG---------------GYGGLYGYGKGYG---------------RKCYSS-RFGSCGPC---
                                                       ****    ****
```

Supplementary Figure S10. Amino acid sequence alignment of beta-keratins of *C. picta bellii* (Cp). Amino acid sequences were aligned with the Multalin algorithm. Beta-keratins form 4 groups encoded by genes at different loci. EDbeta, Beta A, and Beta B proteins are encoded by genes arranged in this order within the EDC whereas Beta O proteins are encoded by genes outside the EDC. The group of Beta A proteins shows high sequence similarity to Beta O proteins. Beta-A1L1, A1L2, B17L, and B18L are located on unplaced single-contig-scaffolds. Beta A and B proteins are numbered according to the order of their genes in the EDC. Red, conserved in 90% or more sequences; blue, conserved in 50% or more sequences; /, additional sequence was removed to allow alignment of the N-terminus. Asterisks indicate positions of 2 beta-sheets.

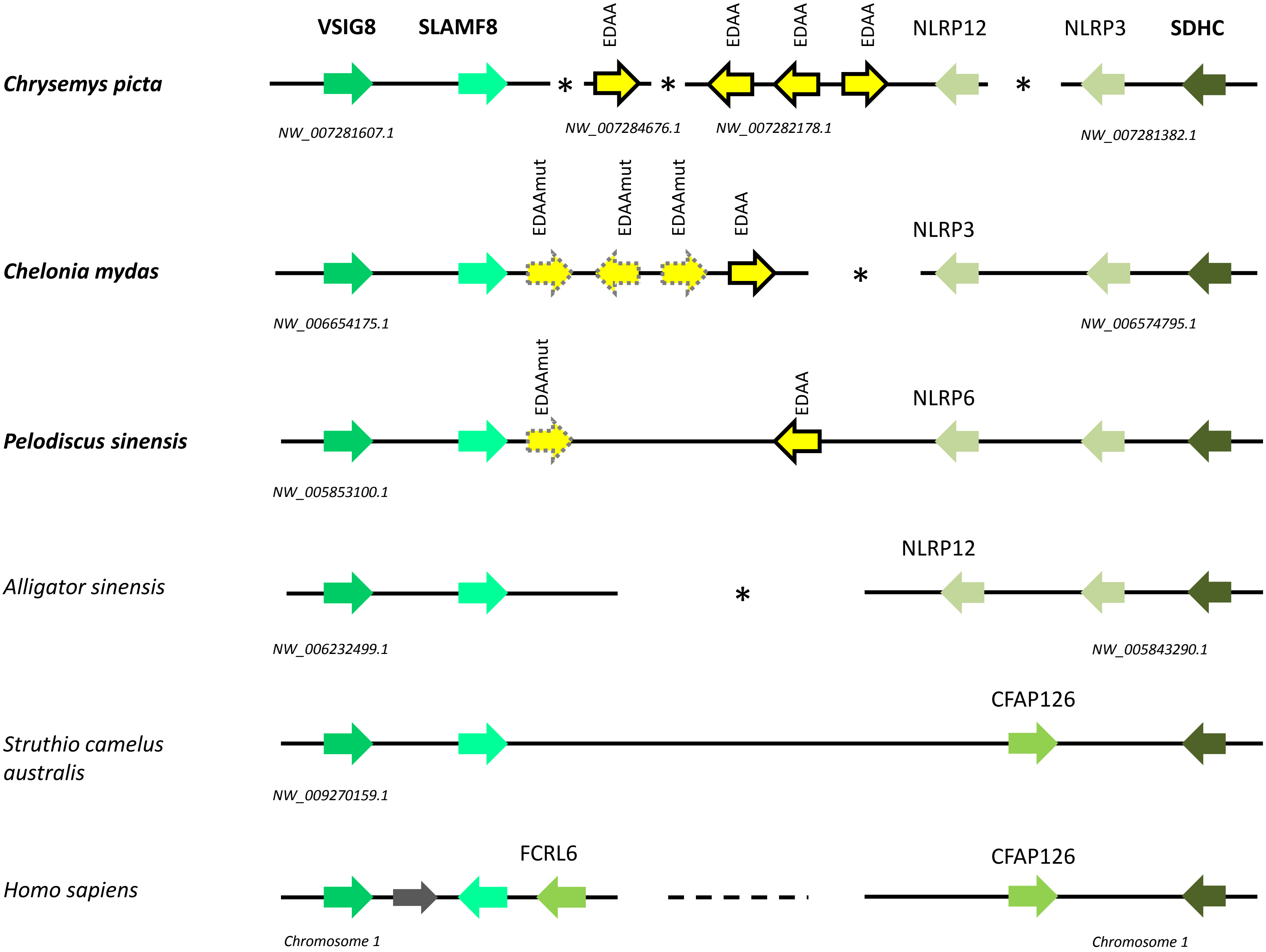

Supplementary Figure S11. Turtle EDAA gene locus outside of the EDC and homologous loci without EDAA genes in other amniotes. The conserved genes VSIG8, SLAMF8 and SDHC flank a chromosomal locus that contains EDAA genes in turtles but not in other amniotes. The relative arrangement and the orientation of genes is schematically depicted. The schematics are not drawn to scale. For detailed information about EDAA gene names and positions, see Suppl. Tables S1-S4. Colors indicate gene homology. EDAA genes carrying deleterious mutations have frames with a broken line. The numbering of NLRP (NLR family, pyrin domain containing) proteins in reptiles is uncertain. GenBank accession numbers are shown below the various scaffolds. A broken lines indicates a long region of human chromosome that contains too many genes to be shown here. The genome of the ostrich (*S. camelus australis*) is shown as representative of phylogenetically basal birds. Asterisks indicate genome sequence gaps.

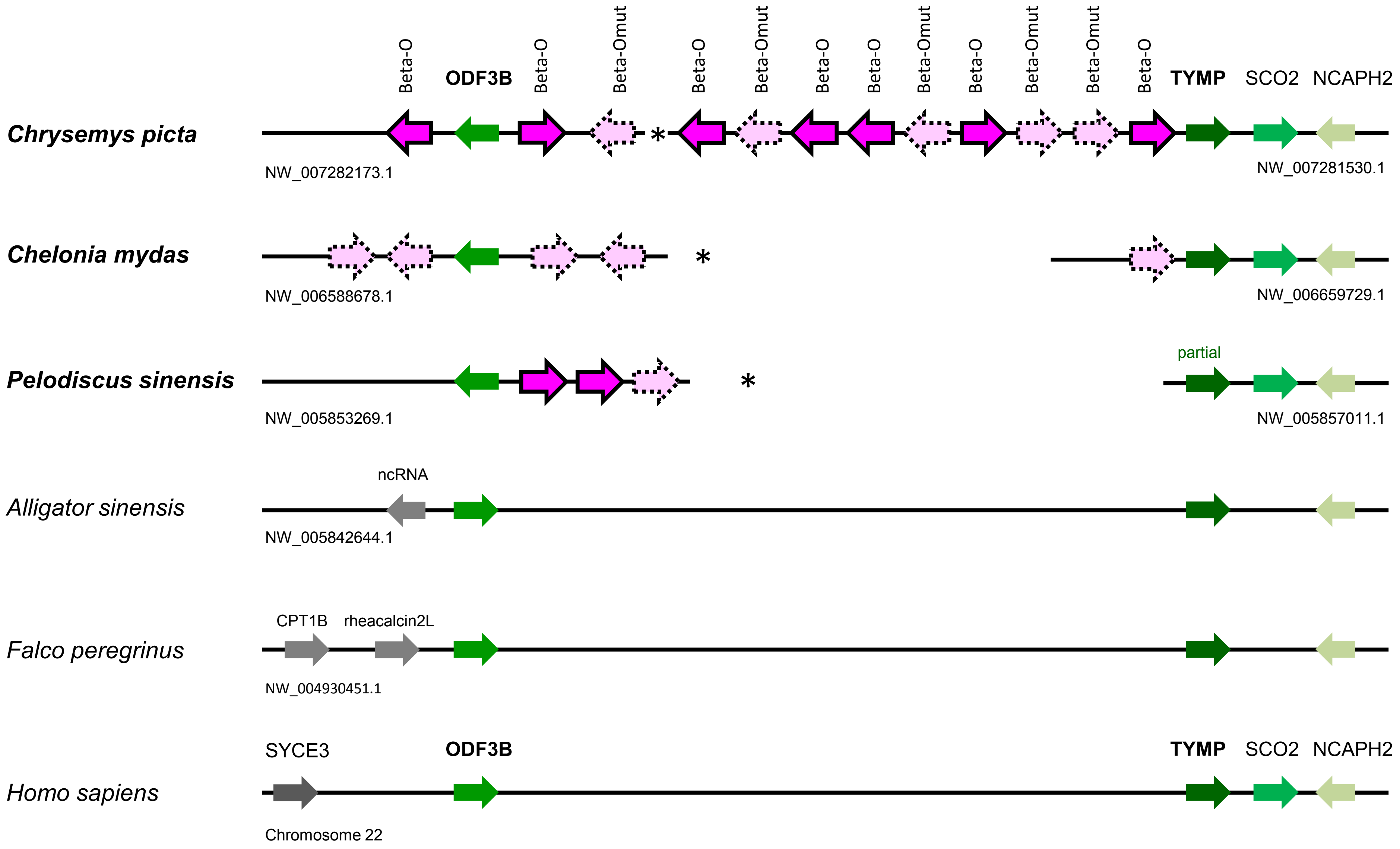

Supplementary Figure S12. Beta-keratin (Beta-O) gene locus outside of the EDC in turtles and homologous loci without beta-keratin genes in other amniotes. The conserved genes *ODF3B* and *TYMP* flank a chromosomal locus that contains beta-keratin genes in turtles but not in other amniotes. The relative arrangement and the orientation of genes is schematically depicted. The schematics are not drawn to scale. Colors indicate gene homology. Pink arrows indicate beta-keratin genes comprising apparently functional open reading frames. Light pink and frames with broken lines indicate the presence of deleterious mutations. For detailed information about gene names and loci, see Suppl. Tables S1-S4. GenBank accession numbers are shown below the various scaffolds. In birds, represented here by peregrine falcon (*F. peregrinus*), this locus is not well conserved but consistently devoid of beta-keratin genes. Note that high sequence similarities to the genes on the above scaffolds indicate that additional genes (also designated Beta-O) on other short scaffolds (Suppl. Tables S2-S4) belong to the same locus. Asterisks indicate genome sequence gaps.

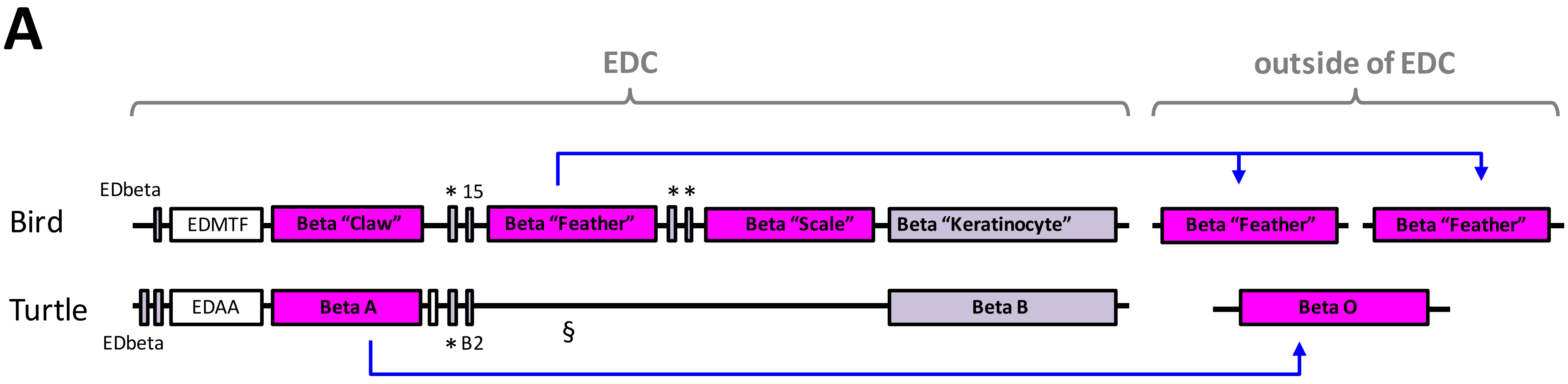

(continued on the next page)

Supplementary Figure S13. Gene locus comparison and phylogenetic analysis of beta-keratins (also known as corneous beta-proteins) of a turtle (*C. picta bellii*) and a bird (*G. gallus*). (A) Schematic diagram of beta-keratin gene loci of a bird (chicken, *G. gallus*) and a turtle (western painted turtle, *C. picta*). For an overview of the genes of the surrounding locus, see Figure 2. Clusters of more than 2 similar beta-protein genes are shown as long boxes (length not proportional to the number of genes) whereas single genes are depicted as short boxes. The names of the gene clusters of the turtle are defined in the main text. EDMTF (Strasser et al. 2014) and EDAA genes are indicated as white boxes whereas beta-keratins are indicated by pink and violet shading with pink clusters being closely related according to molecular phylogenetics (panel B). The names of chicken beta-keratin clusters clade are adapted from Ng et al. (2014) and Greenwold et al. (2014). Putative gene translocations linking clusters of related beta-keratin genes (see panel B) within the EDC (left) and outside of the EDC (right) are indicated by blue arrows. Genes marked by asterisks belong to the groups Beta-B or bird Beta "Keratinocyte". The turtle gene EDAA10 is located between the clusters Beta A and Beta B of the turtle. §, the length of the line between Beta B2 (orthologous to chicken beta 15) and the rest of the Beta B cluster of the turtle is not proportional to the physical distance but indicates that this locus does not contain orthologs of avian feather and scale beta-keratins. **(B, next page)** Maximum likelihood phylogeny of beta-keratins of the western painted turtle *C. picta* (Cp, in red) and chicken *G. gallus* (Gg, in blue). Support of phylogenetic groups was computed by the ultrafast bootstrap approximation approach (UFBoot) (see Materials and Methods for more information). Since UFBoot support values behave like posterior probabilities, branches with support values of at least 90% and 95% are regarded as supported and strongly supported, respectively. Branches with support lower than 80% are not shown (see Materials and Methods for more information). For clarity, mid-point rooting was used to draw the tree, however, the true position of the root is unknown. The strongly supported monophyletic groups of avian feather, scale and claw Beta proteins as well as the monophyletic group of turtle Beta-A with Beta-O proteins are indicated (pink fonts) on the right. The relationships within these clades could not be resolved because of the limited phylogenetic information in the underlying sequence alignment. Partial Cp beta-keratins (Suppl. Fig. S2C), the highly derived and possbily pseudogenic sequence Cp_Beta-B9, Cp beta-keratins Beta-A1L1, A1L2, B17L, and B18L encoded by genes on unplaced single-contig-scaffolds, and incomplete feather beta-keratin sequences (Ng et al. 2014) were not included in the phylogenetic analysis. In the names of feather beta-keratins encoded by genes outside the EDC (labeled FK), the chromosome number is indicated after the species code. For example, Gg27 indicates *Gallus gallus* chromosome 27). Other labels of Gg sequences: F, feather beta-keratin encoded by gene within the EDC on chromosome 25; S, scale beta-keratin (EDC); C, claw beta-keratin (EDC); K, keratinocyte beta-keratin (EDC).

Suppl. Fig. S13B

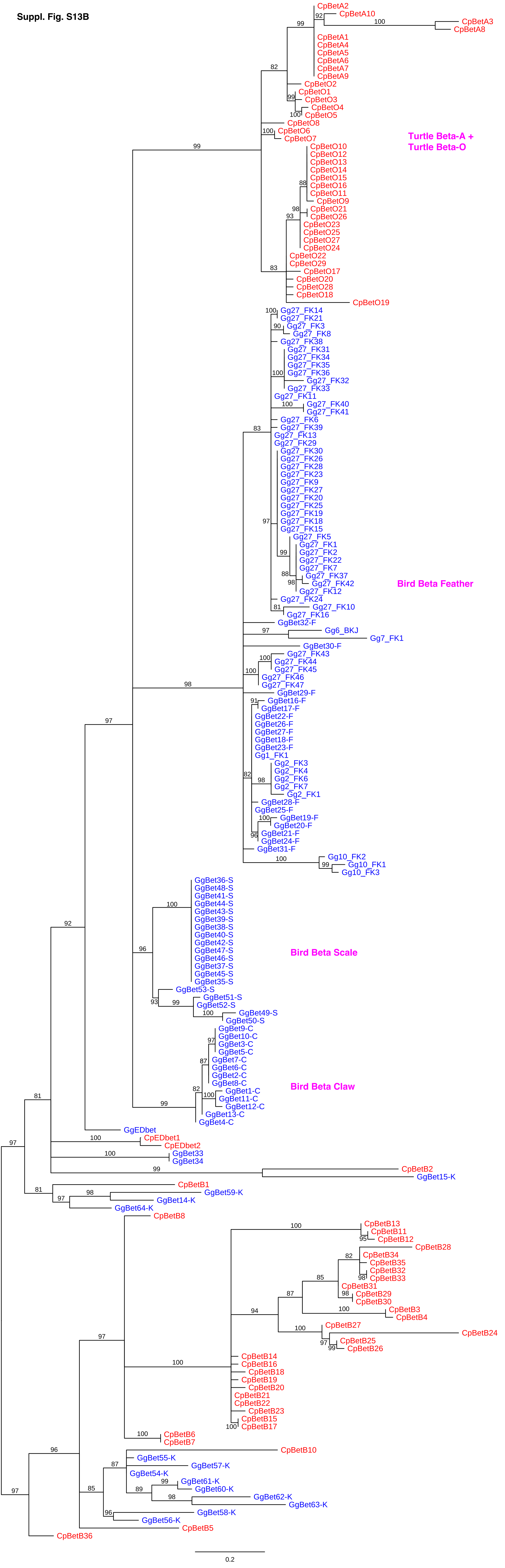

A

```
          1                                                         60
Cp_EDQL   MCSREPRGCHDSGSSSCHDSGSSTCHSSGGGSCHDVKPL----------PQCPT-PVPCQ
Gg_EDQL   MCSRADRGCHSSESSSCH-SGGSSCHGSEEVTCHEVSAVQDGTPVVVLQPQCPVVTVPTQ
          ****  **** * ***** ** * ** *    ** *             ****   ** *

          61                        88
Cp_EDQL   --TTTLPC---QQQTKQPCQWPPQKHQK
Gg_EDQL   GPVAPVPCQQQQQQIKQPVQWPTQQ-QK
                **   *** *** *** *  **
```

B

```
          1                                                         60
Cp_EDP3   MSSDQQQCKQTCPPPPKCQEKCPPPCKEPVKTPKCQEKCPPPSKEPKCPPPKQSQDWKQC
Gg_EDP3   MSSHQQ--KQQQQIPAQCQQKCPPKGVEQCQAPKGQTKCPVKSIPQQ---QQQQQCPKQK
          *** **  **    *  ** ****   *    ** * ***  *         * *  **
```

Supplementary Figure S14. Examples of proteins encoded by orthologous SEDC genes of the western painted turtle and the chicken. Orthology of genes was inferred from reciprocal highest sequence similarity and gene locus synteny. Amino acid sequences of EDQL **(A)** and EDP3 **(B)** proteins were aligned. Note that EDQL of the chicken was previously named EDQM3 (Strasser et al., 2014). The EDP3 gene of the chicken had been missed in our previous study of the chicken EDC (Strasser et al., 2014). Here, we identified the coding sequence of chicken EDP3 in the EDC on chromosome 25 (Accession number: NC_006112.2, nucleotide positions 1108303-1108470, reverse orientation). Asterisks below the alignments indicate identical amino acid residues. In the alignment of EDQL sequences, the carboxy-terminal sequence similar to the carboxy-terminus of loricrin (Suppl. Fig. S6B) is underlined. Amino acids involved in cross-linking via cysteine bridges (C) and transglutamination (Q, K) as well as proline residues (P) are highlighted by color-shading as in Suppl. Fig. S1. Cp, *Chrysemys picta bellii*; Gg, *Gallus gallus*.

A

```
                              1                                                          60
           lizard (Aca) MCESRIYAAGREPYFNLNSTWYDPAGSWLDTRRKPFHYTVNTSCVPCCNKNNNCDVPRRG
            snake (Oha) MPEERIYSSGREAYFNLNSTWYDPAGSWLDTRRKPFRYVDNTACVTCCNPRSN--VPRRG
        alligator (Asi) M----TCSSGRESYFNLNSTWYDPSGSWLENHRIPLCYADDSCCGGC-----NPDVRGVG
          chicken (Gga) M----IYSSGRESYFNLNSTWYDPAGSWLDTRRTPFRYGYNNCCSSRC---DGEGVEGMR
hardshell turtle (Cpi) M----IYSSGRESYFNLNSTWYDPAGSWLDTRRTPFTYAYSTCCSSGG---CPRG-----
hardshell turtle (Cmy) M----IYSSGRESYFNRNSTWYDPAGSWLDTRRTPFTYAYSTCCSSGC---GPRG-----
softshell turtle (Asp) M----VYSSGREPFFNRNSTXYDPAGSWLDTRCTPFTYAY????????????????????

                              61                                                        120
           lizard (Aca) GHNYRCYSYRQSTCTPECNPRLPCGFRNPSGGPRDYWGRPIGDSCDGRTGGYYSNEESVN
            snake (Oha) GHNYRCYCYRQCTCTPGGNPRVTCCVHNPSGGPRDYWGRPIGDACDGCTGGHYSHAGSDC
        alligator (Asi) GHNYRPCWYRRSVCSEAERGSSSGYCGSEDSGCAR---RPTLGYSDGC-GGYRRGPDRCN
          chicken (Gga) GHNYRHYGYRQPVCSERCQGYSTAESCHGGGGSSCA-RRPT--YSYGSTGGCQGYGRSVC
hardshell turtle (Cpi) GHDNRCYEYRRSGCGENCHGSSG--SCHGSGGHCCV-RRPS--YFHGYSGGCHGHGRSVC
hardshell turtle (Cmy) GHDNRCYEYRRSGCAENCHGSSG--SCHGSGGHCCV-RRPS--YFHGSSGGCHGHGWSVC

                              121                                                       180
           lizard (Aca) GSCCRASGGCGSGGG--ACAKPSSSIGGCGGGVCAEPGCQSSGRCGGRRRGLCSEPGCGL
            snake (Oha) GSCCGSLGGCGTGGRTMACAQPCAT----SGGVCAEPGCRPAGRGVCAEPCITSSGGCS-
        alligator (Asi) GECSSHEFGRRPTYHYAADVYLANERLACSEGCHGSSGGFYGSSGGCHRRRRCGEPC---
          chicken (Gga) SE---------------------------R-CQGSSGSCHGGGGSSCVR----------
hardshell turtle (Cpi) SE---------------------------RSCHGSGSSCHGSG-SSC------------
hardshell turtle (Cmy) SE---------------------------RSCHSSGSSCHGSG-SSC------------

                              181                                                       240
           lizard (Aca) FRRRRSVCSETCSRSSRGCGSGGCAGPQISFSGGCGGRGLCSEPG-C---GIARRRQSVC
            snake (Oha) --SGRGVCAEP---GCRPAGRGVCAEPCITSSGGCR-TGVCAEPT-CTPSGYGRRRRGVC
        alligator (Asi) ----HGSGSYGSSRGCHGRRRSVCGEPCHDSGSSGYLQRVCVKPGPCIPRCPPRQKYVRS
          chicken (Gga) ----RPTYSYGSTGGCQGYGRSVCSERCQGSSGGGF-HSSGQQPQCSEP--------VQY
hardshell turtle (Cpi) ---------HGSGSSCHGSGSSC------HNTSGAC-HST---PIYVKP--------KQY
hardshell turtle (Cmy) ---------HGSGSSCHG------------TSGAC-HSA---PIYVKP--------KQH

                              241                                     284
           lizard (Aca) SETYSRSSRGCQPYARGAC--VGPQSSVSGGCGARGVCSEL---
            snake (Oha) FEPCSGTSNGC---------------------------------
        alligator (Asi) TQSCCIPVQTYCAPVQAYCPPVGKYSSGGQQCKQTSKLPILKAK
          chicken (Gga) IPQCCPMPVPVQQVPTAKCIPHQQQQQ-----QQVCKVPARKIK
hardshell turtle (Cpi) VQQCCP---PVQQC----CLPVKK-----------CCPPVQKC-
hardshell turtle (Cmy) VQQCCP---PVQKC----CPPMQQ-----------CCLPVKKC-
```

Suppl. Fig. S15. The *EDWM* gene is deleted in the soft-shell turtles. Continued on the next page.

B

```
     1                                                                                                  100
Psi  GAGGGCACCATGGGGCTGCCTGAAGAAAGG-------CAAGTTTGCAGCGGGGAGAATTGGCCAGGACCCAACGGCTGGAGTCGGATCCCTGGGA----
Cpi  GAGAGCACCATGGGGCTGCCTGAAGAAGAGAGCGCCAACAAGTTTGCATGGGGTGAATTGGCACATTGCCCTGTGGCTGATTCTGATCCCCAGTTGCAT

     101                                                                                                200
Psi  ---------------CTTGCCATTTTTCACCTGGCCAGGGAAACCTGT-----------------------------------------------------
Cpi  ATGGCTGGGGAAATCCATTTCAGTGTAAACCTAGAGAGGGCATCTTGGAACAGGCTGGCCAGTCCCCAGAGGGGGGTGTTGATATCGCCCTTAGATTCGA

     201                                                                                                300
Psi  -------------------------------------------------------------------GTCAATGTAAATGCGGGGTGTTTCTCA-----
Cpi  GGCTTTCAGGTTAAGAACCACTGGCCCATGTGTGGGGTAGACAAACAGGAATTTCTATCTGAGGAGAGTCAATGGAAATTCAGGGACTTATCCCATGTGG

     301                                                                                                400
Psi  ------------------------------------------------------------------------------GAAGGGCCGAGCCGCTTCCCAG
Cpi  TCTCTTTCAGCTTCACCTCGGCTGCAGAATGATTTACTCTTCTGGAAGGGAATCCTACTTCAACTTGAACTCCACCTGGTATGACCCTGCAGGTTCCTGG
                                M  I  Y  S  S  G  R  E  S  Y  F  N  L  N  S  T  W  Y  D  P  A  G  S  W

     401                                                                                                500
Psi  ACACAGTCCCCCCTTA------------------------------------------------------------------------------------
Cpi  CTGGACACCCGGCGCACCCCCTTCACCTATGCTTATAGCACCTGCTGCAGCAGTGGTGGCTGTCCAAGGGGAGGCCATGATAACCGATGCTACGAGTATC
      L  D  T  R  R  T  P  F  T  Y  A  Y  S  T  C  C  S  S  G  G  C  P  R  G  G  H  D  N  R  C  Y  E  Y

     501                                                                                                600
Psi  ----------------------------------------------------------------------------GACACAATTGGAGTTATTTGATTT
Cpi  GACGATCGGGCTGTGGTGAGAATTGCCATGGGTCGTCGGGGTCGTGCCACGGCAGTGGAGGCCACTGCTGTGTCAGGAGGCCATCGTACTTCCATGGATA
     R  R  S  G  C  G  E  N  C  H  G  S  S  G  S  C  H  G  S  G  G  H  C  C  V  R  R  P  S  Y  F  H  G  Y

     601                                                                                                700
Psi  TTCT------------------------------------------------------------------------------------------------
Cpi  TTCTGGAGGATGCCACGGCCATGGGCGGTCGGTCTGTTCTGAGCGGTCATGCCACGGTTCTGGATCGTCATGCCACGGTTCTGGGTCGTCATGCCACGGT
       S  G  G  C  H  G  H  G  R  S  V  C  S  E  R  S  C  H  G  S  G  S  S  C  H  G  S  G  S  S  C  H  G

     701                                                                                                800
Psi  ----------------------------------------------------------------------------------------------------
Cpi  TCTGGATCTTCATGCCACGGTTCTGGATCGTCATGCCACAATACCTCTGGAGCATGCCACAGTACACCAATTTATGTGAAGCCAAAACAATATGTGCAAC
      S  G  S  S  C  H  G  S  G  S  S  C  H  N  T  S  G  A  C  H  S  T  P  I  Y  V  K  P  K  Q  Y  V  Q

     801                                                                                                900
Psi  ----------------------------------------------------------------------------------------------------
Cpi  AGTGCTGCCCTCCAGTGCAACAGTGCTGTCTTCCAGTGAAAAAGTGCTGCCCTCCAGTGCAGAAGTGCTGAAAACCAAGGCCAAAGCGTCTTCCAAGTCA
     Q  C  C  P  P  V  Q  Q  C  C  L  P  V  K  K  C  C  P  P  V  Q  K  C  -

     901                                                                                               1000
Psi  -----------------------------------------------------CCCACAACAGCAATACTTTTCCTGTGCTTGTGACATGGCCTTACAC
Cpi  ACAGCTGCAAAATCAAGTCTGCAAAATTCCAACACGCAGCTGAAGTGACAGCTACAAGTAACAGCAATGAATCTTCTGCTCTCATGACGTTGCTTTACAT

     1001                                                                                              1100
Psi  ATATAGCCCTTGGCCTGGCTTCTTTCCTTCACACATTGGCCTGCCATGGTTATTTGGCTCCTAT--AGCAGCTGGTGTTTGCACTGCCCTGTGAGGAAGC
Cpi  TTCTACAGCTTGGCATGACTTGTTTCATTCACAAATTGCACTTTCTCTGTTGCTTTGATTATTATAACCAGTTAATGCTTGTAATGCACTTTGAAGAGGT

     1101                                                                                              1200
Psi  ACCCTACAATACACGTGTGCCGTCATGCCCGTATGGGCTCTTTCTGGTGTATCATTCGTATCCCTGGCCATGCGC---TGTCCCATGTTCTTGTGGGATA
Cpi  AACCCACTGTATAAGTGTGACATTTTATCATTAAGGGCTTTTTCTGAGGGATCCTTTATATTCTTGGGCTATGCAATGTGAAATATTTTCTGCTGGGATA

    1201                                                       1260
Psi  ATTTCTCCCATTTCCTTTCCGCTTCAGGGGCCAGTTGTAGAGGAGAAAACAGTAAAACCC
Cpi  ATTTCACCCACTACCTTCCCGCTTCGCTGTCCACTTGTAGAGGTGAAGACAATAAAACTT
```

Supplementary Figure S15. The *EDWM* gene is deleteriously mutated in soft-shell turtles. (A) Amino acid sequence alignment of EDWM proteins. *EDWM* is conserved in representatives of all main clades reptiles whereas the conceptual translation of an *EDWM* gene fragment of the softshell turtle *Apalone spinifera* (Asp) shows inactivation. An "X" on black background indicates the premature end of EDWM because of an in-frame stop codon in the *Apalone spinifera* sequence. Because of the end of the genomic sequence contig, the conceptual translation of Asp EDWM is incomplete (indicated by question marks). Red letters indicate residues present in all species and blue letters indicate residues present in more than 50% of the species (except *Apalone spinifera*). Aca, *Anolis carolinensis*; Asi, *Alligator sinensis*; Cmy, *Chelonia mydas*; Cpi, *Chrysemys picta bellii*; Gga, *Gallus gallus*; Oha, *Ophiophagus hannah*. **(B)** The coding sequence of *EDWM* has been lost in the softshell turtle *Pelodiscus sinensis*. DNA sequences from the predicted *EDWM* locus within the EDC region of *Pelodiscus sinensis* (Ps) and *Chrysemys picta bellii* (Cp) were aligned. Identical nucleotides are shown in red. The coding sequence of the *EDWM* gene of *C. picta* is highlighted by yellow shading. The amino acid sequence is shown below the coding sequence. Cp, Chrysemys_picta_bellii-3.0.3 Scaffold107, whole genome shotgun sequence, gi|636526453:c1011496-1010237 (reverse complement); Ps, PelSin_1.0 scaffold1810, whole genome shotgun sequence, gi|557455322:c36333-35817 (reverse complement).

A

```
           1                                                          60
  Cp_EDP1  MPYYGQQHKH--LPAPVCVTKCSQPCPPQYEQHCVPKCRPVYVTKCPPLYGPQYAYPCAP
  Cm_EDP1  MPYYGQQHKQLCLPPPACVTKCSQPYPPQYEQQCVPKCRPVYVTKCPPWYGPQYAYPCAP
Ps_EDP1L2  MTYYGRKHQQHCLPSPACVAKCPQPCRPQYEQHCAPKCQPVYVTKCPPLYGPQYAFPCAA
 As_EDP1p  XXXXXXXXXXXXXXXXXXXXXXXXXXXXXXXXXXXXXXXXXXXXXXXXXXXXXXXXXXXX

           61                                                        120
  Cp_EDP1  QCPPRCVTKCPPRCVTKCPPPCVTKCPPPCVTKCPPPCVTKCPPPCVTKCPPPCVTKCPP
  Cm_EDP1  QCPPPCVTKCPPPC----PPPCVTKCPPPC------------------------------
Ps_EDP1L2  QCPPRCVTKCPPPCVTKCPPQCVTKCPPPCVTKCPPQCVTKCPPPCVTKCPPQCVTKCPP
 As_EDP1p  XXXXXXXXXXXXXXXXXXXXXXXXXXXXXXXXXXXXXXXXXXXXXXXXXXXXXXXXXXXX

           121                                                       180
  Cp_EDP1  PCVTKCPPPCVTKCPPPCVTKCPPPCVTKCPPPCMTKCPQQCVTQCP-------------
  Cm_EDP1  ----------------------PPPCVTKCPPPCVTKCPQHCVTQYP-------------
Ps_EDP1L2  PCVTKCPPQCVTKCPPPCVTKCPPQCVTKCPPPCVTKCPPQCVTKCPPPCVTKCPPQCVT
 As_EDP1p  XXXXXXXXXXXXXXXXXXXXXXXXXXXXXXXXXXXXXXXPPCVTKCPPPCVTKCPPPCVT

           181                                               232
  Cp_EDP1  ---------------------------GQYQSGKVQISSHCKKYCSAPKWPW
  Cm_EDP1  ---------------------------DQYQSGKVQISSHGKKYCSGPKWPW
Ps_EDP1L2  KCPPQCVTKCPPRCVTKCPQQCVTQYPGQCQSGNIKMSSQCKKYCSAPNWPW
 As_EDP1p  KCPPPRVTKCPPRCVTKCPQQCVTQYPGQCQSGNIKMSSHCKSYCSTPKWPW
```

B

```
           1                                                          60
  Cp_EDP2  MASRQNQQQRKQTLTLPPALSNATSEPAPPPEAVPEPCPATVEEPENSPQEEEGPQEEYK
  Cm_EDP2  MASQQNQQQRKQTLTLPLALSNATSEPAPTPEAGPEPCPATVEERENSPQEEEESQEEYK
  Ps_EDP2  MASPQNQQQRRQSLPLPPALSNAAPEPEPSPG------PRTVKEPENAPREEEKPQKE--
 As_EDP2p  MASPQNQQQRRQILTLPPALSNATPEPEPSPEVARDPGPTTVXXXXXXXXXXXXXXXXXX

           61                                                        120
  Cp_EDP2  QPLNQPLGPAPELEPEPVLCPEP--ESNPPEVKEIEYLQPDHQQYKHPPTLPPAPGMETS
  Cm_EDP2  RPLNQPLGPAPELEPEPVLGPEP--ESNPSEVKEIEYLQLDQQQYKHPPTLPPAPGIETS
  Ps_EDP2  -PLDQPPGPVPELEPEPEPEPEPAPEPNPPEAEEAGYLQPEQQQYKQPPALPPAPGAETS
 As_EDP2p  XXXXXXXXXXXXXXXXXXXXXXXXXXXXXXXXXXXXXXXXXXXXXXXXXXXXXXXXXXXX

           121                                               172
  Cp_EDP2  KEYQQAESE--PELGRCPPPIREPEGPPFVQPSSPVEEQQQKQPHHWPPKRK
  Cm_EDP2  KEYQQAEPELEPEPGRCPPPISEAEGPLFVQPSSPVEEQQQKQPHHWPPKRK
  Ps_EDP2  TECEEAKPEPEPEPGRCPPPISEPEGPGPVQPSPPGEEKQQKQPCRWPPARK
 As_EDP2p  XXXXXXXXXXXXXXXXXXXXXXXXXXXXXXXXXXXXXXXXXXXXXXXXXXXX
```

Supplementary Figure S16. Amino acid sequence alignments of orthologous EDC proteins of 4 species of turtles. Continued on the next page.

C

```
           1                                                          60
Cp_EDQL    MCSREPRGCHDSGSSSCHDSGSST------------------------------CHSSGG
Cm_EDQLp   MCSREPHGCHDTGSSSCHDTGSSSSPDTGSSFCXXXXXXXXXXSSSCHDTGSSSCHGSGG
Ps_EDQL    MCSREPRGCPDSERSSCPSSERSS------------------------------CHGSEA
As_EDQLp   MCSREPRGCPDSERSSCPDSERSXXXXXXXXXXXXXXXXXXXXXXXXXXXXXXXXXXXXX

           61                                       102
Cp_EDQL    GSCHDVKPLPQCPTPVPCQTTTLPCQQQTKQPCQWPPQKHQK
Cm_EDQLp   GTCHDVKPLPQCPIPVPCQTTTIPCQQQTKQPCQWPPQKHQK
Ps_EDQL    TTCHDVKPHPQYPTTVPCQTPTSPCQQQTKQTCPWPPQKHQK
As_EDQLp   XXXXXXXXXXXXXXXXXXXXXXXXXXXXXXXXXXXXXXXXXX
```

D

```
           1                                                          60
Cp_EDYM1   MSYFAYQYKQRNYTPYSTTRLIPHAEPCVVKGPAPRVTKCADPCAVKHPAPCTTKCRDPC
Cm_EDYM1   MSYFAYQYKQRNYTPYSATRLVPPAEPCVVKGPAPPGTKCAETCAVKHPAPCTTQCRDPC
Ps_EDYM1   MSYFAYQYKQRNYTPYSTTRLLACAEPCVVKGPAPCGTKCVEPCATKRPAPCVPKCRDPC
As_EDYM1p  XXXXXXXXXXXXXXXXXXXXXXXXXXXXXXXXXXXXXXXXXXXXXXXXXXXXXXKCRDPC

           61                                                        120
Cp_EDYM1   AGKPSVPCATKCFEPHAQRHPAKHYPKFSEPAGVKCSTPCDTRYHEPYGLIHPQPFPERW
Cm_EDYM1   AAKPSVPCATKCFEPHAQRHPAQYIPKFSEPVGVKCSTPCVTRYHEPYGLIHPQPFPERW
Ps_EDYM1   AGKAPVHCEPKCLEPHAQRGPAHCAPKFSEPAGVKCSVPWVPRCHEPYGPIPARPFPERW
As_EDYM1p  AGKTSIHCEPKCLEPHAQRGPAHCAPKFSEPVGVKCSVPWVPRCHEPYG-----PVPERW

           121                                              170
Cp_EDYM1   NPCAPPYVHPYVTGYPQACGPTYVPSFPKYPYPYAPQWPNTWGYGNCGPC
Cm_EDYM1   NPCAPPYVHG---GYPQACGPTYVPSFPKYPYPYAPQWPDTWGYGNCGPC
Ps_EDYM1   NPCAPPYGQPFVTGYPQACGPSYGPSFPKYSYPCAPQWPGGWGYGGCGPC
As_EDYM1p  NPCAPPYGQPFVTGXXXXXXXXXXXXXXXXXXXXXXXXXXXXXXXXXXXX
```

Supplementary Figure S16. Amino acid sequence alignments of orthologous EDC proteins of 4 species of turtles. The amino acid sequences of EDP1 (A), EDP2 (B), EDQL (C) and EDYM1 (D) of *Chrysemys picta* (Cp), *Chelonia mydas* (Cm), *Pelodiscus sinensis* (Ps) and *Apalone spinifera* (As) were aligned using the Multalin algorithm. Red fonts, residues conserved in all sequences; blue fonts, conserved in 50-75% of the sequences; p, partial amino sequence; X, unknown amino acid residue. Note that the highly fragmented genome sequence of *A. spinifera* did not allow the prediction of complete sequences.

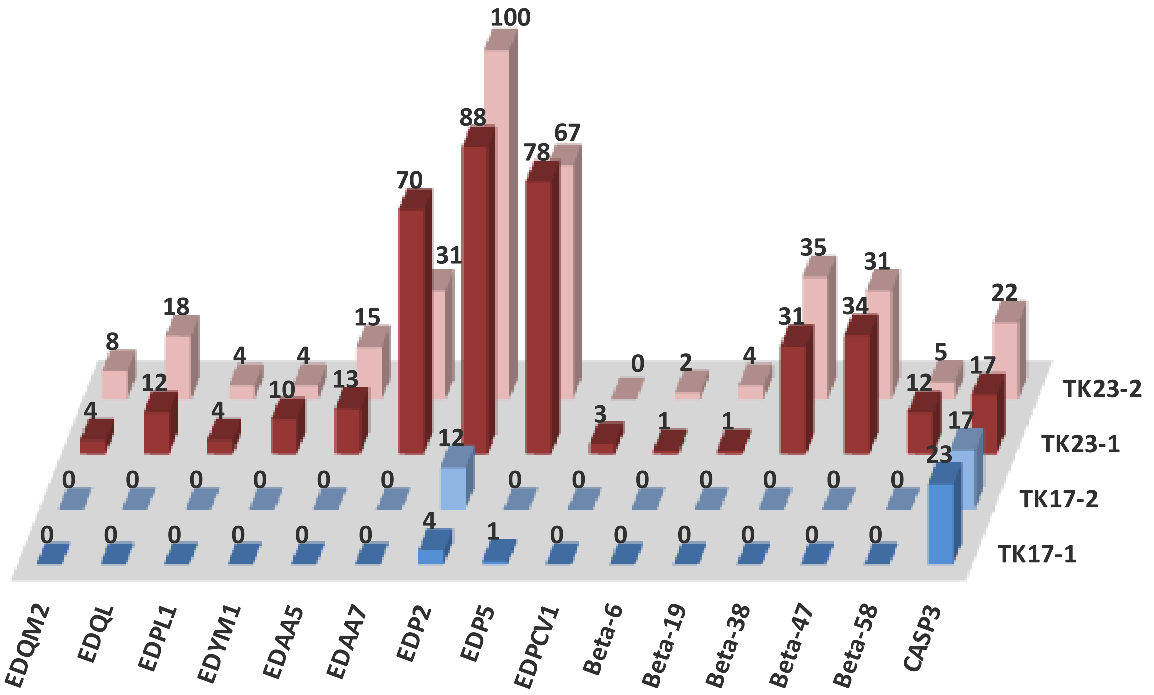

Supplementary Figure S17. RNA sequencing (RNA-seq) data suggest upregulation of EDC gene expression during epidermal maturation in embryos of *Pelodiscus sinensis*. To estimate the expression levels of EDC genes during embryonic development of the soft-shelled turtle *P. sinensis*. RNA-seq data deposited in the GenBank sequence read archive (SRA) (Wang et al., 2013) were screened by tBLASTn using the sequence of the first 17 amino acid residues of each protein as query. The RNA-seq reads yielding a 100% match to the query sequence were counted and plotted over each sample (Tokita and Kuratani (TK) development stage - number of replicate). Data from 2 biological replicates of various embryonic stages were analyzed. The results obtained for the two final developmental stages, TK17 and TK23, of Wang et al. (2013) are shown. A subset of the EDC genes of *P. sinensis* (Supplementary Figures S2B, C) were investigated. The ubiquitous pro-apoptotic protease, caspase-3 (CASP3), was used for comparison. As the amino-terminus of the predicted caspase-3 protein of *P. sinensis* (XP_006128558.1) appears to be incorrect, we used the sequence of residues 28-44 as query for CASP3. Note that the expression levels of all EDC genes increased from development stage TK17 to stage TK23 whereas the expression of CASP3 remained unchanged. Accession numbers of transcriptome data: DRX001551 (TK17, sample 1), DRX001552 (TK17, sample 2), DRX001553 (TK23, sample 1), DRX001554 (TK23, sample 2). References: Tokita M, Kuratani S. 2001. Normal embryonic stages of the chinese softshelled turtle Pelodiscus sinensis (Trionychidae). *Zool Sci*. 18:705-715. Wang Z, Pascual-Anaya J, Zadissa A, Li W, Niimura Y, Huang Z, Li C, White S, Xiong Z, Fang D, et al. 2013. The draft genomes of soft-shell turtle and green sea turtle yield insights into the development and evolution of the turtle-specific body plan. *Nat Genet*. 45:701-706.

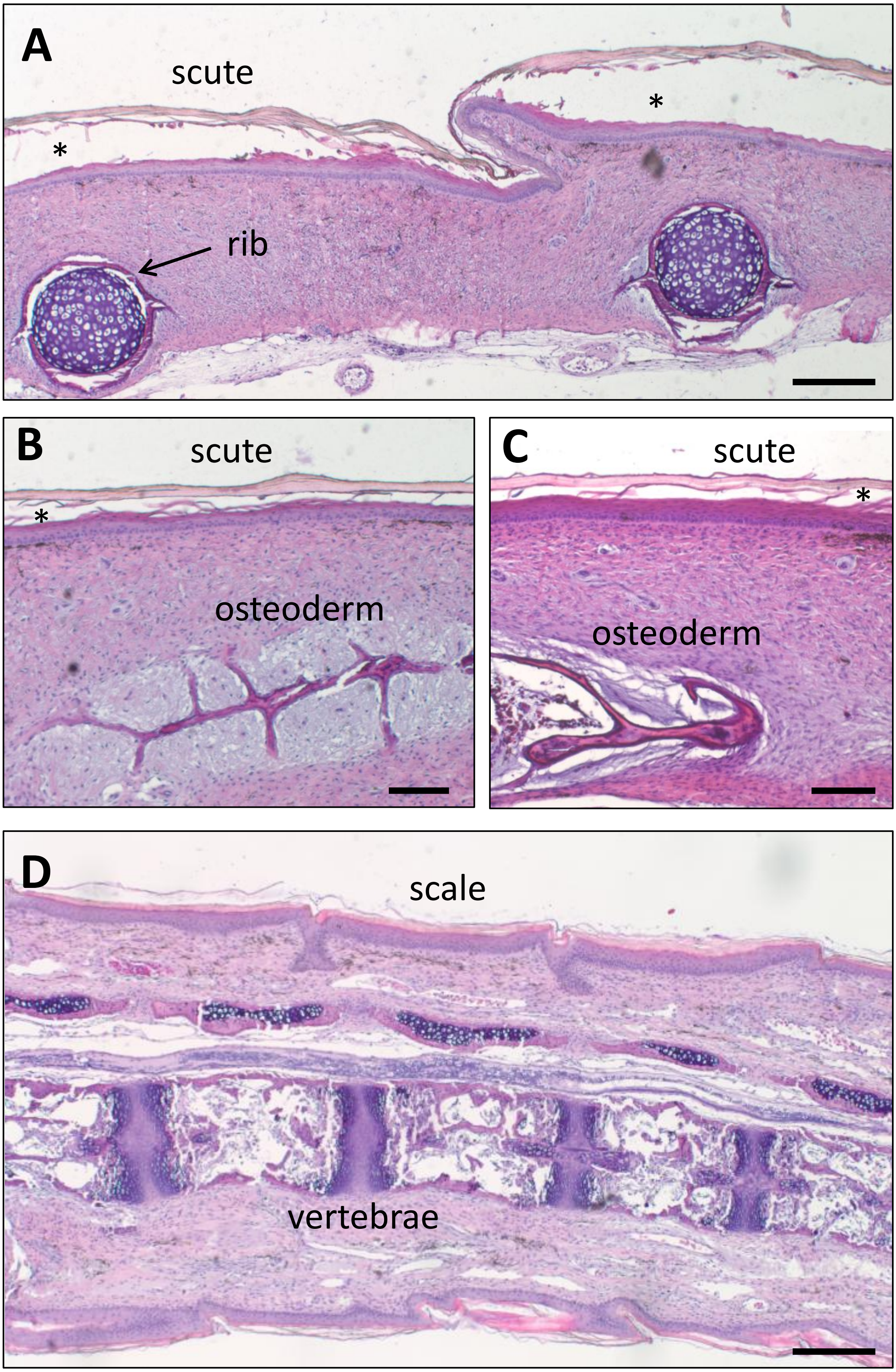

Supplementary Figure S18. Hematoxylin & eosin (H&E) staining of tissues of the European pond turtle (*E. orbicularis*) on embryonic day 45. Continued on next page.

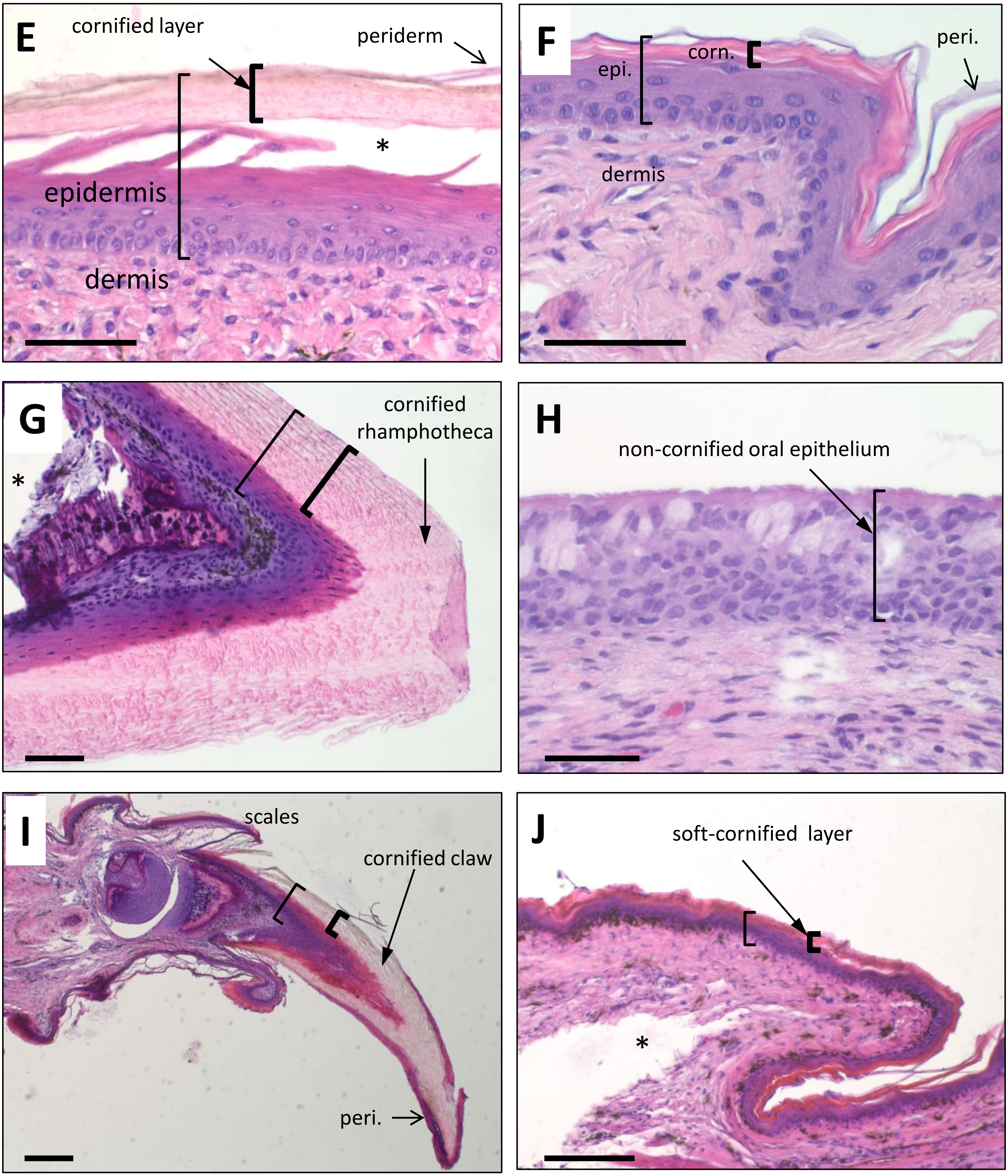

Supplementary Figure S18. Hematoxylin & eosin (H&E) staining of tissues of the European pond turtle (*E. orbicularis*) on embryonic day 45. Tissue samples were taken from embryos of the same developmental stage that was used for RNA preparations (Fig. 4). (A, B, E) Carapace, (C) plastron, (D) tail, (F) leg, (G) rhamphotheca, (H) oral epithelium, (I) toe including claw, (J) neck skin. Asterisks indicate artifacts of tissue preparation. In panels E-J the epidermal compartment is indicated by a thin-lined bracket, and the cornified epidermal compartment is indicated by a thick-lined bracket. Note the presence of the periderm (peri), an embryo-specific layer. Scale bars: (A, D, I) 200 µm; (B,C, J) 100 µm; (E, F, G) 50 µm; (H) 40 µm.

A

>Eo_Beta-A1_partial
GGTTTACCTCCATCACAGAAAGATGTCTTGCTCCAGCCTGTGCTATCCAGAATGCGGGGTGGCCCGNCCCAGTCCAGTT

>Eo_Beta-A4_partial
GGTTTACCTCCATCACAGAAAGATGTCTTGCTCCAGCCTGTGCTATCCAGAATGCGGGGTGGCCCGTCCCAGTCCAGTT

>Eo_Beta-B4_partial
GGCTCANCCCCATTGCAGAAAGATGTTTTCCGATGAGGAGTTCTCTTACAAAAGTCAGCAGCGACATATACCGCAGAAAGGCCAAAACCCATGTCTGCCGCAGAAAG
AGACAAAACCGTGTCCGCCGCAGAAAGTGCCATGTCCGCCAAAGCGTCCTCCGTGTCCAAAGTATCCACCATGCATTCCGTATCCACGGCCATGCCCACCTCAGTAT
CCATCAATATGCCCCAACCATATATTGGGGTCTGGAACGAACCGTGTGTCACAGAATGCGGTGACTCGACTGCAGTCGTCTTTGCACCACTGGTTGTCGTGAATTTC
CCAGGCCCAACTCTTGCCACTTGCCCACAGGATAGCGTAGTGGGATCCTCTCTACCAAGAGGAATTATAGGCCCATATGGTTCCGGGGGCTCTTTCGGTAACGGGGG
CTTATCCTTCAGCAACGGGAGCTCATCCTTCGGTACCGGGAGCTCATTCTTCGGCTCCTGGGGT

>Eo_Beta-B19_partial
GGNTATACCACAATCACAAAAGATGTCTTCCAGCAAAGCTTTGTGTTACCCACGCCCGCCATGTTATCCCGACATCTGCCCCGACCCATATGTTGATGCCTGCAACG
AGCCTTGTGTCACATCATGTGGTGACTCGAGCGCAGTCGTCTATGCCCCACCGGTTCTCGTGAGATTCCCGGGACCAATTCTCGCTACCTGCCCTCAAGACAGC

>Eo_Beta-B32_partial
GGATTCCCTCCATCACAGAAGATGWCTTACTGCCCACCCCAGGATTGTTATCCCGATATATGCCCACGTCCATGTATTGACGTCCGCAACGAGCCGTGTATCTCATC
ATGYGGAGATTCGACTGCAGTG

>Eo_Beta-O17_partial
GGTTTACCTCCATCCCAGAAAGATGACTTTCTCCAGCCTGTGCTATCCAGAATGCGGGGTGGCCCGGCCCTGTCCAGTCACTGGNACCTGCAACGAGCCGTGCGTTA
GGCAGTGCCAGGACTCCGAAGTGGTGATCAGACCCTCACCGGTTGTC

>Eo_EDAA8_partial
TTGGTGAACGGGGTTTACCTCCAAGGCAGAAAGATGACTTTCCACCACCAAAAGCTTTCTCACCATTGGGGCTGTGACCCCTGTTGGAAAGGGGGCTGGGGCGGTTA
TGGGGGCCATTACGGCTGCTATCAGCCATGGGGTTATTCTAGACCATACGGATGGGGGTGGGGCCACAATTCCGGTTCCTGCTACTCTTACCCTTACCGATGGGGGG
GTGGTTACGGCTATGGA

>Eo_EDAA19_partial
CTGGGTTTACCGCCYACACAGAAAGATGACTTTCGATGAAAACTTTAGTGATGAATTGTATTATAAGCCCTACCATTATGGAGGCTGGGGTGGTAGAGGATACGGCT
ACTGTAAGCCATGGTGCTACCAAAGGCCATACAAGTGTTGTTGGGGCT

>Eo_EDbeta1_partial
TGTTACCTCCATCTCAGAAAGATGTCGTGCGGGGCAAACTTGTGCATTGATGGGGGCTCAGCGTGCGGTGTGGCACGCCCCAGGCCATGCGCTGACAGCTGCAACCA
ACCATGCGTCACGCAGTGCCCCGACTCGAGAGTGGTGAT

>Eo_EDKM_partial
TCTAACCTCATAAAGGCCATCGCAGATATGATTAACAGCTACCAGAGCAACTCCAGAAAGGGCAGGGAATCAGAGAGGTTCCGGAGGTGTGAATTTAAGAAGTTGGT
CCAGCAGGAGCCGTCTCCTGCCAAGAGATCATCCAGCAATAAATATAAACACACTACGAGCTTGCCAGATTCTGACGCGGAGCTGATGAACAAGAAAGAACTCATCA
CTGCCAATCCCTGTGTGTACTGAAG

>Eo_EDP3_partial
AGGTTTACCTAGATCATTGAAAGATGTCTTCTGATCAGCAACAGTGTAAGCAGACCTGCCCNCCTCCTCCANAATGCCAGGAGAAATGCCCTCCACCATGCAAGGAG
CCCTGCAAGACTCCNAAATGCCAGGAGAAATGCCCTCCGCCATGCAAGGAGCCCTGCCCTCCCAAATGCCCCCCTCCACAG

>Eo_EDPCV_partial
TTTTGCCCNCNTCCCAGGAAGATGGCTTACCAGCAGCAATGCAAACAGACCTGCCTKCCCCCTCCTTGCTGTGTGACCAAGT

>Eo_EDPE_gDNA_partial
CCTGAGCCAATACCATGTTGTCCAGNGAAGCCACCATGCAAGGAACCACCAGTCCCAATTCNTACTCCATGCCCTGAGCCAATACCATGTCCTCANGAGAAGCAACA
GTGCAAGNTGCCACCAGTCCCAGTTCCTCTTCCACACCCTGAGCCAATACCNTGTTCTCCAGAGAAGCCACCATGCAAGGAACCCCCATTCCCANTTCCTACTCCAT
GCCCTGAGCCAATACCTTATTGTCCAGAAAAGCCACCATGCAAGGAACCAAAAGTCCCACNTCCTCTTCCACACCCTGAGCCAATACCATGTNNTCMAGAGAAGCNR
CNNTGCAAGNAGCCACCAGTCCCAGTTCCTCTTCCACGCCCTGAGCCAATACCNTGTTCTCCAGANAAGCCACCATGCAAGGAACCACCAGTCCCAGTTCCTNTTCC
ACACCCTGAGCCAATACCTTATTGTCCAGANAAGCCGCCATGCAAGNAACCACCAGNCCCNGTTNCTCNTCCANGCCCTGAGCCAATACCATGTTNTCCAGAGAAGC
CNCCATGCAAGGAACCACCAKTCCCAGTTCCTCYTCCACACCCTGAGCCAATACCATGTCCTCAACAGAAGCAGCAGAGCAAGTTGCCACCAGTCCCAGTTCCTACT
CCATGCCCTGAGCCAATACCTTGTTCTCCAGAGAAGCCGCCATGCAAGGAACCACCAGCCCCCGTTACTCCTCCATGCCCNGAGCCAGT

>Eo_EDQM1_partial
AGGGGTTACCCAAGCTACAGAAAGATGTGCTCCCGCCAGGAGAAAGACCACTGCCACAAACAAGATACCTGCCACGGGAGCGGAGGAGGGTCATCTTGCCACGGGAG
CGGAGGAGGATCATCTTGCCACGGGAGCGGAGGAGGATCATCTTGCCACGGGAGCGGAGGAGGGTCATCTTGCCACGGGAGCGGAGGAGGGTCATCTTGCCATGGGA
GCGGAGGAGGGTCATCTTGCCACGGGAAACCACAGAAGCCTTGCCAACAGGAGCAGCAACAGCAG

>Eo_EDQM7_partial
GGGTTACCCAAGCTGCAGAAAGATGTGCTCCCGCCAGGAGAAAGACCAGTGCCACAAACAAGACGGCAGCGGAGGATGCCACAGCAGCGGATCCTCTTGCCATAGTA
GTGGAGGATCTTCTTGCCACAGTGGAGGTTCATCTTGCCATGGGAGCGGAGGGTCTTCTTGCCATAGTAGTGGAGGTTCATCTTGTCATGGTGGAGGMTCATCTTGC
CACAGCGGAGGGTCTTGCCATGGGAAACCACAGCAGCACTGCCAGCAGCAGCAGCAGCAGAA

>Eo_EDWM_partial
GCTTCACCTCGGCTGCAAAATGATTTACTCTTCTGGAAGGGAATCCTACTTCAACTTGAACTCCACCTGGTATGACCCTGCAGGTTCCTGGCTGGACACCCGGCGCA
CCCCCTTCACCTATGCTTATAGCACCTGCTGCAGCAGTGGTGGCTGTCCAAGGGGAGGCCATGATAACCGATGCTACGAGTATCGACGATCGGGCTGTGCTGAGAAT
TGCCATGGGTCGTCGGGGTCGTGCCACGGCAGTGGAGGCCACTGCTGTGTCAGGAGGCCATCGTACTTCCATGGATCCTCTGGAGGATGCCACGGCCATGGGCGGTC
GGTCTGTTCTGAGCGGTCATGCCACGGTTCTGGAGCTTCATGCCACGGTTCTGGATCTTCATGCCACGGTTCTGGATCTTCATGCCACAATACCTCTGGAGCATGCC
ACAGTACACCAATTTATGTGAAGC

>Eo_GAPDH_partial
GACAACTATGGCATAGTGGAAGGTCTCATGACCACTGTCCATGCCATCACAGCCACACAGAAGACTGTGGATGGCCCCTCTGGAAAGCTGTGGCGTGATGGCAGAGG
TGCTGCC

>Eo_LOR_partial
AAGGGTTCCCTAGCCTGCCGGAAGATGTGTTCGCATCAAGAGAAACAGGACTGCTATGAGATCCCAGCTCAGGCTGGAGGATGCCACGCCAGTGGTGGAGGATCCTC
GGGCAGTGGCAGCGGAGCTTTGCTGGGCCAGCCCATTCTGGGCTCGTCGAGCTACGGTGTTGGAGGGGGCTCGTCCTACTGCGGCTCCGGGGAGTC

Supplementary Figure S19. Continued on next pages.

B

```
>Eo_Beta-A1_partial
MSCSSLCYPECGVAXPSPV

>Eo_Beta-A4_partial
MSCSSLCYPECGVARPSPV

>Eo_Beta-B4_partial
MFSDEEFSYKSQQRHIPQKGQNPCLPQKETKPCPPQKVPCPPKRPPCPKYPPCIPYPRPCPPQYPSICPNHILGSGTNRVSQNAVTRLQSSLHHWLS

>Eo_Beta-B19_partial
MSSSKALCYPRPPCYPDICPDPYVDACNEPCVTSCGDSSAVVYAPPVLVRFPGPILATCPQDS

>Eo_Beta-B32_partial
MXYCPPQDCYPDICPRPCIDVRNEPCISSCGDSTAV

>Eo_Beta-O17_partial
MTFSSLCYPECGVARPCPVTXTCNEPCVRQCQDSEVVIRPSPVV

>Eo_EDAA8_partial
MTFHHQKLSHHWGCDPCWKGGWGGYGGHYGCYQPWGYSRPYGWGWGHNSGSCYSYPYRWGGGYGYG

>Eo_EDAA19_partial
MTFDENFSDELYYKPYHYGGWGGRGYGYCKPWCYQRPYKCCWG

>Eo_EDbeta1_partial
MSCGANLCIDGGSACGVARPRPCADSCNQPCVTQCPDSRVV

>Eo_EDKM_partial
SNLIKAIADMINSYQSNSRKGRESERFRRCEFKKLVQQEPSPAKRSSSNKYKHTTSLPDSDAELMNKKELITANPCVY

>Eo_EDP3_partial
MSSDQQQCKQTCXPPPXCQEKCPPPCKEPCKTXKCQEKCPPPCKEPCPPKCPPPQ

>Eo_EDPCV_partial
MAYQQQCKQTCXPPPCCVTK

>Eo_EDPE_gDNA_partial
PEPIPCCPXKPPCKEPPVPIXTPCPEPIPCPXEKQQCKXPPVPVPLPHPEPIXCSPEKPPCKEPPFPXPTPCPEPIPYCPEKPPCKEPKVPXPLPHPEPIPCXXEKX
XCKXPPVPVPLPRPEPIXCSPXKPPCKEPPVPVPXPHPEPIPYCPXKPPCKXPPXXVXXPXPEPIPCXPEKXPCKEPPXPVPXPHPEPIPCPQQKQQSKLPPVPVPT
PCPEPIPCSPEKPPCKEPPAPVTPPCXEP

>Eo_EDQM1_partial
MCSRQEKDHCHKQDTCHGSGGGSSCHGSGGGSSCHGSGGGSSCHGSGGGSSCHGSGGGSSCHGSGGGSSCHGKPQKPCQQEQQQQ

>Eo_EDQM7_partial
MCSRQEKDQCHKQDGSGGCHSSGSSCHSSGGSSCHSGGSSCHGSGGSSCHSSGGSSCHGGGSSCHSGGSCHGKPQQHCQQQQQQ

>Eo_EDWM_partial
MIYSSGRESYFNLNSTWYDPAGSWLDTRRTPFTYAYSTCCSSGGCPRGGHDNRCYEYRRSGCAENCHGSSGSCHGSGGHCCVRRPSYFHGSSGGCHGHGRSVCSERS
CHGSGASCHGSGSSCHGSGSSCHNTSGACHSTPIYVK

>Eo_GAPDH_partial
LAKVINDNYGIVEGLMTTVHAITATQKTVDGPSGKLWRDGRGAAQNIIPA

>Eo_LOR_partial
MCSHQEKQDCYEIPAQAGGCHASGGGSSGSGSGALLGQPILGSSSYGVGGGSSYCGSGE
```

Supplementary Figure S19. Continued on next page.

C

```
Cp_EDbeta1                                                   MSCGANLCIDGGSACGVARPRPCADSCNQPCVTQCPDSRVIIYPPPVVVTFPGPILTTFPQESVVESVGA
Eo_EDbeta1                                                   MSCGANLCIDGGSACGVARPRPCADSCNQPCVTQCPDSRVV
Cp_EDbeta2                                                   MSCSRNVCTAGGSACGVARPRPFTDSCNQPCVTRCPDSRVIIYPPPVVVTFPGPILTTFPQESVVESVGA

Cp_Beta-A1                                                       MSCSSLCYPECGVARPSPVSGSCNEPCVRQCPDSEVIIRPSPVVVTIPGPILSNFPQQSEVGAVGA
Eo_Beta-A1**                                                     MSCSSLCYPECGVAXPSPV
Cp_Beta-A2                                                       MSCSSLCYPECGVARPSPVSGSCNEPCVRQCPDSEVIIRPSPVVVTIPGPILSNFPQQSEVGAVGA
Cp_Beta-A6                                                       MSCSSLCYPECGVARPSPVSGSCNEPCVRQCPDSEVIIRPSPVVVTIPGPILSNFPQQSEVGAVGA
Cp_Beta-A7                                                       MSCSSLCYPECGVARPSPVSGSCNEPCVRQCPDSEVIIRPSPVVVTIPGPILSNFPQQSEVGAVGA
Cp_Beta-A4                                                       MSCSSLCYPECGVARPSPVSGSCNEPCVRQCPDSEVIIRPSPVVVTIPGPILSNFPQQSEVGAVGA
Eo_Beta-A4**                                                     MSCSSLCYPECGVARPSPV
Cp_Beta-A5                                                       MSCSSLCYPECGVARPSPVSGSCNEPCVRQCPDSEVIIRPSPVVVTIPGPILSNFPQQSEVGAVGA
Cp_Beta-A9                                                       MSCSSLCYPECGVARPSPVSGSCNEPCVRQCPDSEVIIRPSPVVVTIPGPILSNFPQQSEVGAVGA
Cp_Beta-A1L1                                                     MSCSSLSYPECGVARPSPVSGTCNEPCIRQCPDSEVVIRPSPVVVTLPGPILSTFPQQSEVAAVGA
Cp_Beta-A1L2                                                     MSCSSLSYPECGVARPSPVSGSCNEPCVRQCPDSEVVIRPSPVVVTLPGPILSTFPQQSGVGAVGA
Cp_Beta-A10                                                      MSCSSMCYPECGVTRPSPVSGSCNEPCVRQCPDSEVIIRPSPVVVTIPGPILSNFPQQSRVGAIGA
Cp_Beta-A3                                                       MSCSSLCYPECGVARTSPDSGSCNELCVRQCPDSEVIIRPSPVVVTIPGPILSNFPYRGHYGRLYC
Cp_Beta-A8                                                       MSCSSRCYPECGVARPSPVSGSCNELCVRQCPDSEVIIRPSPVVVTIPGQILSNFPYGGHYGRLYG

Cp_Beta-O3                                                       MTFSSLCYPECGVARPSPVTGSANEPCVRQCPDSEVVIRPSPVVVTLPGPILSNFPQQSEVAAVGA
Cp_Beta-O1                                                       MTFSSLCYPECGVARPSPVTGSSNEPCVRQCPDSEVVIRPSPVVVTLPGPILSNFPQQSEVAAVGA
Cp_Beta-O4                                                       MTFSSLCYPECGVARPSPITGSSNEPCVRQCPDSEVVIRPSPVVVTLPGPILSNFPQESEVAAVGA
Cp_Beta-O2                                                       MTFSSLCYPECGVARPSPVTGTCNEPCVRQCQDSEVVIRPSPVVVTLPGPILSNFPQQSEVAAVGA
Cp_Beta-O5                                                       MTFSSLCYPECGVARPSPVTGSSNEPCVRQCPDSEVVIRPSPVVVTLPGPILSNFPQESEVAAVGA
Cp_Beta-O17                                                      MTFSSLCYPECGVARPCPVTGTCNEPCVRQCQDSEVVIRPSPVVVTLPGPILSNFPQHSGVGALGA
Eo_Beta-O17                                                      MTFSSLCYPECGVARPCPVTXTCNEPCVRQCQDSEVVIRPSPVV
Cp_Beta-O22                                                      MTFSSLCYPECGVARPCPVTGTCNEPCVRQCQDSEVVIRPSPVVVTLPGPILSNFPQHSAVGAVGA
Cp_Beta-O23                                                      MTFSSLCYPECGVARPCPVTGTCNEPCVRQCQDSEVVIRPSPVVVTLPGPIMSNFPQHSGVGAVGA
Cp_Beta-O24                                                      MTFSSLCYPECGVARPCPVTGTCNEPCVRQCQDSEVVIRPSPVVVTLPGPIMSNFPQHSGVGAVGA
Cp_Beta-O25                                                      MTFSSLCYPECGVARPCPVTGTCNEPCVRQCQDSEVVIRPSPVVVTLPGPIMSNFPQHSGVGAVGA
Cp_Beta-O20                                                      MTFSSLCYPECGVARPCPVTGTCNEPCVRQCPDSEVVIRPSPVVVTLPGPILSNFPQHSAVGAVGA
Cp_Beta-O21                                                      MTFSSLCYPECGVARPCPVTGTCNEPCVRQCPDSEVVIRPSPVVVTLPGPIMSNFPQHSGVGAVGA
Cp_Beta-O26                                                      MTFSSLCYPECGVARPCPVTGTCNEPCVRQCPDSEVVIRPSPVVVTLPGPIMSNFPQHSGVGAVGA
Cp_Beta-O27                                                      MTFSSLCYPECGVARPCPVTGTCNEPCVRQCQDSEVVIRPSPVVVTLPGPIMSNFPQHSGVGAVGA
Cp_Beta-O9                                                       MTFSSLCYPECGVARPSPVTGTCNEPCVRQCQDSEVVIRPSPVVVTLPGPIMSNFPQHSGVGAIGA
Cp_Beta-O10                                                      MTFSSLCYPECGVARPSPVTGTCNEPCVRQCQDSEVVIRPSPVVVTLPGPIMSNFPQHSGVGAVGA
Cp_Beta-O11                                                      MTFSSLCYPECGVARPSPVTGTCNEPCVRQCQDSEVVIRPSPVVVTLPGPIMSNFPQHSGVGAVGA
Cp_Beta-O12                                                      MTFSSLCYPECGVARPSPVTGTCNEPCVRQCQDSEVVIRPSPVVVTLPGPIMSNFPQHSGVGAVGA
Cp_Beta-O13                                                      MTFSSLCYPECGVARPSPVTGTCNEPCVRQCQDSEVVIRPSPVVVTLPGPIMSNFPQHSGVGAVGA
Cp_Beta-O14                                                      MTFSSLCYPECGVARPSPVTGTCNEPCVRQCQDSEVVIRPSPVVVTLPGPIMSNFPQHSGVGAVGA
Cp_Beta-O15                                                      MTFSSLCYPECGVARPSPVTGTCNEPCVRQCQDSEVVIRPSPVVVTLPGPIMSNFPQHSGVGAVGA
Cp_Beta-O16                                                      MTFSSLCYPECGVARPSPVTGTCNEPCVRQCQDSEVVIRPSPVVVTLPGPIMSNFPQHSGVGAVGA
Cp_Beta-O28                                                      MISSSLCYPECGVARPCPVTGTCNEPCVRQCQDSEVVIRPSPVVVTLPGPILSNFPQHSAVGALGA
Cp_Beta-O29                                                      MISSSLCYPECGVARPCPVTGTCNEPCVRQCQDSEVVIRPSPVVVTLPGPILSNFPQHSAVGAVGA
Cp_Beta-O18                                                      MTFSSLCYPECGVARPCPVTGTCNEPCVRQCQDSEVVIRPSPVVVTLPGPILSNFPQHSAVGALGA
Cp_Beta-O19                                                      MTFSSLCYPECGVAQPCPVTGTCNEPCVRQCQDSEVVIRPSPVVVTLPGPIMSHYTQESVVGALGA
Cp_Beta-O6                                                       MTFSSLCYPECGVARPSPVTGSCNEPCVRQCQDSQVVIRPSPVVVTLPGPIMSNFPQHSAVGAVGA
Cp_Beta-O7                                                       MTFSSLCYPECGVARPSPVTGSCNEPCVRQCQDSQVVIRPSPVVVTLPGPIMSNFPQHSAVGTVGA
Cp_Beta-O8                                                       MTFSSLCYPECGVAQPSPVTGSCNEPCVRQCPDSQVVIRPSPVVMTLPGPILSNFPQHSVVGAVGA
Cp_Beta-B2                                                       MSCYGLRNIPCEVPRPTPAAVTYNEPCVIQCPDSIFESDSPPGIAIIPGPILTTFPHYSVVETSPL
Cp_Beta-B1                                                  MSFNGPQTGAQGSLPCGVKCSEPYIATASEPCVVKCKDSRVIIYPPPVVVTFPGPILTTCPQESIVASSGP
Cp_Beta-B36                                                 MSFNGVPCNDQCHNPCEVTCPQPIVNSSNQPCVVSCGDSRVVIYPPPVVVTFPGPILSTCPQDSIVGSSAA
Cp_Beta-B10                                                 MSSHRQLVSPRCATPWEVTCPQPGANICSQPCVTSCEDSRVMVYAPPVVVAFPGPILSTCPQKSITGSEVP
Cp_Beta-B6                                                  MSSYRQLCNTQCYAPCNVTCPRPFVDACNEPCFTSCGDSSAVLYPPPVIVRFPGPILATCPQESVVGSSAP
Cp_Beta-B7                                                  MSSYRQLCNTQCYAPCNVTCPRPFVDACNEPCFTSCGDSSAVLYPPPVIVRFPGPILATCPQESVVGSSAP
Cp_Beta-B8                                                  MSFCRDLCKYPSYPSCDVTCPQPFVDACNQPCVTSCGDSSVVVYPPPVVVRFPGPILATCPQESVVGSSEP
Cp_Beta-B3  MFSDEEFFYKNKQPQKPQKGQNPCLPQKKPKPCPPQKVPCTPKPPRCPTYPPYIPPPRPCPPQYPSI-CPQPYIGVWNEPCVTECGDSTAVVFAPPVVVNFPGPTLATCPQDSVVGSSLP
Cp_Beta-B4  MFSDEEFWYKSQQPQKPQKGQNPCLPQKKPKPCPPQKVPCTPKPPRCPTYPPYIPPPRPCPPQYPSI-WPQPYIGVWNEPCVTECGDSTAVVFAPPVVVNFPGPTLATCPQDSVVGSSLP
Eo_Beta-B4* MFSDEEFSYKSQQRHIPQKGQNPCLPQKETKPCPPQKVPCPPKRPPCPKYPPCIPYPRPCPPQYPSI-CPNHILGSGTNRVSQNAVTRLQSSLHHWLS
Cp_Beta-B14                                                 MSSSKDLCYPRPPCYPDI-CPNPYVDAWNEPCVTSCGDSSAVVYAPPVVVRFPGPILATCPQDSVVGSTLP
Cp_Beta-B19                                                 MSSSKALCYPRPPCYPDI-CPDPYVDAWNEPCVTSCGDSSAVVYAPPVVVRFPGPILATCPQDSIVGSTLP
Eo_Beta-B19                                                 MSSSKALCYPRPPCYPDI-CPDPYVDACNEPCVTSCGDSSAVVYAPPVLVRFPGPILATCPQDS
Cp_Beta-B21                                                 MSSSKALCYPRPPCYPDI-CPDPYVDAWNEPCVTSCGDSSAVVYAPPVVVRFPGPILATCPQDSVVGSTLP
Cp_Beta-B15                                                 MSSSKALCYPRPPCYPDI-CPDPYVDACNEPCVTSCGDSSAVVYAPPVVVRFPGPILATCPQDSVVGSTLP
Cp_Beta-B16                                                 MSSSKALCYPRPPCYPDI-CPDPYVDACNEPCVTSCGDSSAVVYAPPVVVRFPGPILATCPQDSVVGSTLP
Cp_Beta-B17L                                                MSSSKALCYPRPPCYPDI-CPDPYVDACNEPCVTSCGDSSAVVYAPPVVVRFPGPILATCPQDSVVGSTLP
Cp_Beta-B17                                                 MSSSKALCYPRPPCYPDI-CPDPYVDACNEPCVTSCGDSSAVVYAPPVVVRFPGPILATCPQDSVVGSTLP
Cp_Beta-B20                                                 MSSSKALCYPRPPCYPDI-CPNPYVDACNEPCVTSCGDSSAVVYAPPVVVRFPGPILATCPQDSVVGSTLP
Cp_Beta-B18L                                                MSSSKALCYPRPPCYPDI-CPNPYVDAWNEPCVTSCGDSSAVVYAPPVVVRFPGPILATCPQDSVVGSTLP
Cp_Beta-B18                                                 MSSSKDLCYPRPSCYPDI-CPNPYVDAWNEPCVTSCGDSSAVVYAPPVVVRFPGPILSTCPQDSVVGSTLP
Cp_Beta-B22                                                 MSSSKALCYPRPPCYPDI-CPDPYVDAWNEPCVTSCGDSSAVVYAPPVVVRFPGPILATCPQDSVVGSTLP
Cp_Beta-B23                                                 MSSSKALCYPRLPCHPDI-CPNPYVDAWNESCVTSCGDSSAVVYAPPVVVRFPGPILATCPQDSVVGSTLP
Cp_Beta-B11                                                 MSSCKDLSCRPSPCYPDI-CPDPCVVARNEPCITSCADSTAVVYPPPVSVLFPGPILSSSPQHSLVGSTLP
Cp_Beta-B12                                                 MSSCKDLSCRPSPCYPDI-CPDPCVVARNEPCITSCADSTAVVYPPPVSVLFPGPILSSSPQHSVVGSTLP
Cp_Beta-B13                                                 MSSCKDLSCRPSPCYPDI-CPDPCVVARNEPCITSCADSTAVVYPPPVSVLFPGPILSSCPQHSLVGSTLP
Cp_Beta-B28                                                      MSCCPPQDCIPDI-CPCPYIDVRNEPCISSCGDSTAVVYAPPVVVNFPGPTMATCPQDSFIGTSLP
Cp_Beta-B29                                                      MSYCPPQDCIPDI-CPRPYIDVCNEPCISSCGDSNAVVFAPPVVVRFPGPTMATCPQDSIVGSSLP
Cp_Beta-B30                                                      MSYCPPQDCIPDI-CPRPYIDVCNEPCISSCGDSNAVVFAPPVVVRFPGPTMATCPQDSIVGSSLP
Cp_Beta-B31                                                      MSYCPPQDCIPDI-CPRPYIDVCNEPCISSCGDSTAVVFAPPVVVRFPGPTMATCPQDSFVGSSLP
Cp_Beta-B32                                                      MSYCPPQDCYPDI-CPRPCIDVRNEPCISSCGDSTAVVYAPPVVVRFPGPTMATCPQDSFVGSSLP
Eo_Beta-B32                                                      MXYCPPQDCYPDI-CPRPCIDVRNEPCISSCGDSTAV
Cp_Beta-B33                                                      MSYCPPQDCYPDI-CPRPCIDVRNEPCISSCGDSTAVVYAPPVVVRFPGPTMATCPQDSFVGSSLP
Cp_Beta-B34                                                      MTYCPPQDCYPDI-CPRPCIDVRNEPCISSCGDSTAVVFAPPVVVRFPGPTMATCPQDSFVGSSLP
Cp_Beta-B35                                                      MSYCPPQDCYPDI-CPRPCIDVRNEPCISSCGDSTAVVFAPPVVVRFPGPTMATCPQDSFVGTSLP
Cp_Beta-B24                                                  MFAEFLCWQPRPYCPDI-CPDPCAYVCNKPFVTSCGDSNGVVYAPPDVMRFPGQALTTCPQDSFVGTVGP
Cp_Beta-B25                                                 MSPVKDLCCQPGPYYPDI-CPDPCAYVCNEPCVTTCGDSNAVVFAPPVVVRFPGPTLATCPQDSFVGTSLP
Cp_Beta-B26                                                 MSPVKDLCYQPRPYCPDI-CPEPCAYVCNEPCVTTCGDSNAVVFAPPVVVRFPGPTLATCPQDSFVGTSLP
Cp_Beta-B27                                                 MSPVKDLCCQPRPYCPDI-CPDPCAYVCNEPCVTSCGDSSAVVFAPPVVVRFPGPTLATCPQDSFVGTSLP
Cp_Beta-B5                                                  MSSYEQLCNTQCYAPCNVTCPQPIVDTCNEPCITSCSDSRAVVYPPLIVVTFPGTLLSFCPQESVEESSAH
Cp_Beta-B9                                                  MSFYGDPARSQCYLPCEGTCQQPVANVCNEPWVRSGGDSRGVGYAPLVVVTFPGPSSQYLLSGKHDWNGTA
```

Supplementary Figure S19. EDC genes identified by sequencing cDNAs of *E. orbicularis*. (A) Nucleotide sequences of cDNAs from EDC-related genes of *E. orbicularis* (Eo). The cDNAs were amplified from various embryonic tissues (day 45) of *E. orbicularis* (see main text) and sequenced. In addition, the partial sequence of EDPE, amplified from genomic DNA (gDNA) is included. (B) Amino acid sequences of EDC-proteins of *E. orbicularis* (Eo). Amino acid residues are highlighted as in Suppl. Fig. S1. (C) Alignment of partial (N-terminal) amino acid sequences of beta-keratins from *E. orbicularis* (Eo) and *C. picta* (Cp). Eo sequences (highlighted by yellow shading) are inserted next to the most similar Cp sequence in which identical amino acid residues are also highlighted. Conserved residues characteristic for individual beta-keratins or clusters of beta-keratins in *C. picta* are highlighted by green shading. *The cDNA of Eo_Beta-B4 contained a frame shift (italics indicate residues downstream of the frameshift). **, annotation based on nucleotide sequence alignment (not shown).

Beta A

```
Pn_Beta-3     MSC-----SSLCYPECGVARPSPVSGSCNEPCVRQCPDSEVIIRPSPVVVTIPGPILSNFPQQSEVGAVGAPVVGAGYGGSFGLGGLYGYGGHYGGLYGLGGLGGYGGRYGYGGGYGG----LCGYGGRYGYGGLSGYGGRYGGLCGYGGGYGGGYGYGGACGSGVSCHR--YLSGSCTPC
Cp_Beta-A2    MSC-----SSLCYPECGVARPSPVSGSCNEPCVRQCPDSEVIIRPSPVVVTIPGPILSNFPQQSEVGAVGAPVVGAGYGGSFGLGGLYGYGGHYGGLYGYGGLGGYGGRYGYGGGYGG----LCGYGGRYGYGGLSGYGGRYGGLCGYGGGYGGGYGYGGACGSGVSCHR--YLSGSCTPC
Pn_Beta-5     MSC-----SSLCYPECGVARPSPVSGSCNEPCVRQCPDSEVIIRPSPVVVTIPGPILSNFPQQSEVGAVGAPVVGAGYGGSFGLGGLYGYGGHYGGLYGYGGLGGYGGRYGYGGGYGGGYGGLCGYGGRYGYGGLSGYGGRYGGLCGYGGGYGGGYGYGGACGSGVSCHR--YLSGSCTPC
Cp_Beta-A6    MSC-----SSLCYPECGVARPSPVSGSCNEPCVRQCPDSEVIIRPSPVVVTIPGPILSNFPQQSEVGAVGAPVVGAGYGGSFGLGGLYGYGGHYGGLYGYGGLGGYGGRYGYGGGYGGGYGGLCGYGGRYGYGGLSGYGGRYGGLCGYGGGYGGGYGYGGACGSGVSCHR--YLSGSCTPC
```

Beta O

```
Pn_Beta-1     MTF-----SSLCYPECGVARPSPVTGSSNEPCVRQCQDSQVVINPSPVVMTLPGPILSNFPQHSVVGAVGAPVVGAGFGGSYGLGGLNGSGGHYGGLSGLGGYGGY----------------------------------------------------GGLCGSGVSCHR--YLSGSCGPC
Cp_Beta-07    MTF-----SSLCYPECGVARPSPVTGSCNEPCVRQCQDSQVVIRPSPVVVTLPGPIMSNFPQHSAVGTVGAPVVGAGFGGSYGLGGLNGSGGQYGGLSGLGGYGGY----------------------------------------------------GGLCGSGVSCHR--YLSGSCGPC
Pn_Beta-2     MIS-----SSLCYPERGVARPCPVTGTCNEPCVRQCPDSEVVIQPSPVVVTIPGPILSNFPQHSAVGAVGAPVVGPGFGGSFGHGG-YGYGGLYGGLYGLGGYGGYGGRYGYGGLWG------------------------HGGYCGYPGLYG----YGGLWGYGGYGRR--YLGGRCGTC
Pn_Beta-7     MIS-----SSLCYPECGVARPCPVTGTCNEPCVRQCPDSEVVIQPSPVVVTIPGSILSNFPQHSAVGALGAPVVGPGFGGSFGYGG-YGYGGLYGGLYGLGGYGGYGGHYGYGGLWG------------------------HGGYCGYPGLYG----YGGLWGYGGYGRR--YLGGRCGTC
Pn_Beta-9     MIS-----SSLCYPECGVARPCPVTGTCNEPCVRQCQDSEVVISPSPVVVTPPGPILSNFPQHSAVGAVGAPVVGPGFGGSFGHGG-YGYGGLYGGLYGLGGYGGYGGHYGYGGLWG------------------------HGGYCGYPGLYG----YGGLWGYGGYGRR--YLGGRCGTC
Pn_Beta-6     MTF-----SSLCYPECGVARPCPVTGTCNEPCVRQCPDSEVVIRPSPVVVTLPGPILSNFPQHSGVGAVGAPVVGPGFGGSFGHGG-YGYGGLYGGLYGLGGYGGYGGHYGYGGLWG------------------------HGGYCGYPGLYG----YGGLWGYGGYGRR--YLGGRCGTC
Cp_Beta-025   MTF-----SSLCYPECGVARPCPVTGTCNEPCVRQCQDSEVVIRPSPVVVTLPGPIMSNFPQHSGVGAVGAPVVGPGFGGSFGHGG-FGYGGLYGGLYGLGGYGGYGGHCGYGGLWG------------------------HGGYCGYPGLYG----YGGLWGYGGYGRR--YLGGRCGTC
Pn_Beta-8     MTF-----SSLCYPECGVARPCPVTGTCNEPCVRQCPDSEVVIRPSPVVVTLPGPILSNFPQHSAVGAVGAPVVGPGFGGSFGHGG-YGYGGLYGGLYGLGGYGGYGGHYGYGGLWG------------------------HGGYCGYPGLYG----YGGLWGYGGYGRR--YLGGHCWPC
Pn_Beta-10    MTF-----SSLCYPECGVARPCPVTGTCNEPCVRQCPDSEVVIRPSPVVVTLPGPILSNFPQHSGVGAVGAPVVGPGFGGSFGHGG-YGYGGLYGGLYGLGGYGGYGGRYGYGGLWG------------------------HGGYCGYPGLYG----YGGLWGYGGYGRR--YLGGHCWPC
Cp_Beta-017   MTF-----SSLCYPECGVARPCPVTGTCNEPCVRQCQDSEVVIRPSPVVVTLPGPILSNFPQHSGVGALGAPVVGPGFGGSFGHGG-FGYGGLYGGLYGLGGYGGYGGRYGYGGLWG------------------------HGGYCGYPGLYG----YGGLLGYGGYGRR--YLGGRCGPC
Pn_Beta-11    MTF-----SSLCYPECGVARPSPVTGTCNEPCVRQCPDSEVVIRPSPVVVTLPGPILSNFPQHSGVGAVGAPVVGPGFGGSFGHGG-YGYGGLYGGLYGLGRLGGYGGRYGYGGLLG------------------------YGGHCGYPGLYG----YGGLWGYGGYGRR--YLSGYCGPC
Pn_Beta-15    MTF-----SSLWYPECGVARPSPVTGSSNEPCVRQCPDSEVVIRPSPVVVTLPGPILSNFPQESEVAAIGAPVVGAGFGGSFGLGGLYGYGGHNGGLYGLGRLGGYRGLYGYGGLLG------------------------HGGYCGYPGLYG----YGGLWGYGGYGRR--YLGGYCGPC
Cp_Beta-05    MTF-----SSLCYPECGVARPSPVTGSSNEPCVRQCPDSEVVIRPSPVVVTLPGPILSNFPQESEVAAVGAPVVGAGFGGSFGLGGLYGYGGHYGGLYGLGRLGGYRGLYGYRGLLG------------------------HGGYCGYPGLYG----YGGLWGYGGYGRR--YLGGYCGPC
Pn_Beta-16    MTF-----SSLCYPECGMARPSPVTGSSNEPCVRQCPDSEVVIRPSPVVVTFPGPILSNFPQQSKVAAVGAPVVGAGFGGSFSLGRLYGYGGHYGGLHGLGGYGGYGVLYGYGGLLG------------------------YGGHCGYPGLYG----YGGLWGYGGYGCR--HLDGYCGSC
Pn_Beta-4     MTF-----SSLCYPECGVARPSPVTGTCNEPCVRQCQDSEVVIRPSPVVVTLPGPIMSNFPQHSGVGAVGAPVVGPGFGGSFGHGG-LGYGGLYGGLYGLGGYGGYGGHYGYGGLGG------------------------Y--L----GGYG----YGGLCGSGVSCHR--YLSGSCGPC
Cp_Beta-012   MTF-----SSLCYPECGVARPSPVTGTCNEPCVRQCQDSEVVIRPSPVVVTLPGPIMSNFPQHSGVGAVGAPVVGPGFGGSFGHGG-LGYGGLYGGLYGLGGYGGYGGHYGYGGLGG------------------------Y--L----GGYG----YGGLCGSGVSCHR--YLSGNCGPC
Cp_Beta-09    MTF-----SSLCYPECGVARPSPVTGTCNEPCVRQCQDSEVVIRPSPVVVTLPGPIMSNFPQHSGVGAIGAPVVGPGFGGSFGHGG-FGYGGLYGGLHGLGGYGGYGGHYGYGGLGG------------------------Y--L----GGYG----YGGLCGSGVSCHR--YLSGNCGPC
Pn_Beta-14    MTF-----SSLCYPECGVARPCPVTGTCNEPCVRQCQDSEVVIRPSPVVVTLPGPIMSNFPQHSGVGAVGAPVVGPGFGGSFGHGG-YGYGGLYGGLYGLGGYGGYGGHYGYGGLGG------------------------Y--L----GGYG----YGGLCGSGVSCHR--YLSGNCGPC
Cp_Beta-014   MTF-----SSLCYPECGVARPSPVTGTCNEPCVRQCQDSEVVIRPSPVVVTLPGPIMSNFPQHSGVGAVGAPVVGPGFGGSFGRGG-FGYGGLYGGLYGLGGYGGYGGHYGYGGLGG------------------------Y--L----GGYG----YGGLCGSGLSCHR--YLSGNCGPC
Pn_Beta-12    MIS-----SSLCYPECGVARPCPVTGTCNEPCVRQCQDSEVVIRPSPVVVTLPGPILSNFPQHSAVGALGAPVVGPGFGGSFGHGG-FGYGGLYGGLYGLGGYGGYGGRYGYGGLYG------------------------FGGL----GGYG----YGGLCGSGVSCHR--YLSGNCGPC
Cp_Beta-029   MIS-----SSLCYPECGVARPCPVTGTCNEPCVRQCQDSEVVIRPSPVVVTLPGPILSNFPQHSAVGAVGAPVVGPGFGGSFGHGG-FGYGGLYGGLYGLGGYGGYGGRYGYGGLYG------------------------FGGL----GGYG----YGGLCGSRLSCHR--YLSGNCGPC
Pn_Beta-13    MTF-----SSLCYPECGVARPCPVTGTRNEPCVRQCPDSEVVIRPSPVVVTLPGPILSNFPQHSAVGAVGAPVVGPGFGGSFGHGG-YGYGGLYGGLYGLGGYGGYGGRYGYGGLYG------------------------FGGL----GGYG----YGGLCGSGVSCHR--YLSGNCGPC
Cp_Beta-018   MTF-----SSLCYPECGVARPCPVTGTCNEPCVRQCQDSEVVIRPSPVVVTLPGPILSNFPQHSAVGALGAPVVGPGFGGSFGHGALYGYGGLYGGWCGLGGYGGYCGPYGYGGLGG------------------------Y--L----GCYG----YGGICGSGVSCHR--YLSGSCGPC
```

Beta B

```
As_Beta-1     M---KSLCPPRCHPYPDI-CPEPCARVCNEPCVTSCGDSTAVVYAPPVAVRFPGPILATCPQESIVGS-S---EPLGIGSATGYGGSNLSVSSYGYRPSLGYGGSSGSQSLNSFR--------------------------RSYTSGVSSVSRGGSDPCSSRWLMMYG----CGPRPTQQH
Ps_Beta-16    MSFCRDLCPSPSYPACQVTCPQPFVDACNGPCVTSCGDSTAVVYPPPVIVNFPGPILATCPQESIVGS-S---EPLGIGSAIGYGGSNLSVSSYGYRPSLGYGGSSGSQSLNSLR--------------------------RSYTSGVSSVGRGGSDPCSSRWLMMYG----CGPRPTQQH
Cp_Beta-B8    MSFCRDLCKYPSYPSCDVTCPQPFVDACNQPCVTSCGDSSVVVYPPPVVVRFPGPILATCPQESVVGS-S---EPLGIGSSFGYRGSYLSGSSYGYK------------SLYNDR--------------------------RSYTPGLSSLGRGSSDPCSSRWLNMYG----CGPRQTQQE
As_Beta-2     MNS---LCAPRCNPCP-----EPCAYVCNEPCVTSCGDSTAVVYAPPVAIRFPGPILATCPQDSVVGS-SLPQIPYGPYGPYGGGARSGAGSILGGGGSGVFGGGSGGGAGWVGGSGHG----------------------YSYGSNYGSSSGGY-GRHCSYTCVPCP--------RYRPC
Ps_Beta-56    MKF---PCAPRCYPCPDI-CPEPCAYVCNEPCVTSCGDSTAVVYAPPVAVRFPGPILATCPQDSVVGT-TLP-LP--PYGPYRGGAGGGAGSFLGGGGSGVFGGGSGGGAGGLGGLGGG----------------------YCYGS----SSGGY-GRHCSYTCVPCP--------RYRPC
As_Beta-3     MKS---LCPPRCHPYPDI-CPEPCACVCNEPCVTSCGDSTAVVYAPPVAVRFPGPILATCPQDSVVGS-SLPQIPYGPYGPYGGGAGGGGVLGGAGGVGSGALVGAGGGSWSAFGGGSG----------------------RGYNGGFGGSHGGYWGRRCYANRYDCC-----------PW
Ps_Beta-58    MKF---PCAPRCHPCPDI-CPEPCAYVCNEPCVTSCGDSTAVVYAPPVAIRFPGPILATCPQDSVVGS-SLPQIPYGPYG---GGAGGGGALGGAGGLGGGALVGAGSG----FGGGSG----------------------GGFGSGFGGCHGGYWGRRCYANRYDCC-----------PW
As_Beta-4     MA------CVPQDCYSDI-CPRPYIDVCNSPCISSCGDSTAVVFAPPVVVRFPGPTMATCPQDSFVGS-SLPQLPAGSGGYPGVGGGVSGSLGSG-GYGGVSGGRFGGSSVGGFGGNYG-----GYSSGYGGGYA------GGCGGGYSGGNGGSCGSRRSYRSISACGGGYSSKGSCGPC
As_Beta-5     MA------CVPQDCYSDI-CPRPYIDVCNSPCISSCGDSTAVVFAPPVVVRFPGPTMATCPQDSFVGS-SLPQLPAGSGGYPGVGGGISGSLGSG-GYGGVYGGSFGGSSVGGFGGNFG-----GYR----GGYA------GGCGGGYSGGNGGSCGTRRSYRSISACGGGYSSKGSCGPC
As_Beta-7     MA------CVPQDCYSDI-CPRPYIDVCNSPCISSCGDSTAVVFAPPVVVRFPGPTMATCPQDSFVGS-SLPQLPAGSGGYPGVGGGISGSLGSG-GYGGVYGGSFGGSSVGGFGGNFG-----GYR----GGYA------GGCGGGYSGGNGGSCGTRRSYRSISSCGGGYSSKGSCGPC
As_Beta-9     MA------CVPQDCYSDI-CPRPYIDVCNSPCISSCGDSTAVVFAPPVVVRFPGPTMVTCPQDSFVGS-SLPQLPAGSGGYPGVGGGISGSLGSG-GYGGVYGGSFGGSSVGGFGGNFG-----GYR----GGYA------GGCGGGYSGGNGGSCGSRRSYRSISACGGGYSSKGSCGPC
As_Beta-6     MA------CVPQDCYSDI-CPRPYIDVCNSPCISSCGDSTAVVYAPPVVVRFPGPTMATCPQDSFVGS-SLPQLPAGSGGYPGVGGGVSGSLGSG-GYGGVYGGRFGGSSVGGFGGNFG-----GYR----GGYA------GGCGGGYSGGNGGSCGSRRSYRSISACGGGYSSKGSCGPC
As_Beta-8     MA------CVPQDCYSDI-CPRPYIDVCNSPCISSCGDSTAVVYAPPVVVRFPGPTMATCPQDSFVGS-SLPQLPAGSGGYPGVGGGVSGSLGSG-GYGGVYGGRFGGSSAGGFGGNFG-----GYR----GGYA------GGCGGGYSGGNGGSCGSRRSYRSISACGGGYSSKGSCGPC
Ps_Beta-51    MA------CVPQDCGSDI-CPRPYIDVCNSPCVSSCGDSTAVVFAPPVVVRFPGPTLATCPQDSIVGS-ALPQLPYGPGGFPGVGGGVGGPF-AG-GYGGVSGGRFGGNYGGYSGGSGG-----GYA-------A------GGCGGGYSGGYGGSCGSRRSYRSISSCGGGYSSKGGCGPC
Cp_Beta-B11   MSSCKDLSCRPSPCYPDI-CPDPCVVARNEPCITSCADSTAVVYPPPVSVLFPGPILSSSPQHSLVGS-TLPALPYGARGSFG-GGALGGPIGYGSGYGGALEGGYGYGGLSGYGGSYGYGGLSGYGGSYGYGGL------CGYGGGYGGGYGGLCGYGRRYGGRCYS----SRRGSCGPC
As_Beta-10    MSSRKELCCPRPQCYPDV-CPQPYVDAWNGPCVTSCGDSSAVVYPPPVVVRFPGPILATCPQESVVGT-ALPNVPYGSGGAYA-GGKFGSSVGSGGVY--------GS----------------------------------GYTGGYGAGYGGLFGDGSKYGRNCYS----SRFGGCGPC
Ps_Beta-11    MSSRKELCCPRPQCYPDV-CPQPYVDAWNGPCVTSCGDSSAVVYPPPVVVRFPGPILATCPQESVVGT-ALPNVPYGSGGAYA-GGKFGGSVSSGGVY--------GR----------------------------------GYTGGYGAGYGGLFGDGSKYGRNCYS----SRFGGCGPC
Pn_Beta-17    MSSSKDLCYPRPPCYPDI-CPDPYVDAWNEPCVTSCGDSSAVVYAPPVVVRFPGPILATCPQDSVVGS-TLPNLPYGYGGPYG-GGSFGGSVGSGGAYEGGYGARYGG----------------------------------GYGARYGGGYGGLYGYGKGYGRKCYS----SRFGSCGPC
Cp_Beta-B17   MSSSKALCYPRPPCYPDI-CPDPYVDACNEPCVTSCGDSSAVVYAPPVVVRFPGPILATCPQDSVVGS-TLPNLPYGYGGSYG-GGSFSGSVGSGGAY--------GG----------------------------------GYGAGYGGGYGGLYGYGKGYGRKCYS----SRFGSCGPC
Cp_Beta-B23   MSSSKALCYPRLPCHPDI-CPNPYVDAWNESCVTSCGDSSAVVYAPPVVVRFPGPILATCPQDSVVGS-TLPNLPYRYEGPYG-GGSFGGSGGSGRAY--------GG----------------------------------RYNVGYGSRYGDLCGHGRRFGRKCYS----SRFESCRPC

Consensus     M........s.cyp.c.!..P.P....cNePC!.sC.DS.vV!.ppPVvV.fPGPi$s..PQ.S.VG....p.vp.G.ggs.g.Gg..g.gg.yGg.yglg..ggygg..g.g...g..     ...    ....      .g...gy.g.ygg...yg...g............g.cgpc
```

Supplementary Figure S20. Alignment of amino acid sequences of turtle beta-keratins described in the present study and those investigated in previous gene expression studies. Amino acid sequences of beta-keratins from *Pseudemys nelsoni* (Ps, yellow) (Dalla Valle et al., 2009) and *Apalone spinifera* (As, grey) (Dalla Valle et al., 2013) were aligned with sequences of beta-keratins from *C. picta* (Cp, green) and *P. sinensis* (Ps, blue) (Supplementary Figures S1 and S2). The alignment was made with the Multalin algorithm. Red fonts, >90% conserved; blue fonts, >50% conserved.

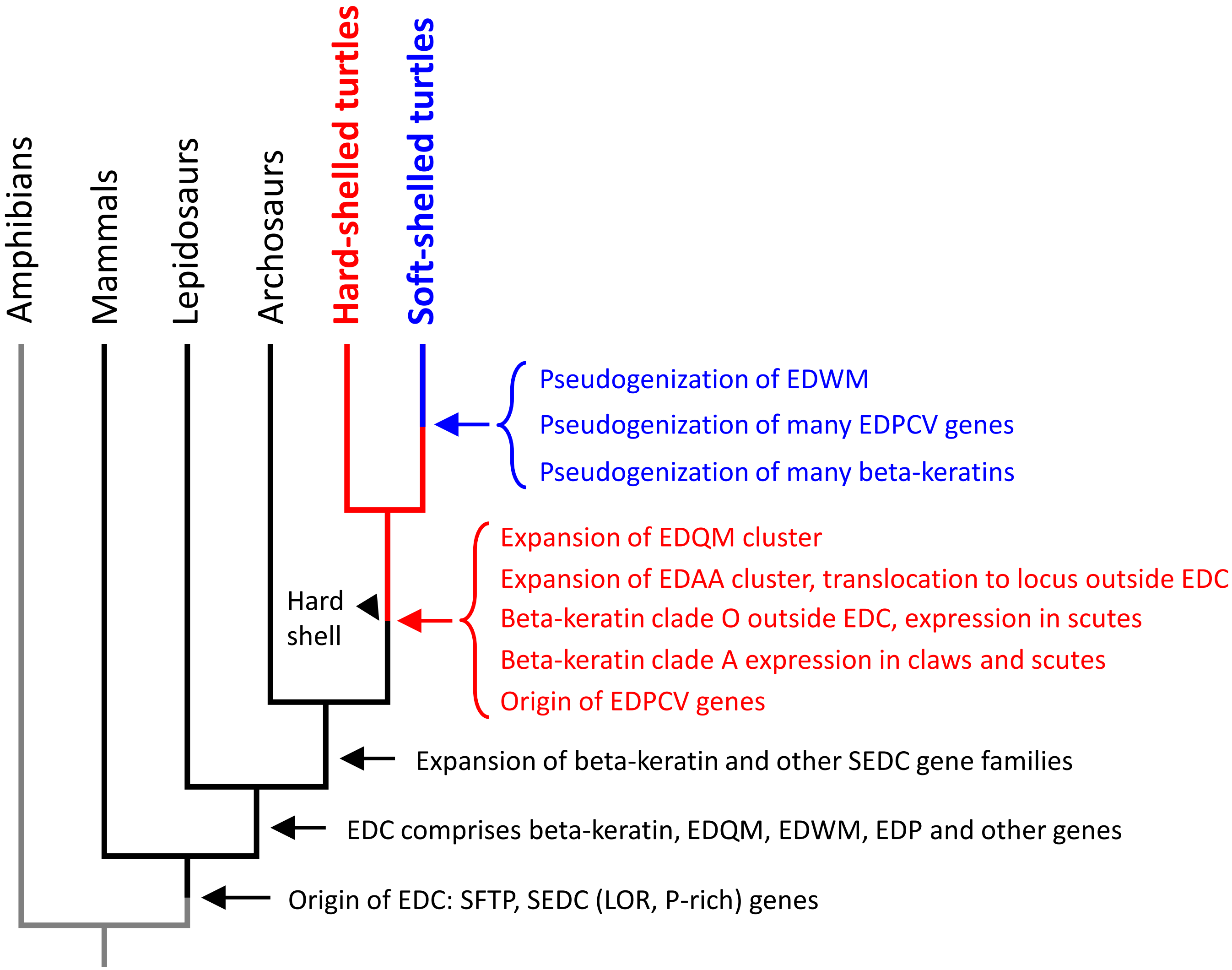

Supplementary Figure S21. Scenario for changes of the EDC during the evolution of soft-shelled turtles. The presence and absence of EDC genes in the various clades of terrestrial vertebrates (Strasser et al. 2014; this study) and application of the principle of parsimony was used to infer features of the EDC as well as gene origin and loss events during the evolution of turtles. Fossil evidence suggests that a hard shell was a basal trait in the evolution of all extant turtles (Gaffney, 1990; Li et al. 2008; Lyson et al. 2014). Abbreviations are explained in the main text. Note that the current model is built on data from a limited set of species. In-depth analyses of the EDC in further genomes, especially among turtles as well as birds and crocodilians (together indicated as Archosaurs), will allow refinements of this model.
